# Glycomic Analysis of Life Stages of the Human Parasite *Schistosoma mansoni* Reveals Developmental Expression Profiles of Functional and Antigenic Glycan Motifs*[Fn FN2]

**DOI:** 10.1074/mcp.M115.048280

**Published:** 2015-04-16

**Authors:** Cornelis H. Smit, Angela van Diepen, D. Linh Nguyen, Manfred Wuhrer, Karl F. Hoffmann, André M. Deelder, Cornelis H. Hokke

**Affiliations:** From the ‡Department of Parasitology, Center of Infectious Diseases, Leiden University Medical Center, 2333 ZA Leiden, The Netherlands;; §Center for Proteomics and Metabolomics, Leiden University Medical Center, 2333 ZA Leiden, The Netherlands;; ¶Institute of Biological Environmental and Rural Sciences (IBERS), Aberystwyth University, Penglais Campus, Aberystwyth SY23 3FG, United Kingdom

## Abstract

Glycans present on glycoproteins and glycolipids of the major human parasite *Schistosoma mansoni* induce innate as well as adaptive immune responses in the host. To be able to study the molecular characteristics of schistosome infections it is therefore required to determine the expression profiles of glycans and antigenic glycan-motifs during a range of critical stages of the complex schistosome lifecycle. We performed a longitudinal profiling study covering schistosome glycosylation throughout worm- and egg-development using a mass spectrometry-based glycomics approach. Our study revealed that during worm development N-glycans with Galβ1–4(Fucα1–3)GlcNAc (LeX) and core-xylose motifs were rapidly lost after cercariae to schistosomula transformation, whereas GalNAcβ1–4GlcNAc (LDN)-motifs gradually became abundant and predominated in adult worms. LeX-motifs were present on glycolipids up to 2 weeks of schistosomula development, whereas glycolipids with mono- and multifucosylated LDN-motifs remained present up to the adult worm stage. In contrast, expression of complex *O*-glycans diminished to undetectable levels within days after transformation. During egg development, a rich diversity of *N*-glycans with fucosylated motifs was expressed, but with α3-core fucose and a high degree of multifucosylated antennae only in mature eggs and miracidia. *N*-glycan antennae were exclusively LDN-based in miracidia. *O*-glycans in the mature eggs were also diverse and contained LeX- and multifucosylated LDN, but none of these were associated with miracidia in which we detected only the Galβ1–3(Galβ1–6)GalNAc core glycan. Immature eggs also exhibited short *O*-glycan core structures only, suggesting that complex fucosylated *O*-glycans of schistosome eggs are derived primarily from glycoproteins produced by the subshell envelope in the developed egg. Lipid glycans with multifucosylated GlcNAc repeats were present throughout egg development, but with the longer highly fucosylated stretches enriched in mature eggs and miracidia. This global analysis of the developing schistosome's glycome provides new insights into how stage-specifically expressed glycans may contribute to different aspects of schistosome-host interactions.

Schistosoma blood flukes give rise to infections in over 200 million people in developing countries worldwide (1). With a Disability-Adjusted Life Years (DALY) value of more than 3 million, schistosomiasis ranks as one of the neglected tropical diseases with the highest impact on public health (2). The schistosome has a complex and intriguing lifecycle, which involves a definitive host (mammal) as well as an intermediate host (snail). Infections with *Schistosoma mansoni*, one of the major schistosome species infecting humans, are initiated when snail-borne cercariae penetrate intact skin. The cercariae then transform into schistosomula, which enter the vasculature of the host and mature while migrating to the portal system. Here, adult male and female worms pair, with the female worm producing hundreds of eggs each day during a life span of several years unless the infection is treated by chemotherapy. Miracidia develop inside the maturing eggs while they cross the intestinal wall over a period of several days to be excreted with the feces. Miracidia then hatch from the eggs upon contact with fresh water and infect the snail host where asexual replication takes place and eventually new cercariae are shed. Notably, many eggs get trapped in organs such as the liver, where they induce a granulomatous inflammation and organ damage, the main cause of pathology in schistosomiasis (1).

Throughout their lifecycle, schistosomes express a multitude of protein- and lipid-linked glycans that play an important role in the parasite biology. The expression of many glycan elements appears to be developmentally regulated by the differential expression of glycosyltransferases during the different lifecycle stages (3). A series of papers has been published indicating that schistosome glycans play essential roles in the molecular interaction of the parasite and the host immune system, enabling survival of the parasite and allowing chronic infection to establish. For example, glycosylated soluble egg antigens (SEA) interact with the C-type lectins mannose receptor (MR), macrophage galactose-type lectin (MGL) and dendritic cell-specific ICAM-3-grabbing nonintegrin (DC-SIGN), and some of these interactions lead to immunomodulatory effects of specific components of SEA via dendritic cells (DCs)^1^ (4, 5). Furthermore, fucosylated egg glycolipids trigger innate immune responses of peripheral blood mononuclear cells and egg glycans are required for periovular granuloma formation in a mouse model. In addition, cercarial secretions induce alternatively activated macrophages in a carbohydrate dependent manner (6–9). Importantly, also adaptive immune responses to schistosome glycans are mounted by the human host. A large part of the antibody responses to schistosomes is directed against antigenic glycan motifs, raising the question whether they could form a basis for antischistosome vaccine strategies (10).

Rapid developments in mass spectrometry-based glycan-analysis technology in the last two decades have led to several studies focused on elucidating the glycan structures of somatic and secretory schistosome preparations (11–22). Among the typical glycan elements detected in *S. mansoni* were unusual and antigenic Fucα1–2Fucα1–3- (DF-) motifs attached to GalNAcβ1–4GlcNAc (LacDiNAc or LDN) (12, 14, 17–19, 21), Xylβ1–2- and Fucα1–3-modified *N*-glycan core structures (13, 15, 17, 20), and a unique *O*-glycan core (Galβ1–3(Galβ1–6)GalNAc) (14, 17) (see supplemental Table S5 for a definition of glycan motifs of *S. mansoni* glycoconjugates). Also more widely occurring glycan elements shared with the mammalian or snail host were detected, *e.g.* Galβ1–4GlcNAc (LacNAc or LN), Galβ1–4(Fucα1–3)GlcNAc (Lewis X or LeX), LDN, and GalNAcβ1–4(Fucα1–3)GlcNAc (LDN-F) (23, 24). These data were generated over a long period of time, often focusing on a single schistosome life stage and a specific class of glycans only, and using various analytical techniques and strategies that make inter-study comparisons often difficult. In addition, glycosylation of the schistosomula that develop shortly after infection and are considered to be relatively vulnerable to immune attack, has remained largely unexplored (20, 25, 26), although these could be interesting therapeutic targets (27–29). Clearly, an integrated and complete overview of schistosome glycosylation was so far not available.

In this study, we therefore set out to determine the overall schistosome protein- and lipid-linked glycome by analyzing a total of 16 lifecycle stages ranging from cercariae to miracidia. We analyzed the glycoprotein-derived *N*- and *O*-glycans as well as the lipid-derived glycans of these life stages by a MALDI-TOF MS-based approach complemented with fragmentation and enzyme degradation studies. Our findings give new insights in the glycobiology of parasite development and parasite–host interaction and contribute to the identification of new potential immune intervention targets.

## EXPERIMENTAL PROCEDURES

### 

#### 

##### Glycoprotein and Glycolipid Preparations

Puerto Rican-strains of *S. mansoni* maintained in the laboratories of LUMC and Aberystwyth University were used throughout this study. For the *N*-glycan analysis the protein/glycoprotein samples were obtained from the various life stages by TRIzol (Invitrogen, Paisly, UK) extracted homogenates generated as described before (3). Parasite preparations for *O*-glycan and lipid-glycan release were obtained separately as follows. Cercariae collected from *Biomphalaria glabrata* snails were transferred to prewarmed (37 °C) medium199 (Gibco Life Technologies, Bleiswijk, The Netherlands) containing 10 mm HEPES (Sigma-Aldrich, Zwijndrecht, The Netherlands), 1× antibiotic antimycotic solution (Sigma-Aldrich) and 15 μm l-glutamine (Sigma-Aldrich) to induce transformation. After 20 min incubation at 37 °C and 5% CO_2_ schistosomula were separated from loose tails by orbital shaking. Schistosomula were collected and cultured for 3, 24, 48, and 72 h at 37 °C and 5% CO_2_ in the same medium or for 9 days in this medium supplemented with erythrocytes (10 μl of packed erythrocytes/200 μl medium, refreshed every 24 h). Adult worms and eggs were obtained from hamsters 7 weeks after infection, as described (30). Mature and immature eggs were isolated by Percoll gradient centrifugation (31). Miracidia were obtained from eggs hatched in distilled water and collected as previously described (30). All parasite isolates were subjected to extraction by potter homogenization in chloroform, methanol (MeOH), and water (13:7:4). The upper phase of the extraction was removed after sonication and centrifugation and replaced by the same volume of 50% MeOH. These steps were repeated twice, however, after the last removal of the upper phase the proteins were pelleted by adding an excess amount of 100% MeOH and centrifugation. Pellets were washed three times with MeOH, dried under a flow of nitrogen, and used for *O*-glycan release. For glycolipid isolation, the combined upper phases from the chloroform-methanol extraction procedure were applied to reversed phase (RP) C18-cartridges (500 mg; JT Baker, Phillipsburg, NJ). Combined flow-through and wash fractions (10 ml water) were applied to a second C18-cartridge, and glycolipids were eluted from both cartridges with 5 ml chloroform/MeOH/water (10:10:1) and dried combined under a flow of nitrogen.

##### N-glycan Release

Protein extracts were potter homogenized in PBS with 1.3% SDS and 0.1% β-mercaptoethanol. Homogenized proteins were denatured at 60 °C for 10 min. After cooling down, 1.3% Nonidet P-40 Substitute (Sigma-Aldrich) was added. *N*-glycans except those with Fucα1–3 modified core structures, were released by adding PNGase F (17 mU, Roche Diagnostics, Almere, The Netherlands) and incubation for 24 h at 37 °C. To isolate released glycans, the incubation mixture was applied to a C18 RP-cartridge. The combined flow-through and wash fraction was subsequently applied to a carbon cartridge (150 mg Carbograph; Grace, Deerfield, IL), washed with milliQ, and glycans were eluted with subsequent addition of 25% acetonitrile (AcN) and 50% AcN/0.1% trifluoroacetic acid (TFA). Proteins remaining on the C18-cartridge were retrieved by applying 30% AcN containing 0.1% TFA followed by 60%AcN/0.1%TFA and dried. The protein fraction was dissolved in PBS and incubated overnight at 37 °C while shaking in the presence of trypsin-coated Sepharose, prepared following the manufacturers protocol (GE Healthcare, Diegem, Belgium), to create tryptic peptides/glycopeptides. The supernatants were removed, dried, and taken up in 1 m sodium acetate pH 4.5, before addition of PNGase A (0.5 mU; Roche Diagnostics). Digestion was continued for 48 h at 37 °C and the PNGase A released glycans were purified as described for the PNGase F-released glycans.

##### O-glycan Release

Protein pellets obtained after chloroform–methanol extraction of parasite homogenates were subjected to reductive β-elimination. The dried samples were taken up in 1 m sodium borohydride, 0.1 m sodium hydroxide, and incubated at 40 °C for 24 h. Samples were neutralized on ice by adding 4 m acetic acid. MeOH/1% acetic acid was added repeatedly with intermediate and final drying under a stream of nitrogen. Released glycans were taken up in milliQ and applied to RP C18-cartridges as described before. Combined flow-through and wash fractions were applied to a carbon cartridge. After a wash with 6 ml water, glycans were eluted with 3 ml 25% AcN and 3 ml 25% AcN/0.05% TFA. To facilitate MALDI-TOF-MS analyses, the purified *O*-glycan alditols were permethylated according to (32). In short, glycans were lyophilized and 100 μl DMSO saturated with sodium hydroxide-powder was added. The samples were kept for 10 min at room temperature, 100 μl iodomethane was added followed by 10 min incubation at room temperature. Dichloromethane (400 μl) and water (500 μl) were added and samples were shaken by multiple inversions, followed by removal of the aqueous-layer and addition of another 500 μl of water. After the fifth removal of the aqueous-layer, the remaining, washed organic layer containing the permethylated glycans was dried under a flow of nitrogen. For further analysis, permethylated *O*-glycans were dissolved in 80% MeOH.

##### Lipid Glycan Release

Glycolipid extracts were dissolved in 50 mm sodium acetate pH 5 containing 0.1% sodium taurodeoxycholate hydrate (Sigma-Aldrich). The sample was sonicated, heated to 50 °C for 10 min, and then incubated at 37 °C with 16 mU recombinant endoglycoceramidase II from *Rhodococcus* sp. (rEGCase II) (Takara-Bio, Otsu, Japan) for 48 h. After 24 h of incubation another 16 mU rEGCase II was added. Released glycans were purified using RP C18- and carbon cartridges as described above for *N*-glycans.

##### Glycan Labeling

Released reducing glycans were labeled with 2-aminobenzoic acid (2-AA), as described previously (33). Glycans were taken up in 50 μl milliQ and 50 μl of labeling mix (DMSO/acetic acid (10:3) containing 48 mg/ml 2-AA (Sigma-Aldrich) and 107 mg/ml picoline-borane-complex (Sigma-Aldrich)) was added. Samples were incubated at 65 °C for 2 h. Labeled glycans were taken up in 75% AcN and were loaded on Biogel P10 (BIO-RAD, Veenendaal, The Netherlands) conditioned with 80% AcN. The Biogel was washed with 80% AcN and glycans were eluted with milliQ.

##### Exoglycosidase Digestions

Selected glycan pools were treated with specific exoglycosidases to support glycan structure assignments. Each enzyme reaction mixture contained a 1 μl aliquot of labeled glycans, 6 μl 100 mm sodium phosphate buffer pH 5 and 5 μl of either one of the following enzymes: β-*N*-acetylglucosaminidase from jack bean (NAG; 312.5 mU)(Sigma-Aldrich), α-l-fucosidase from bovine kidney (BKF; 33.3 mU)(Sigma-Aldrich), α(1–3,4)-fucosidase from *Xanthomonas manihotis* (XMF; 2.5 mU)(Sigma-Aldrich). For treatment with β(1–4,6)-galactosidase from jack bean (JBG) (227.3 mU) (Prozyme, Hayward, CA) 250 mm sodium citrate pH 4.0 was used. All exoglycosidase digestions were performed at 37 °C for 24 h.

##### Immunofluorescence Microscopy

Cercariae and 3-day-old schistosomula were washed with PBS and fixed with 2% paraformaldehyde. Fixed parasites were suspended in 100 μl of undiluted hybridoma culture supernatant containing either of the monoclonal antibodies 291–4D10-A and 114–5B1-A, recognizing LeX- and LD*N*-DF-motifs, respectively (34). After 30 min incubation at 37 °C, parasites were washed with PBS and suspended in 100 μl of a 1:200 dilution of Alexa Fluor 488-conjugated goat-anti-mouse IgG (Life Technologies) followed by 30 min incubation at 37 °C. Parasites were then washed with PBS, transferred to a glass-slide and analyzed by fluorescence microscopy (Leica DM-RB (Leica, Rijswijk, The Netherlands)).

##### MALDI-TOF-MS

2-AA-labeled glycans were analyzed by MALDI-TOF-MS in the negative-ion reflectron mode with 2,5-dihydroxybenzoic acid (DHB, 20 mg/ml in 30% AcN, Bruker Daltonics, Bremen, Germany) as matrix using Ultraflex II and Ultraflextreme mass spectrometers (Bruker Daltonics). All recorded spectra were obtained as summed spectra of 20,000 shots. Exoglycosidase treated glycans were purified from the digestion mixture using ZipTip C18 (Millipore, Amsterdam, The Netherlands) following the manufacturer's instructions and eluted directly onto the MALDI-target plate with 10 mg/ml DHB in 50% AcN containing 0.1% TFA. Permethylated *O*-glycans were analyzed in positive-ion reflectron mode using DHB as matrix. Fragmentation ion analysis was performed using the LIFT-MS/MS facility of the Ultraflex II and Ultraflextreme mass spectrometers. Precursor ions were selected in a timed ion gate after 8 kV acceleration, and laser-induced fragment ions were further accelerated with 16 kV in the LIFT cell and fragment masses were registered.

##### Interpretation of Mass Spectra, Search Parameters, and Assignment Criteria

The aim of this study was to analyze and compare in a semiquantitative manner the expression of the dominant glycans and glycan motifs during different life stages of *S. mansoni.* Therefore, we have limited spectral assignments to the relatively abundant signals only, and whenever possible we used previously published *S. mansoni* glycan structural data as a basis for interpretations. Mass spectra were smoothed and base-line subtracted using FlexAnalysis version 3.3 (Bruker Daltonics) and peak lists were used as input for subsequent identification of the glycan compositions in Glycopeakfinder (http://www.glyco-peakfinder.org) (35). Peaks with a signal to noise ratio below 3 were excluded from this analysis. For *N*- and lipid-glycans, 2-AA was taken into account as a fixed reducing-end modification and deprotonated masses were registered. *O*-glycans were reduced and permethylated and masses were registered as sodium adducts. Possible glycan compositions were allowed to have 0–20 residues of deoxyhexose, hexose and *N*-acetylhexosamine each for all glycan classes and, in addition, 0–1 pentose for the *N*-glycans. All compositions that matched with registered masses with a deviation of less than 500 ppm were further evaluated manually to assign glycan structures and consider possible isobaric and isomeric structures on the basis of common or previously published specific knowledge of schistosome glycans (11–22, 36–38). In specific cases, proposed structures were confirmed by exoglycosidase treatment (*N*-glycans) and fragmentation analysis (*O*- and lipid-glycans) as described in the results section. In the MALDI-TOF-MS spectra of *N*-glycans and glycolipids only the structure of the most likely or most abundant isomer is indicated for each composition. In case of the permethylated *O*-glycans, minor signals caused by under-methylation were not annotated.

##### Quantification

This study was primarily designed as a qualitative assessment of developmental glycosylation of schistosomes. For each mass spectrum of PNGase F-sensitive glycans, the relative quantity of a number of relevant glycan types and motifs within the overall glycan set of that life stage was estimated from the summed area of the two highest isotope signals for each glycan as a percentage of the total area of all glycan-signals in the spectrum. Absolute quantities and the relative ratios of the *N*-, *O*-, and glycolipid-derived glycan classes could not be determined in this study, but a low signal to noise ratio was interpreted as an indication for general low abundance of the specific glycan class in the life stage examined, or for absence if glycan derived signals were undetectable. Qualitative reproducibility of the data was confirmed by comparing profiles of the different glycan classes derived from biological duplicates or triplicates of cercariae, adult worms, and eggs, and in addition by comparison with published data as indicated in the Results section.

## RESULTS

Overviews of protein- and lipid-linked glycosylation of up to 16 *S. mansoni* life stages were generated. Protein extracts of all life stages studied were subjected to digestion with PNGase F, followed by digestion with PNGase A to release PNGase F resistant *N*-glycans with a Fucα1–3-modified core (core(α3)-Fuc), if present (13, 15, 17, 20). *O*-glycans and lipid-glycans were obtained from additional protein and lipid extracts by reductive β-elimination and by digestion with endo-glycoceramidase, respectively. The reducing *N*-glycans and glycolipid-glycans were derivatized with 2-AA, *O*-glycan alditols were permethylated, and all glycans were subsequently analyzed by MALDI-TOF-MS. Resulting data for *N*-glycans, *O*-glycans, and lipid-glycans will be presented and discussed in relation to worm development and egg maturation.

### 

#### 

##### N-glycans

**Fig. 1.**
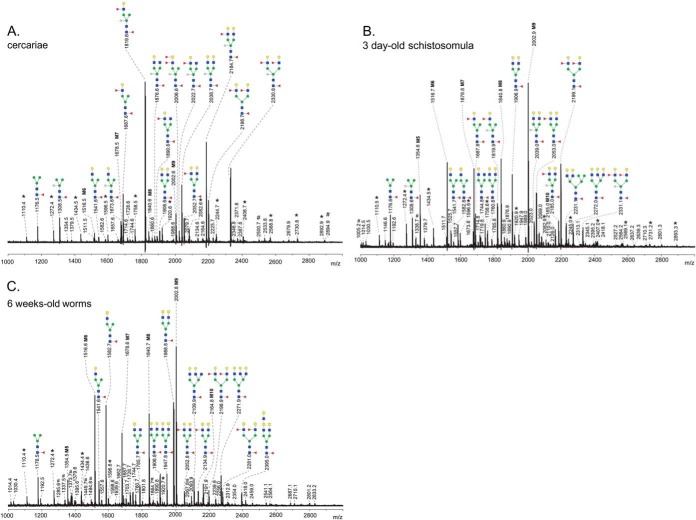
**MALDI-TOF-MS of N-glycans released by PNGase F from *S. mansoni* cercariae *A*, 3 day schistosomula, *B*, and 6 week worms, *C*.** The 2-AA-labeled glycans were analyzed in negative-ion reflectron mode. All signals are labeled with monoisotopic masses and structures were deduced from these masses on the bases of exoglycosidase digestions and published data. Red triangle, fucose; yellow circle, galactose; blue square, *N*-acetylglucosamine; green circle, mannose; yellow square, *N*-acetylgalactosamine; white star, xylose. M5-M9 oligomannosidic *N*-glycans with five to nine mannose residues. M10, oligomannosidic *N*-glycan with nine mannose and one glucose residue. Signals corresponding to a hexose oligomer of unknown origin are marked with *. Nonglycan signals are marked with #.

**Fig. 2.**
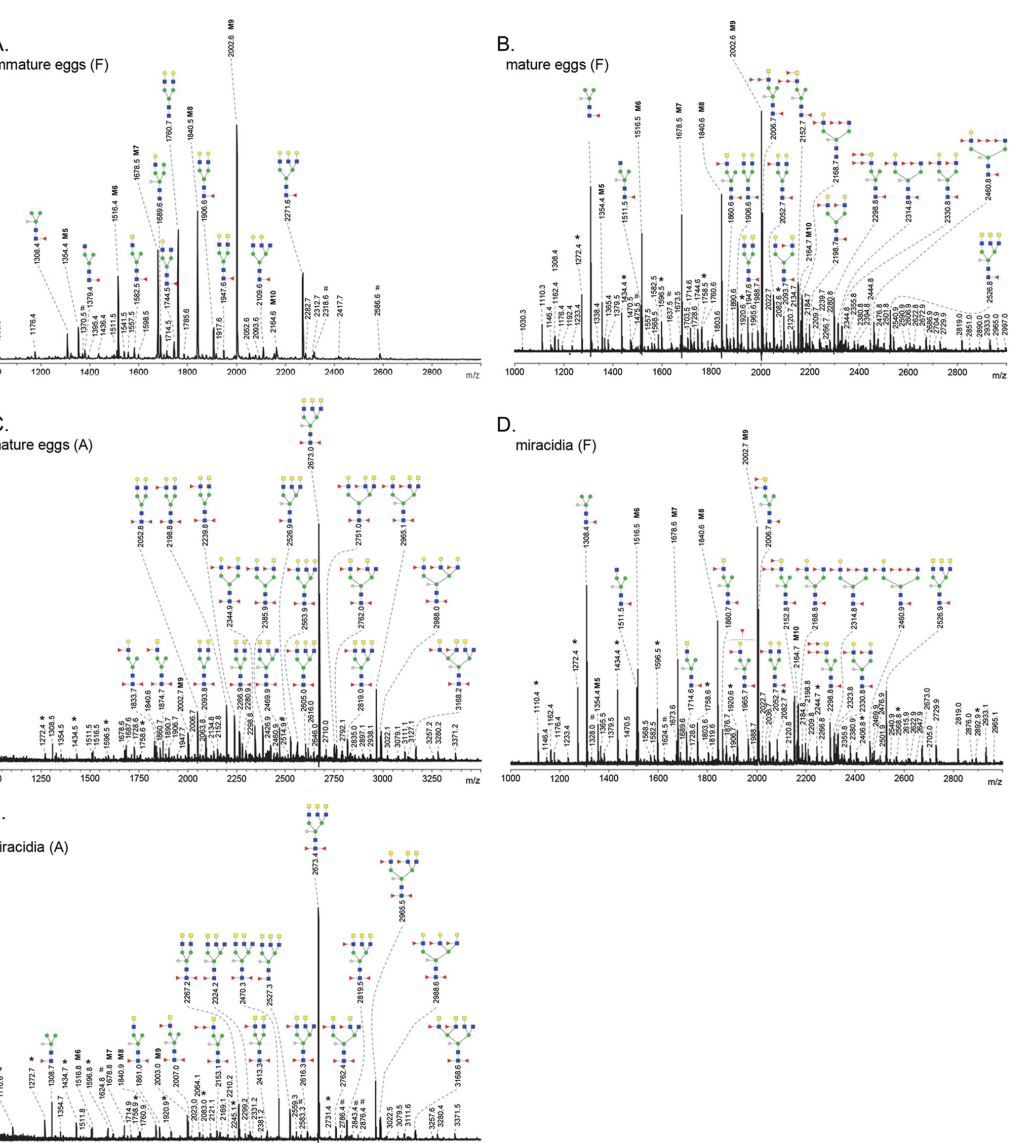
**MALDI-TOF-MS of *N-*glycans released by PNGase F from *S. mansoni* immature eggs, *A*, mature eggs, *B*, and miracidia, *D*, and the PNGase A-sensitive *N-*glycans of mature eggs, *C*, and miracidia, *E*.** The 2-AA-labeled glycans were analyzed in negative-ion reflectron mode. All signals are labeled with monoisotopic masses and structures were deduced from these masses on the bases of exoglycosidase digestions and published data. Red triangle, fucose; yellow circle, galactose; blue square, *N*-acetylglucosamine; green circle, mannose; yellow square, *N*-acetylgalactosamine; white star, xylose. M5–M9 oligomannosidic *N*-glycans with five to nine mannose residues. Signals corresponding to a hexose oligomer of unknown origin are marked with *. Nonglycan signals are marked with #.

*N*-glycans released by PNGase-F were detected in all life stages studied. During the development from cercariae to adult worms, *N*-glycan profiles of consecutive stages overlap, displaying mainly changes in the relative peak intensities, but ultimately worm maturation leads to the appearance of adult worm-specific glycans in addition to the complete disappearance of specific larval *N*-glycans. Sharply different *N*-glycomes are observed between the worm and egg stages (Fig. 1*A*–1*C*, supplemental Fig. S1*A*–S1*J*, and Fig. 2*A*, 2*B*, 2*D*). After cleavage of the PNGase F-sensitive *N*-glycans, we performed a subsequent digestion with PNGase A for each life stage. In line with previous reports (13, 17, 20), core(α3)-Fuc modified *N*-glycans were not detectable in the cercariae to adult worm range, whereas PNGase A treatment of mature egg and miracidia glycoproteins gave rise to an abundant set of core(α3)-Fuc substituted *N*-glycans with unique compositions and structures compared with the PNGase F released *N*-glycans (Fig. 2*C*, 2*E* compared with Fig. 2*B*, 2*D*).

**Fig. 3.**
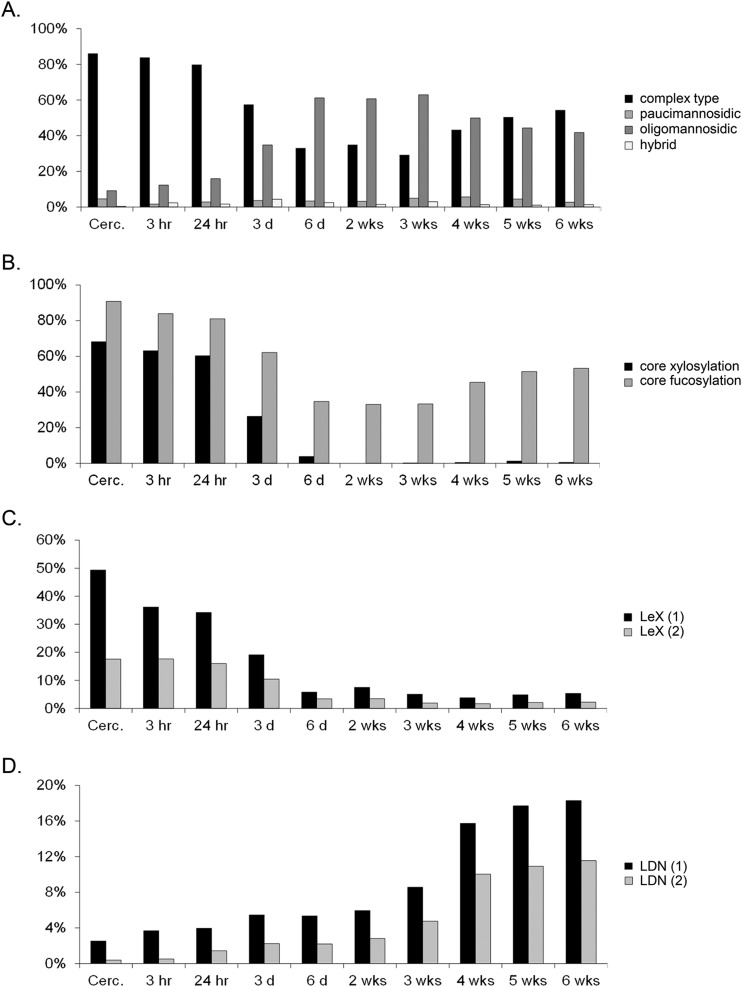
**Expression levels of different *N-*glycan classes *A*, and glycan motifs (*B–D*) among the PNGase F-sensitive *N-*glycans during *S. mansoni* worm development.** Glycan motifs shown are core-xylose and core(α6)-Fuc, *B*, LeX-antennae, either one (1; dark bars) or two (2; light bars) in a single glycan, *C*, and LDN-antennae, either one (1; dark bars) or two (2; light bars) in a single glycan, *D*.

To facilitate the assignment of all *N*-glycan spectra recorded we first interpreted and validated *N*-glycan spectra of cercariae, 3-day-old schistosomula, 6-week-old worms and mature eggs to serve as a template for annotation of the spectra obtained for the other stages. For the purpose of relative quantification of selected *N*-glycan elements during worm development (Fig. 3) we assigned the 20 most intense glycan-derived signals in each spectrum, complemented with the next most abundant signals to cover up to 95% of the area of all glycan signals in the spectrum, except when the area of the individual signals was less than 1% of the total.

##### N-glycans of Cercariae

The *N*-glycan spectrum of cercariae (Fig. 1*A*) is highly similar to the previously published cercarial *N*-glycan spectra (15, 20). Dominant are the mono- or di-antennary glycans with Fucα1–6- and Xylβ1–2-modified core structures (core(α6)-Fuc and core-Xyl, respectively) and the antenna motifs LN and/or LeX. Representative signals derived from glycans with the composition X_1_F_1–2_H_4_N_3_ (X, xylose, Xyl; F, fucose, Fuc; H, hexose, Hex; N, *N*-acetylhexosamine, HexNAc) were observed at *m*/*z* 1673.6 [M-H]^−^ and *m*/*z* 1819.6 [M-H]^−^, whereas signals derived from X_1_F_1–3_H_5_N_4_ were found at *m*/*z* 2038.7 [M-H]^−^, 2184.7 [M-H]^−^, and *m*/*z* 2330.8 [M-H]^−^. The presence of core-fucose within all of these structures was confirmed by digestions with XMF. Under the conditions applied, XMF cleaves of Fuc in the context of LeX, but not core-Fuc (36, 39). After digestion, abovementioned peaks shifted because of the loss of up to two LeX-associated Fuc-residues to *m*/*z* values corresponding to the presence of one remaining core(α6)-Fuc (*i.e.* X_1_F_1_H_4_N_3_ at *m*/z 1673.4 [M-H]^−^ and X_1_F_1_H_5_N_4_ at *m*/*z* 2038.5 [M-H]^−^ (Table I). All other cercarial *N*-glycans observed also carry core(α6)-Fuc, except the oligomannose glycans. Complementary proof of the localization of Fuc-residues was obtained by digestion with JBG, which cleaves off terminal Gal-residues in the context of LN, but not of LeX. Original signals shifted in the JBG digestion-spectrum because of the loss of one or two Gal residues to *m*/*z*-values corresponding to X_1_F_1_H_3_N_3_ at *m*/*z* 1511.4 [M-H]^−^, X_1_F_1_H_3_N_4_ at *m*/*z* 1714.5 [M-H]^−^, and X_1_F_2_H_4_N_4_ at *m*/*z* 2022.6 [M-H]^−^, whereas X_1_F_2_H_4_N_3_ at *m*/*z* 1819.5 [M-H]^−^ and X_1_F_3_H_5_N_4_ at *m*/*z* 2330.7 [M-H]^−^ did not shift because of the presence of one and two LeX antenna, respectively (Table I). In addition, a set of similar glycans, but lacking the core-xylose was found in the original spectrum at *m*/*z* 1541.6 [M-H]^−^ (F_1_H_4_N_3_), 1687.6 [M-H]^−^ (F_2_H_4_N_3_), 1906.6 [M-H]^−^ (F_1_H_5_N_4_), 2052.7 [M-H]^−^ (F_2_H_5_N_4_), and *m*/*z* 2198.7 [M-H]^−^ (F_3_H_5_N_4_). Structures were confirmed by exo-glycosidase treatment in the same way as for the xylosylated series (Table I). Oligomannosidic glycans (H_6–9_N_2_), indicated by signals at *m*/*z* 1516.5 [M-H]^−^, 1678.5 [M-H]^−^, 1840.6 [M-H]^−^, and 2002.6 [M-H]^−^, and paucimannosidic glycans detected at *m*/*z* 1308.5 [M-H]^−^and 1176.5 [M-H]^−^ (X_1_F_1_H_3_N_2_ and F_1_H_3_N_2_ respectively) were observed at low levels relative to the complex-type glycans. Furthermore, relatively minor signals were observed representing glycans with one LN and one truncated antenna (*e.g. m*/*z* 2022.7 [M-H]^−^ (X_1_F_2_H_4_N_4_), 1876.6 [M-H]^−^ (X_1_F_1_H_4_N_4_), and 1890.6 [M-H]^−^ (F_2_H_4_N_4_)) or with a fucosylated LDN-antenna (*e.g.* 2006.6 [M-H]^−^ (X_1_F_3_H_3_N_4_)). Glycans carrying either GlcNAc or LDN-antennae were affected by NAG treatment, which cleaves off all unsubstituted HexNAc residues (Table I). It is not possible to discriminate between an unsubstituted LDN disaccharide unit, or two single terminal GlcNAc residues on the basis of NAG treatment, but HexNAc_2_-fragments indicative for the presence of LDN have previously been detected as part of the *N*-glycans of cercariae (15) and we therefore assume that two HexNAc residues in the context of an *N*-glycan core form a single LDN-antenna. Finally, in cercariae we did not find indications for the presence of tri- or tetra-antennary glycans, DF-motifs, and core(α3)-Fuc. These observations are in accordance with previous reports (15, 20).

**Table I:** Overview of exoglycosidase digestions of S.mansoni cercariae PNGase F-sensitive N-glycans and structures deduced

Registered masses*^a^*	Putative composition*^b^*	Jackbean N-acetylhexosaminidase treatment*^c^*	Jackbean beta-Galactosidase treatment*^c^*	Bovine kidney fucosidase treatment*^c^*	Xanthomonas manihotis fucosidase treatment*^c^*	Most probable structural characteristics*^d^*
1176.5	F1H3N2	-	-	1030 (-1F)	-	Core fucosylation
1308.5	X1F1H3N2	-	-	1162 (-1F)	-	Core fucosylation; core xylosylation
1354.5	H5N2	-	-	-	-	Oligomannose
1516.5	H6N2	-	-	-	-	Oligomannose
1541.6	F1H4N3	-	1379 (-1H)	1395 (-1F)	-	1 LacNAc; core fucosylation
1673.6	X1F1H4N3	-	1511 (-1H)	1527 (-1F)	-	1 LacNAc; core fucosylation; core xylosylation
1678.5	H7N2	-	-	-	-	Oligomannose
1687.6	F2H4N3	-	-	1395 (-2F)	1541 (-1F)	1 Lewis X; core fucosylation
1819.6	X1F2H4N3	-	-	1527 (-2F)	1673 (-1F)	1 Lewis X; core fucosylation; core xylosylation
1840.6	H8N2	-	-	-	-	Oligomannose
1876.6	X1F1H4N4	1673 (-1N)	1714 (-1H)	1730 (-1F)	-	1 single HexNAc; 1 LacNAc; core fucosylation; core xylosylation
1890.6	F2H4N4	1687 (-1N)	1728 (-1H)	1598 (-2F)	1744 (-1F)	1 single HexNAc; 1 Lewis X; core fucosylation
1906.6	F1H5N4	-	1744 (-1H)/1582 (**-**2H)	1760 (-1F)	-	2 LacNAc; core fucosylation
2002.6	H9N2	-	-	-	-	Oligomannose
2006.6	X1F3H3N4	-	-	1860 (-1F)/1714 (-2F)/1568 (-3F)	1714 (-2F)	1 F-LDN-F or 2 single fucosylated HexNAc; core fucosylation; core xylosylation
2022.7	X1F2H4N4	1819 (-1N)	1860 (-1H)	1730 (-2F)	1876 (-1F)	1 single HexNAc; 1 Lewis X; core fucosylation; core xylosylation
2038.7	X1F1H5N4	-	1876 (-1H)/1614 (-2H)	1892 (-1F)	-	2 LacNAc; core fucosylation; core xylosylation
2052.7	F2H5N4	-	1890 (-1H)	1760 (-2F)	1906 (-1F)	1 LacNAc; 1 Lewis X; core fucosylation
2164.6	H10N2	-	-	-	-	Oligomannose
2184.7	X1F2H5N4	-	2022 (-1H)	1892 (-2F)	2038 (-1F)	1 LacNAc; 1 Lewis X; core fucosylation; core xylosylation
2198.7	F3H5N4	-	-	1760 (-3F)	1906 (-2F)	2 Lewis X; core fucosylation
2330.8	X1F3H5N4	-	-	1892 (-3F)	2038 (-2F)	2 Lewis X; core fucosylation; core xylosylation

*^a^* Registered mass in the undigested spectrum

*^b^* X, Xylose; F, Fucose; H, Hexose; N, N-acetylhexosamine

*^c^* Resulting masses after digestion of the original mass with the different exoglycosidases. Changes in monosaccharides are indicated in parenthesis. -, no shift in mass after exoglycosidase digestion.

*^d^* Core xylosylation was assigned on the basis of composition.

##### N-glycans of 3-Day-Old Schistosomula

The *N*-glycan spectrum of 3-day-old schistosomula is shown in Fig. 1*B*. In line with previous findings (20), the major signals are attributable to oligomannosidic structures (*i.e.* H_5–10_N_2_ at *m*/*z* 1354.6 [M-H]^−^, 1516.7 [M-H]^−^, 1678.8 [M-H]^−^, 1840.8 [M-H]^−^, 2002.9 [M-H]^−^, and 2165.0 [M-H]^−^). Signals corresponding to core(α6)-fucosylated mono- and di-antennary glycans with LN and/or LeX antennae but without core-Xyl, were also relatively abundant at *m*/*z* 2199.1 [M-H]^−^ (F_3_H_5_N_4_), 1906.9 [M-H]^−^ (F_1_H_5_N_4_), 2053.0 [M-H]^−^ (F_2_H_5_N_4_), 1687.8 [M-H]^−^ (F_2_H_4_N_3_), 2272.1 [M-H]^−^ (F_1_H_6_N_5_), 1541.7 [M-H]^−^ (F_1_H_4_N_3_), and 2231.1 [M-H]^−^ (F_1_H_7_N_4_). Guided by the exoglycosidase treatments (supplemental Table S1), this F_1_H_7_N_4_-species was assigned to a hybrid glycan with a tandem repeat LN-antennae. Signals derived from additional hybrid glycans (*e.g.* at *m*/*z* 1719.8 [M-H]^−^ (H_6_N_3_) and 2069.0 [M-H]^−^ (F_1_H_6_N_4_)) were observed at low abundance. The F_1_H_6_N_5_-species at *m*/*z* 2272.1 [M-H]^−^ represents a tri-antennary structure (supplemental Table S1). Structures with a core(α6)-Fuc and LeX/LN-antennae together with the core-Xyl modification (*i.e.* X_1_F_2_H_4_N_3_ at *m*/*z* 1819.9 [M-H]^−^ and X_1_F_1–3_H_5_N_4_ at *m*/*z* 2039.0 [M-H]^−^, 2185.0 [M-H]^−^, and 2331.1 [M-H]^−^) were found at much lower levels than in cercariae. Similar to cercariae, *N*-glycans expressing truncated antennae and LDN-antennae (F_1_H_3–4_N_4_ at *m*/*z* 1582.8 [M-H]^−^ and 1744.8 [M-H]^−^) were observed at low levels.

##### N-glycans of 6-Week-Old Adult Worms

The most abundant glycans detected in the adult worm *N*-glycan spectrum (Fig. 1*C*) are the oligomannosidic glycans ranging from H_5_N_2_ to H_10_N_2_, and complex glycans expressing LDN-motifs observed at *m*/*z* 1582.7 [M-H]^−^ (F_1_H_3_N_4_) and *m*/*z* 1988.8 [M-H]^−^ (F_1_H_3_N_6_) for the mono- and di-antennary species, respectively. Glycans with one or two fucosylated LDN antenna were found to be expressed in relatively high amounts as well (F_2–3_H_3_N_6_ at *m*/*z* 2134.9 [M-H]^−^ and 2281.0 [M-H]^−^), and these observations are in accordance with our previous study of separate male and female worm *N*-glycans (11). Aided by the same study, the signal at *m*/*z* 2395.0 [M-H]^−^ was assigned to a glycan with a tandem-repeat of LDN (F_1_H_3_N_8_). Combinations of LDN-antennae and other antennae elements such as LN (*m*/*z* 1947.8 [M-H]^−^ (F_1_H_4_N_5_)), and truncated antennae (*m*/*z* 1785.7 [M-H]^−^ (F_1_H_3_N_5_)) were also present at considerable levels. Relatively low abundant signals were observed for LeX-containing glycans (F_2–3_H_5_N_4_ observed at *m*/*z* 2052.8 [M-H]^−^ and 2198.9 [M-H]^−^). The peaks at *m*/*z* 2271.9 [M-H]^−^ (F_1_H_6_N_5_) and 2109.9 [M-H]^−^ (F_1_H_5_N_5_) were derived from tri-antennary glycans, as confirmed by exoglycosidase digestion by JBG, leading to a loss of up to three Hex residues (supplemental Table S2). In contrast to the early life-stages, the complex type-glycans of adult worms differ dramatically with respect to core-modifications (core-Xyl absent in adult worms) and antennae-motifs (predominantly LDN in adult worms).

##### Changes in the Expression of N-glycan Motifs During the Development of Schistosome Cercariae to Adult Worms

*N*-glycan spectra (Supplemental Fig. S1*A*–S1*J*) recorded of the remaining stages of cercariae to adult worms development were interpreted on the basis of the compositions derived from the observed *m*/*z* values and exoglycosidase treatment of the life stages described above. The derived relative abundance of a number of characteristic structural motifs in the *N*-glycans over the course of worm development is depicted in Fig. 3*A*–3*D* (see also supplemental Table S4). Among four different general types of *N*-glycans (oligomannosidic, paucimannosidic, complex-type, and hybrid), pronounced shifts in the ratio between complex-type and oligomannosidic glycans are particularly clear (Fig. 3*A*). In cercariae, complex-type glycans and oligomannosidic glycans were detected in a ratio of 9:1 changing to a ratio of about 1:2 in 6-day-old-schistosomula, and then gradually to 1:1 in the adult worms. Pauci-mannosidic and hybrid-type glycans were expressed at relatively low overall levels of less than 10% throughout the whole range.

Schistosome *N*-glycans are carriers of core-modifications and terminal motifs that provoke humoral and cellular immune responses (40–44). The antigenic core-Xyl element is expressed on 70% of all cercarial *N*-glycans, but its expression rapidly decreases in the schistosomula stages, becoming undetectable in worms after 2 weeks of development (Fig. 3*B*). In contrast, the nonantigenic common core(α6)-Fuc modification that is mainly associated with complex-type glycans, was found to be abundant throughout schistosome development (Fig. 3*A*, 3*B*). Changes in *N*-glycan motifs during development of cercariae to adult worms are in particularly clear for LeX and LDN antigens. LeX was abundant in cercarial *N*-glycans (70% carry one or two LeX-antennae) (Fig. 3*C*) but levels of *N*-glycans expressing LeX rapidly decline during development to levels below 10%. In contrast, LDN-carrying structures showed the opposite pattern (Fig. 3*D*) with glycans containing one LDN-antenna increasing from around 2% in cercariae to 18% in the adult worms, and structures with two LDN-antennae from less than 1% to 12% of the total *N*-glycome. This means that among the complex-glycans in adult worms the LDN-antennae are dominant. Expression of fucosylated LDN-motifs was relatively constant within the PNGase F-released glycans over the different lifecycle-stages, with expression levels between 3 and 6% (supplemental Table S4). It is not possible to discriminate between the different structural isomers of these fucosylated LDN-elements on the basis of MALDI-TOF mass spectra alone. However, from the more detailed glycosidase treatment-assisted analysis of the cercarial, 3-day-old schistosomula and adult worms stages it can be concluded that *N*-glycans during worm development do not carry the Fucα3GalNAc-R (in F-LDN, F-LDN-F) or Fucα1–2Fucα1-R (DF) elements, suggesting that 3–6% of all *N*-glycan structures in the cercariae to adult worm life stages express LDN-F.

##### N-glycans of Mature Eggs

As a basis for the assignment of *N*-glycans during egg development we first analyzed, the mature egg. The spectrum of PGNase F-sensitive glycans of the mature eggs is shown in Fig. 2*B* and that of the PNGase A-sensitive glycans in Fig. 2*C*. Both strongly resembled previously published spectra (13, 20). Signals of oligomannosidic glycans (H_5–9_N_2_) are abundant among the PNGase F-sensitive glycans as is the tri-mannosyl glycan with both core(α6)-Fuc and core-Xyl (X_1_F_1_H_3_N_2_ at *m*/*z* 1308.4 [M-H]^−^). The species observed at *m*/*z* 2006.7 [M-H]^−^ (X_1_F_3_H_3_N_4_) corresponded to a glycan with core(α6)-Fuc and core-Xyl, and two HexNAcs and two Fuc-residues in the antennae. Based on the digestions with BKF, XMF, and NAG (supplemental Table S3) and data published by Khoo *et al.* (13), we concluded that this glycan carries an LDN antenna with a Fucα1–2Fucα1–3 motif linked to the GlcNAc residue (LDN-DF). This glycan element is completely resistant to fucosidase treatment with BKF and XMF, but NAG treatment leads to the loss of the unsubstituted GalNAc residue. Extensions of this glycan with additional fucoses were observed at *m*/*z* 2152.7 [M-H]^−^ (X_1_F_4_H_3_N_4_ (F-LDN-DF)), and *m*/*z* 2298.8 [M-H]^−^ (X_1_F_5_H_3_N_4_ (DF-LDN-DF)). Signals at *m*/*z* 2168.7 [M-H]^−^, *m*/*z* 2314.8 [M-H]^−^, and 2460.8 [M-H]^−^ (X_1_F_3–5_H_4_N_4_) correspond to glycans with one LeX-antenna, and one truncated antenna consisting of a GlcNAc residue substituted with a difucosyl or trifucosyl element (Fucα1–2Fucα1–3GlcNAcβ1–2R (DF-GlcNAc) or Fuc α1–2Fucαα1–3GlcNAcβ1–2R (TF-GlcNAc)). Other relatively abundant glycans carrying LN and LeX motifs were found at *m*/*z* 1906.6 [M-H]^−^, 2052.7 [M-H]^−^, 2198.7 [M-H]^−^ (F_1–3_H_5_N_4_), *m*/*z* 2330.8 [M-H]^−^ (X_1_F_3_H_5_N_4_), and at *m*/*z* 1947.6 [M-H]^−^, 2093.7 [M-H]^−^ (F_1–2_H_4_N_5_ together with LDN(-F) motifs). Finally, in the PNGase F released glycan pool we observed a characteristic signal at *m*/*z* 2526.8 (X_1_F_1_H_3_N_8_), corresponding to a glycan with core(α6)-Fuc, core Xyl and three LDN-antenna. Notably, the core(α3)-Fuc containing variant of the latter glycan is the main component of the PNGase A-specific *N*-glycan pool of mature eggs (Fig. 2*C*), giving rise to a signal *m*/*z* 2673.0 [M-H]^−^ (X_1_F_2_H_3_N_8_). This glycan composition matches with the major glycan present on the abundant egg glycoprotein kappa-5 (36). Fucosylated variants, carrying one or more LDN-F elements previously also detected on kappa-5, were detected in the overall glycan pool at *m*/*z* 2965.1 [M-H]^−^ (X_1_F_4_H_3_N_8_) and 2819.0 [M-H]^−^ (X_1_F_3_H_3_N_8_). Interestingly, in the same PNGase A glycan pool, the glycans with LeX antenna are mostly not xylosylated (mono-antennary F_3_H_4_N_3_ at *m*/*z* 1833.7 [M-H]^−^, di-antennary F_2–4_H_5_N_4_ at *m*/*z* 2052.8 [M-H]^−^, 2198.8 [M-H]^−^, and 2344.9 [M-H]^−^, or tri-antennary F_3_H_6_N_5_ at *m*/*z* 2563.9 [M-H]^−^). These LeX-bearing glycans have previously been found on the major excretory/secretory egg glycoproteins, IPSE/alpha-1 and omega-1 (37, 38). Other structures observed carry combinations of LDN, LDN-F, LN, or LeX-antennae. The structural characteristics of the PNGase A released glycans are strikingly different from the PNGase F sensitive pool, not only with respect to the core modifications but also in terms of antenna motifs.

##### N-glycans During Egg Development

It is likely that an important source of *N*-glycans in mature egg extracts is the miracidium, in contrast to immature eggs that contain the undeveloped embryo (45, 46). Indeed, the PNGase F released *N*-glycan profile of miracidia (Fig. 2*D*) resembles that of the mature egg (Fig. 2*B*). The PNGase A-spectrum of the miracidia (Fig. 2*E*); however, displays a striking difference with that of the egg (Fig. 2*C*). Signals derived from glycans terminating with a Gal residue (*e.g.* LN or LeX) visible in the *m*/*z* 1800–2700 range in the mature egg spectrum are completely absent from the miracidial spectrum. Core(α3)-Fuc modified glycans of the miracidium carry exclusively LDN or fucosylated LDN antenna, and the profile is dominated by the kappa-5 associated core-(α3/α6)difucosylated, xylosylated tri-antennary LDN-glycan (X_1_F_2_H_3_N_8_) at *m*/*z* 2673.4 [M-H]^−^. In contrast to the abundance of the core(α3)- and core(α3/α6)-fucosylated glycans found in mature eggs and miracidia, PNGase A-specifically released glycans were undetectable in immature eggs (data not shown). The PNGase F-released *N*-glycan profile of immature eggs (Fig. 2*A*) shows that in comparison to the mature eggs the expression levels of fucosylated antenna elements are low. Moreover, the major kappa-5 associated tri-antennary LDN glycan detected at *m*/*z* 2526.8 in Fig. 2*B* and *m*/*z* 2526.9 in Fig. 2*D* is completely absent in immature eggs, further indicating that this glycan and its variants originate from miracidia. The core Xyl-motif appears to be mainly associated with miracidia as well, because it is hardly detectable in immature eggs, and in mature eggs it is almost exclusively associated with the LDN containing *N*-glycans attributed to the miracidium. Furthermore, the observation that core(α3/α6)-difucosylated di-antennary glycans with LeX termini (*e.g. m*/*z* 2198.8 and 2344.9 in Fig. 2*B*), which have been found linked to the major egg E/S glycoproteins IPSE/α1 and omega-1 (37, 38), are present in mature eggs, but not in immature eggs or miracidia, indicates that these glycans are characteristic for glycoproteins expressed in egg compartments that develop during maturation.

##### O-glycans

**Fig. 4.**
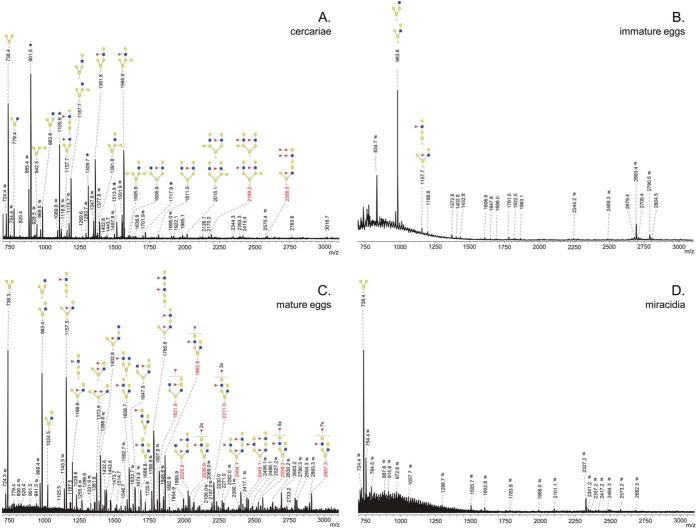
**MALDI-TOF-MS of the permethylated O-glycans obtained by reductive β-elimination from *S. mansoni* cercariae, *A*, immature eggs, *B*, mature eggs, *C*, and miracidia, *D*.** Glycans were analyzed as sodium-adducts in positive-ion reflectron mode. All signals are labeled with monoisotopic masses and structures based on the results of MALDI-TOF MS/MS fragmentations (masses indicated in black) supplemented with literature data (masses indicated in red). Red triangle, fucose; yellow circle, galactose; blue square, *N*-acetylglucosamine; yellow square, *N*-acetylgalactosamine. Signals corresponding to a hexose oligomer of unknown origin are marked with *. Nonglycan signals are marked with #.

*O*-glycans were obtained by reductive β-elimination of protein extracts from various schistosome life stages and permethylated prior to MS analysis. Glycan structures were assigned on the basis of compositions and MALDI-TOF-MS/MS fragmentations, taking into account previously published data when available. *O*-glycan spectra of the life stages studied are shown in Fig. 4*A*–4*D* and supplemental Fig. S2*A*–S2*H*. Examples of structural characterizations by MALDI-TOF-MS/MS are shown in supplemental Fig. S3.

##### O-glycan Expression During Development of Cercariae to Adult Worms

Cercariae express a range of *O*-glycans (Fig. 4*A*). Smaller glycans detectable at *m*/*z* 738.4 [M+Na]^+^ (H_2_N_1_) up to *m*/*z* 1565.9 [M+Na]^+^ (F_1_H_4_N_2_) are abundant, but signals over *m*/*z* 2000 indicating compositions including multiple Fuc-residues were also observed. As indicated in previous reports (12, 14, 17) and in line with MALDI-TOF-MS/MS fragmentation analyses (supplemental Fig. S3*A*–S3*C*), cercarial *O*-glycans were primarily based on the schistosome-specific Galβ1–3(Galβ1–6)GalNAc core (H_2_N_1_) (14) that was sometimes modified by one or two additional Gal residues (Fig. 4*A*). A minor *O*-glycan subset was identified based on the conventional type-2 core (*m*/*z* 779.4 [M+Na]^+^; H_1_N_2_). Similar to the *N*-glycans, the major terminal motif of cercarial *O*-glycans is the LeX-motif, which was present in glycans detected at *m*/*z* 1157.7 [M+Na]^+^ (F_1_H_2_N_2_), 1361.8 [M+Na]^+^ (F_1_H_3_N_2_), 1565.9 [M+Na]^+^ (F_1_H_4_N_2_), and in several, less abundant, more elaborate glycan-structures. The presence of the LeX motif was confirmed by the observation of a characteristic B-type fragment-ion at *m*/*z* 660 [M+Na]^+^ (F_1_H_1_N_1_) in the fragmentation analyses (supplemental Fig. S3*B*–S3*C*). Structures with nonfucosylated terminal elements (LN or GlcNAc) were also observed, *e.g.* at *m*/*z* 983.6 [M+Na]^+^ (H_2_N_2_), 1187.7 [M+Na]^+^ (H_3_N_2_) (fragmentation pattern shown in Supplemental Fig. S3*A*), and *m*/*z* 1391.8 [M+Na]^+^ (H_4_N_2_). Finally, the cercarial *O*-glycan spectrum contained a signal in the high mass-range at *m*/*z* 2589.5 [M+Na]^+^ corresponding to the highly fucosylated glycan with composition F_5_H_2_N_5_. This type of glycan, most likely based on a conventional type-2 core and having DF-LDN-DF, represents one of the previously described cercarial glycocalyx *O*-glycans (12). Based on MS signal intensity these unique multifucosylated glycans make up a relatively small fraction of the total *O*-glycome of cercariae. The overall cercarial *O*-glycan spectrum (Fig. 4*A*) is strikingly similar to that of the *O*-glycans derived from cercarial 0–3 h secretions (17), suggesting that the most abundant cercarial *O*-glycans are associated with glycoproteins excreted/secreted after transformation into schistosomula.

To examine changes in *O*-glycan expression in the first days after transformation we recorded mass spectra of *O*-glycans obtained from 3h, 24h, 48h, and 3-day-old *in vitro* cultured schistosomula (Supplemental Fig. S2*B*–S2*E*). Each of these spectra were highly similar to that of cercariae. However, overall intensity gradually diminished in the developing schistosomula to levels difficult to detect by MALDI-TOF MS in 3-day-old schistosomula (supplemental Fig. S2*B*–S2*E*), and to completely undetectable in 2 and 6-week-old worms (data not shown). The absence of *O*-glycans detectable by MS in mature schistosome stages has previously been described (11). It should be noted that adult worms produce the excretory Circulating Anodic Antigen (CAA) and Circulating Cathodic Antigen (CCA), which both carry long *O*-glycan chains with repeating di- or tri-saccharide motifs. These polysaccharides are difficult to detect by MS methods in their intact form, and their structures have previously been determined using NMR approaches (47, 48).

##### O-glycans of Eggs and Miracidia

Whereas the spectra of cercariae and schistosomula were dominated by signals below *m*/*z* 1600 [M+Na]^+^, the *O*-glycan mass spectrum of mature eggs in addition contains a large proportion of higher mass glycan signals (Fig. 4*C*), in line with previous studies (13, 17). In contrast to cercariae and schistosomula, most egg *O*-glycan structures are based on the mucin type-2 core (Galβ1–3(GlcNAcβ1–6)GalNAc), rather than the schistosomal Galβ1–3(Galβ1–6)GalNAc core, possibly mixed with type-1 core (Galβ1–3GalNAc)-based isomers for signals at *m*/*z* 1157.5 [M+Na]^+^ (F_1_H_2_N_2_), 1198.6 [M+Na]^+^ (F_1_H_1_N_3_) (fragmentation pattern shown in supplemental Fig. S3*D*), 1780.8 [M+Na]^+^ (F_2_H_3_N_3_), 2025.9 [M+Na]^+^ (F_2_H_3_N_4_), 2404.1 [M+Na]^+^ (F_3_H_4_N_4_), and 2445.1 [M+Na]^+^ (F_3_H_3_N_5_). Although the Galβ1–3(Galβ1–6)GalNAc core glycan was detected in eggs by a relatively intense signal at *m*/*z* 738.3 [M+Na]^+^ (H_2_N_1_), extensions thereof were only found at *m*/*z* 983.4 [M+Na]^+^ (H_2_N_2_) and 1606.7 [M+Na]^+^ (F_1_H_3_N_3_) (fragmentation pattern shown in supplemental Fig. S3*E*). Fragmentation-analysis confirmed that for most ions examined more than one possible isomer was underlying the registered *m*/*z* signal. For example, the fragmentation spectrum of *m*/*z* 1198.6 [M+Na]^+^ (F_1_H_1_N_3_) (supplemental Fig. S3D) showed characteristic fragments for both a type-1 core based structure (C-type fragment-ion at *m*/*z* 923.6 [M+Na]^+^ (F_1_H_1_N_2_)) and type-2 core based structures (Z-type fragment-ion at *m*/*z* 962.7 [M+Na]^+^ (F_1_N_3_)). Like cercariae and schistosomula, many *O*-glycans of eggs express LeX motifs, *e.g. m*/*z* 1157.5 [M+Na]^+^ (F_1_H_2_N_2_), but in the eggs, tandem repeats of LeX were uniquely observed e.g. at *m*/*z* 1780.8 [M+Na]^+^ (F_2_H_3_N_3_). Furthermore, in particular the higher mass egg *O*-glycans possess LDN-motifs, often as a (multi)fucosylated form, *e.g.* LDN-F at *m*/*z* 1198.6 [M+Na]^+^ (F_1_H_1_N_3_) (supplemental Fig. S3*D*), F-LDN-F or LDN-DF at *m*/*z* 2211.0 [M+Na]^+^ (F_4_H_1_N_5_) and DF-LDN-DF at *m*/*z* 2907.3 [M+Na]^+^ (F_8_H_1_N_6_). Finally, more unusual motifs were found at *m*/*z* 1647 [M+Na]^+^ (F_1_H_2_N_4_) and 1821.8 [M+Na]^+^ (F_2_H_2_N_4_), indicative of the presence of F-LDN with an additional hexose-residue, based on observations in literature (17) most likely galactose, and at *m*/*z* 1402.6 [M+Na]^+^ (F_1_H_2_N_3_) where a LeX-element was capped with an additional HexNAc, most likely GlcNAc (17). Thus, the egg-stage *O*-glycans are characterized by a multitude of different terminal motifs, of which LeX and (multi)fucosylated LDN appear to be the most abundant. The predominant core types were mucin type-2 and to a lesser extent type-1.

In sharp contrast to the mature egg, the *O*-glycan spectrum of miracidia contains only a single major glycan signal (Fig. 4*D*) at *m*/*z* 738.4 [M+Na]^+^ (H_2_N_1_). MS/MS-analysis indicated two hexose-residues attached to a reducing-end *N*-acetylhexosamine, most likely corresponding to the Galβ1–3(Galβ1–6)GalNAc *O*-glycan-core (supplemental Fig. S3*F*). This striking finding suggests that, in contrast to the *N*-glycans of miracidia, which are abundant and diverse, miradicia contribute with only a single type of *O*-glycan to the very complex *O*-glycome of eggs. Similarly contrasting, the *O*-glycan spectrum of immature eggs is dominated by the H_2_N_2_ glycan observed at *m*/*z* 983.6 [M+Na]^+^, and other *O*-glycans are hardly detectable (Fig. 4*B*). These observations suggest that the *O*-glycans of schistosome eggs are mainly associated with glycoproteins originating from egg compartments that form after maturation, but that are not part of the miracidium.

##### Lipid Glycans

**Fig. 5.**
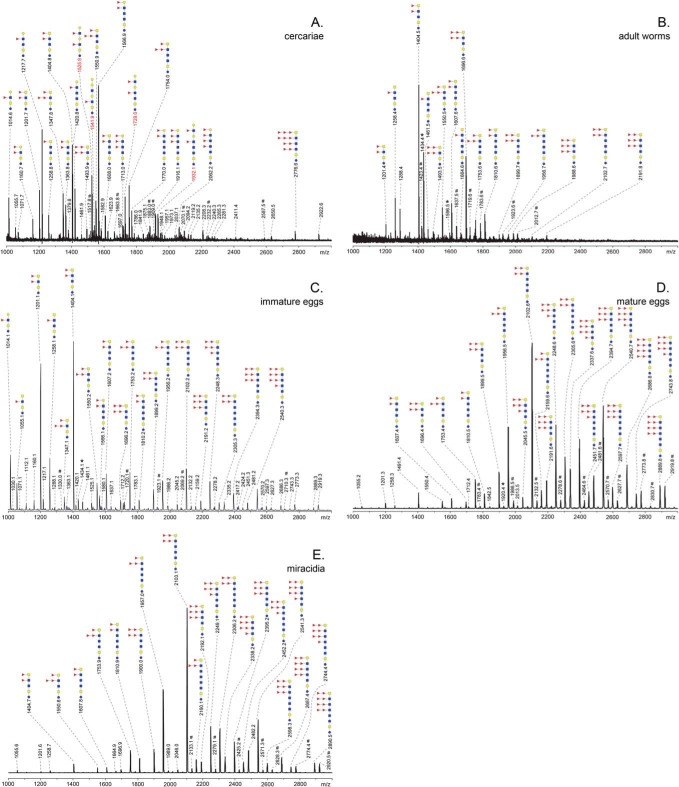
**MALDI-TOF-MS of the lipid-glycans of *S. mansoni* cercariae, *A*, adult worms, *B*, immature eggs, *C*, mature eggs, *D*, and miracidia, *E*, released by endoglycoceramidase digestion.** The 2-AA-labeled glycans were analyzed in negative-ion reflectron mode. All signals are labeled with monoisotopic masses. Compositions were deduced based on literature and MALDI-TOF MS/MS fragmentations (for masses indicated in red). In most cases, isomers caused by variable distribution of fucosyl residues along the backbone may occur, the most likely and/or most abundant isomer is indicated for each mass. Red triangle, Fucose; Yellow circle, Galactose; Blue circle, Glucose; Yellow square, *N*-acetylgalactosamine; Blue square, *N*-acetylglucosamine. Signals corresponding to hexose oligomer of unknown origin are marked with *. Nonglycan signals are marked with #.

Glycolipid glycans were released by endo-glycoceramidase and, after 2-AA-labeling, analyzed by MALDI-TOF-MS. Lipid-glycan spectra of the lifecycle stages studied are shown in Fig. 5*A*–5*E* and Supplemental Fig. S4*A*–S4*J*. Putative annotations of the mass spectra were performed in accordance with structural observations in earlier publications when available (18, 19, 21, 22), and when required MALDI-TOF-MS/MS fragmentations were recorded to further substantiate assignments (supplemental Fig. S5).

##### Lipid Glycosylation During the Development of Cercariae to Adult Worms

According to literature (18, 19, 21, 22, 49), all schistosome glycolipids are based on the so-called *schisto*-core GalNAcβ1–4Glc, where the glucose is attached to the ceramide-portion. In cercariae (Fig. 5*A*), one or more GlcNAc-moieties in a linear stretch with (oligo-)fucosyl side-chains could be found attached to this core, whereas the core itself was never found to be fucosylated (22). In full accordance with Wuhrer *et al.* (22), the most abundant lipid glycans in cercariae terminated with Hex or Fuc-Hex, thus giving rise to LeX- and pseudo-LeY-motifs (Fucα1–3Galβ1–4(Fucα1–3)GlcNAc). Major LeX containing glycans were found at *m*/*z* 1014.6 [M-H]^−^, 1217.7 [M-H]^−^, and 1420.8 [M-H]^−^ (F_1_H_2_N_2–4_). Glycans with pseudo-LeY were found at *m*/*z* 1160.7 [M-H]^−^, 1363.8 [M-H]^−^, 1566.9 [M-H]^−^, 1770.0 [M-H]^−^ (F_2_H_2_N_2–5_), and at *m*/*z* 1713.0 [M-H]^−^ (F_3_H_2_N_4_), 1916.1 [M-H]^−^, 2062.2 [M-H]^−^ (F_3–4_H_2_N_5_) with (multiple) additional fucosylated GlcNAc residues. Glycans terminating with two HexNAc-residues, most likely representing LDN-termini (22), were found at lower levels. Signals corresponding to glycans with F-LDN and F-LDN-F were seen at *m*/*z* 1201.7 [M-H]^−^ (F_2_H_1_N_3_), 1258.8 [M-H]^−^, 1404.8 [M-H]^−^ (F_1–2_H_1_N_4_), and 1608.0 [M-H]^−^ (F_2_H_1_N_5_). LDN epitopes presumably with (multiple) DF-motifs were found at *m*/*z* 1347.8 [M-H]^−^, 1493.9 [M-H]^−^ (F_3–4_H_1_N_3_) and 1550.9 [M-H]^−^, 1754 [M-H]^−^ (F_3_H_1_N_4–5_). Moreover, a heavily fucosylated glycan with trifucosyl (TF) side-chains and DF-motifs was found at *m*/*z* 2776.6 [M-H]^−^ (F_10_H_1_N_5_). Finally, we observed glycans with tandem-repeats of LeX or a unique Hex-Hex terminus, as confirmed by fragmentation analysis (supplemental Fig. S5). These structures at *m*/*z* 1525.9 [M-H]^−^, 1729.0 [M-H]^−^, 1932.1 [M-H]^−^ (F_2_H_3_N_3–5_), and 1541.9 [M-H]^−^ (F_1_H_4_N_3_) have not been described before among cercarial lipid glycans.

Surprisingly, the overall glycolipid glycan profile did not significantly change after transformation of cercariae followed by 9 days culture (supplemental Fig. S4*A*–S4*J*). Unlike the N-glycan and *O*-glycan profiles, which rapidly change after transformation, the glycolipids remain constant during the first days of schistosomula development.

The lipid glycan spectrum of the adult worms is however different from that of cercariae (Fig. 5*A*, 5*B*). The glycans of the adult worms lack LeX and pseudo-LeY termini but instead seem to exclusively terminate with fucosylated LDN. Frank *et al.* (18) showed that these structures could be divided in a group of glycans with a somewhat lower degree of fucosylation with fucoses solely present on the terminal LDN-motif, and a group with higher fucosylated species with additional fucosylation of the GlcNAc-backbone, and we annotated our glycolipid glycan mass spectra accordingly. Fully in accordance with Frank *et al.* (18) glycans expressing these fucosylated LDN-motifs generally contained between three and six HexNAc-moieties, with the species containing four HexNAcs being the most prominent *e.g. m*/*z* 1404.5 [M-H]^−^ (F_2_H_1_N_4_) and 1696.6 [M-H]^−^ (F_4_H_1_N_4_) carrying F-LDN-F and DF-LDN-DF as terminal-motifs, respectively.

##### Surface Expression of Glycan Epitopes

**Fig. 6.**
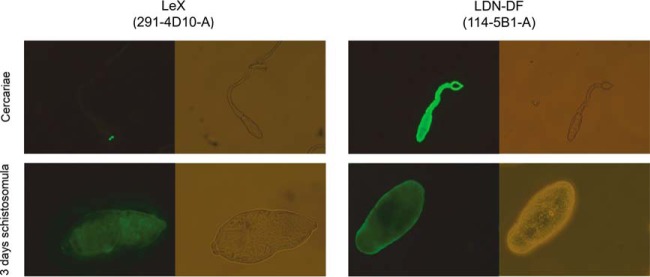
**Surface expression of LeX- and LDN-DF-motifs in cercariae and 3 days schistosomula.** Cercariae and 3 days schistosomula were incubated with the monoclonal antibodies 291–4D10-A (antiLeX) and 114–5B1-A (antiLDN-DF) and observed by fluorescence microscopy. For each fluorescence image the corresponding transmitted light (Phase-Contrast) image is shown. Images for cercariae were taken with a 10× magnification, images for 3 days schistosomula were taken at 40× magnification.

In view of the differential expression of various glycan antigens during early worm development and the potential consequences of their exposure to the host we were triggered to study the surface expression of a number of terminal glycan-motifs in cercariae and 3-day schistosomula by detection with previously described monoclonal antibodies (34). In line with previous studies (26, 34), LeX-motifs were detected at the oral sucker of cercariae, but 3 days after transformation we could detect LeX over the whole surface of the parasite (Fig. 6). This increase of surface expressed LeX-motifs in schistosomula is in sharp contrast with the general trend that the expression of LeX-motifs by *N*- and *O*-glycans declines during development (Fig. 3*A*, supplemental Fig. S5*A*–S5*H*). These data suggest that, although LeX expression is high on cercarial glycoproteins, this motif is not exposed at the cercarial surface. Possibly, LeX surface expression after shedding of the glycocalyx and secretion of cercarial ES is caused by exposure of the LeX containing glycolipids that remain abundant after transformation, rather than expression on *N*- or *O*-glycan, which decreases instead. In contrast, for the LDN-DF-motif we observed a sustained surface expression in both cercariae (34) and 3 days schistosomula (Fig. 6). LDN-DF motifs were not detected on *N*-glycans in cercariae and schistosomula, but they are present in the *O*-glycan and lipid glycan pools of these life stages.

##### Lipid-glycans of Eggs and Miracidia

Mature egg lipid glycans were very similar to those of the adult worms with respect to the terminal fucosylated-LDN motifs detected (Fig. 5*D*). Egg lipid glycans had longer HexNAc-backbones (5–7 HexNAcs) compared with the adult worm lipid glycans (4–5 HexNAcs), and showed a much higher degree of fucosylation, in line with previously published egg lipid glycan spectra (19, 21). Strong signals of multiple glycans comprising six to eight Fuc-moieties where observed in the mass range of *m*/*z* 2191–2889 [M-H]^−^. The spectrum of the miracidia lipid glycans (Fig. 5*E*) was almost identical to the egg lipid glycans. No unique signals or motifs were observed compared with mature eggs, only slight quantitative changes. On the other hand, the spectrum of lipid glycans derived from immature eggs (Fig. 5*C*) is shifted toward the lower mass glycans (*m*/*z* 1000–2600 [M-H]^−^). Therefore, miracidia appear to be a major source of the large highly fucosylated lipid glycans in the mature eggs, but a subset of short chain glycans with similar glycan motifs also abundantly expressed by adult worms is present in the immature eggs.

## DISCUSSION

Genomic, transcriptomic, and proteomic studies of schistosomes have revealed a wealth of knowledge with respect to the developmental expression of genes and proteins during the schistosome life cycle (3, 50–58). In the current study, we performed a mass spectrometry-based overall glycan analysis covering all developmental stages of *S. mansoni* that interact with the human host, thereby filling the significant gaps that remained in the glycomic map of the schistosome life cycle. We identified a number of striking shifts and switches in the expression of protein- and/or lipid-linked immunogenic glycan motifs.

Schistosome glycans are involved in the stimulation or modulation of innate immune responses of the host, and they provoke adaptive immune responses, in particular as antibody target (59–61). During penetration of the host's skin and subsequent transformation into schistosomula, schistosome cercariae secrete a set of highly glycosylated proteins (0–3 h ES) that facilitate entry through the skin and modulate the immune response (62–64). We found that the overall *N*- and *O*-glycan profiles of cercariae (Figs. 1*A*, 4*A*) are strikingly similar to those of 0–3 h ES (17). Together with the observed gradual decline of the total *O*-glycan content after transformation until *O*-glycans become undetectable in 3-day-old schistosomula, this suggests that the *O*-glycans of the larval schistosome stages are mainly associated with its secretions. Similarly, the core xylosylated *N*-glycans with LeX antennae that are abundant in 0–3 h ES (17) disappeared within 3 days from the changing overall schistosomula *N*-glycan profile (Fig. 1*A*, 1*B* and supplemental Fig. S1*A*–S1*D*). The high-mass multifucosylated *O*-glycans that constitute the cercarial glycocalyx (12) comprise another, however, relatively minor subset of the total *O*-glycan-repertoire that disappears from the spectrum within days (Fig. 4*A* and supplemental Fig. S2*A*–S2*E*). This subset presumably becomes undetectable in schistosomula because the cercarial glycocalyx is rapidly shed upon transformation (57, 63, 65, 66). In contrast to the *N*-glycans, the lipid-derived glycan repertoire remained surprisingly stable from cercariae up to at least 9-day-old juvenile worms (Fig. 5*A* and supplemental Fig. S4*A*–S4*F*). A subset of these glycolipids carry LeX motifs, just as the cercarial *N*- and *O*-glycans, but equally abundant cercariae/schistosomula glycolipid-associated motifs are pseudo LeY and variations of multifucosylated LDN and GlcNAc stretches. Using LeX-specific mAbs it has been shown that, in cercariae, LeX is only present at the oral sucker, whereas in 3-day-old schistosomula the whole surface displayed patches of LeX (Fig. 6) (25, 26, 34). In view of the above data, the latter observation may be because of the surface exposure of lipid-linked rather than protein-linked LeX in the developing schistosomulum tegument. Multifucosylated LDN elements such as the LDN-DF motif bound by mAb 114–5B1 cover the complete surface of cercariae (Fig. 6) (34), presumably as part of glycocalyx-associated *O*-glycans. Surface exposure of LDN-DF at the schistosomula surface remains however after *O*-glycans have disappeared (Fig. 6), most likely because of exposure of the glycolipid-associated multifucosylated LDN motifs. Although protein-associated fucosylated glycan motifs, in particular different structural variations of LeX, have been shown to be directly or indirectly involved in the induction of immunomodulatory mechanisms by 0–3 h ES as well as by schistosome egg antigens (5, 64, 67), it is not known if glycolipids from cercariae and schistosomula that carry identical glycan motifs could also induce such effects. Innate immune responses of human PBMCs were triggered however by egg glycolipid preparations that carry LDN-DF (6) and worm-derived fucosylated glycolipids stimulate an inflammatory DC phenotype (68), suggesting that also larval glycolipids may trigger immunomodulatory effects if they would be appropriately exposed to the host immune system.

Regardless of possible immunomodulatory properties it is clear that many glycan motifs, either shared between lipids and proteins (*e.g.* LeX, multifucosylated LDN motifs), or unique (*e.g.* core-Xyl on *N*-glycans, *O*-glycan core motifs, pseudo LeY on glycolipids) form potential targets of the antibody response in the schistosome-infected host (59). Using ELISA, surface plasmon resonance, and/or glycan array approaches, antibody targets in human and animal schistosome infection sera obtained from different endemic and experimental settings and cohorts include in any case LDN, LeX, F-LDN, LDN-F, multifucosylated variants of LDN and HexNAc, including DF-elements, core-Xyl, and core-(α3)Fuc (41–44, 69–74). It is also likely that other so far unexplored motifs such as the additional β1–6Gal residues linked to cercarial *O*-glycans (Fig. 4*A*) and the digalactosylated lipid glycans (Fig. 5*A*) are antigenic. Novel glycan array approaches using complete sets of native, isolated glycans (70) will help uncover these so far ignored glycan antigens.

Interestingly, the expression of protein-associated immunogenic glycan motifs appears to decrease during schistosomula to worm development (Figs. 1, 3, and supplemental Figs. S1*A*–S1*J*, S2*A*–S2*E*). It is not known to which effect, but in particular the worm *N*-glycans are dominated by oligomannose and LDN glycans, with relatively little fucose content. Perhaps exposure of glycans to which no particular immunogenic properties have been attributed is an adaptation for survival in the blood stream of the host, whereas the cercariae and eggs, which have to pass the tissue to ensure development and completion of the schistosome life cycle, favor expression of the generally more immunogenic fucosylated glycans. It has been shown that the LDN-motif, a disaccharide element that occurs widely among helminths (60) can be bound by the C-type lectin receptor MGL (75, 76), and by galectin-3 (77) but it is unclear if these interactions lead to functional consequences in schistosomiasis. LDN-coated Sepharose beads as model for schistosome eggs do give rise to the formation of hepatic granulomas in the mouse (8), but inflammation around schistosome worms that expose LDN in the bloodstream has not been reported. On the other hand, an LDN-binding mAb in the presence of complement was capable of killing schistosomula *in vitro* (26) suggesting that LDN may be the target of an effective immune response. Antibody responses to LDN in schistosome-infected humans and animals have indeed been observed, but in general these seem to be predominantly of the IgM type and rather weak, in particular compared with antibodies against highly fucosylated glycan motifs (26, 70, 71). Low antigenicity is not a general feature of worm-associated glycan motifs however. When schistosomula develop a gut they start to excrete a polymeric form of LeX as part of circulating cathodic antigen (CCA) (47), and worm glycolipids contain antigenic fucosylated LDN elements similar to cercariae and eggs (18) (Fig. 5*B*). In developing and adult worms, F-LDN-, F-LDN-F-, and DF-containing *N*- or *O*-glycans were not observed by MS (Supplemental Figs. S1*B*–S1*J* and S2*A*–S2*E*). In line with this, mAbs 114–5B1 and 114–4D12 that bind to DF-containing motifs recognize only few worm proteins in a Western blot, and the F-LDN-F motif has been shown to be exclusively associated with glycolipids in parenchymal ducts of so far unknown function in an IFA study of adult worm sections (34, 78).

The current and previous studies reveal a strong overlap between glycan structures of schistosome cercariae and eggs (Figs. 1*A*, 2, 4, 5*A*, 5*C*, 5*D*, 5*E*) (17, 20, 26, 78). Shared glycan motifs are the cause of abundant antigen cross-reactivity between cercariae and eggs (10, 17, 79–81). Some glycan motifs are, however, unique to the cercariae and early schistosomula, such as the pseudo LeY motif and the galactosylated *O*-glycan core. It remains to be investigated if these unique cercarial glycans harbor specific immunogenic properties, but they may be interesting as a target of specific anticercarial antibodies in the development of a diagnostic assay or for immunization against schistosome infection. Surprisingly, although LeX motifs in various monomeric and multimeric contexts are abundant in *N*- and *O*-glycans during cercariae to worm development as well as egg development (with the exception of miracidia), LeX on glycolipids only appears in cercariae and early schistosomula. The biological relevance of the additional expression of LeX on cercarial glycolipids, while being absent from worm and egg glycolipids is not known. Possibly, the absence of LeX on egg glycolipids is the result of an overall absence of a β4-galactosyltransferase (β4-GalT) activity in the egg compartments in which the glycolipid synthesis takes place. The β4-GalT activity relative to β4-GalNAcT seems to dramatically decrease in miracidia. In miracidia we found exclusively LDN-based *N*- and *O*-glycan antennae and glycolipids, whereas nonmiracidial egg *N*- and *O*-glycans also contain the LN-based LeX motif (Figs. 2, 4, and 5).

The immature *S. mansoni* egg does not yet contain a developed miracidium, nor an active subshell Von Lichtenbergs envelope or Reynolds layer that secrete ES glycoproteins (45). Immature eggs display an *N*-glycan profile dominated by nonspecific, common oligomannose glycans and LN-motifs (Fig. 2*A*), a lipid glycan profile that is dominated by the F-LDN-F motif that is expressed throughout development (Fig. 5, Supplemental Fig. S4*A*–S4*J*), and simple short *O*-glycans (Fig. 4*B*). The mature egg however, which does include the miracidium as well as the subshell layers, displays highly complex fucosylated *N*-glycans, including LeX, LDN substituted with F and DF, core xylose and core-(α3)-Fuc (Fig. 2*B*, 2*C*). This core-(α3)-Fuc modification, which occurs mainly in combination with the core-(α6)-Fuc, forming a di-fucosylated Asn-linked GlcNAc residue, was not detectable by MS in the other life stages studied except miracidia and mature eggs. Also observed in mature eggs were complex and large *O*-glycans with multiple LeX motifs and highly fucosylated LDN elements (Fig. 4*C*), the latter group previously identified as the target of a diagnostic mAb 114–4D12 used to detect SEA (82). Comparison of the glycome of hatched miracidia with that of the mature egg suggests that miracidia are the main source of the *N*-glycans observed in the mature egg preparation. The PNGase F profiles of mature eggs and miracidia are almost identical (Fig. 2*B*, 2*D*). The PNGase A profiles are identical with respect to all core-(α3)-Fuc, core-Xyl, LDN-containing glycans, however a subset of nonxylosylated core(α3/α6)-fucosylated LeX-containing glycans are unique to the mature egg and not present in the miracidia (Fig. 2*C*, 2*E*). The latter glycans are identical to those present on the major ES glycoproteins IPSE/α1 and omega-1 (37, 38), and they have been identified among glycans released from ES (17). The sites of production of IPSE/α1 are the Von Lichtenbergs envelope and Reynolds Layer (83), and for omega-1 the Von Lichtenbergs envelope (84). We hypothesize that this subset of LeX-containing glycans is uniquely associated with the egg ES glycoproteins that are produced in these envelope cells associated with the mature egg, whereas all other *N*-glycans, including the dominant core-xylosylated, core-(α3/α6) difucosylated tri-antennary LDN glycan (Fig. 2*E*) that was found on kappa-5 (36) represent miracidum-derived glycoproteins. Kappa-5 appears to be produced and secreted by the miracidium inside the egg as it has been found in the subshell area, however, not in the cellular layers (85). Surprisingly, in the hatched miracidium we observed only a single short *O*-glycan (Fig. 4*D*), and all complex and fucosylated *O*-glycans observed in the mature egg (Fig. 4*C*) seem also to be produced by the mature egg associated subshell layers. Similar *O*-glycans were indeed detected in egg ES (17), and DF-LDN-DF-containing *O*-glycans were detected by IFA surrounding the egg in frozen liver sections (82), indicating these fucosylated *O*-glycans are associated with one or more abundant components from egg ES. In previous studies, *O*-glycans were not found on the most abundant ES glycoproteins IPSE/α1 and omega-1 (37, 38), and it remains to be determined which ES glycoproteins do carry these *O*-glycans. Like in cercariae and adult worms (see above), *O*-glycosylation and LeX-expression in eggs seem mainly associated with secretions. Miracidia glycans are almost completely devoid of LeX- or LN-motifs (Fig. 2*D*, 2*E*). In particular, the core (α3/α6) di-fucosylated *N*-glycan pool characteristic for miracidia (Fig. 2*E*) is exclusively based on LDN antennae. It has previously been shown that LeX apparently does not play a role in snail infection as this motif is not expressed in the intra-molluscan stages of *S. mansoni*. In contrast, in these stages LDN-based motifs are abundant, both in the parasite and the intermediate host (86–90). This cross-reactivity might indicate that the developing miracidium already adapts a snail-compatible glycosylation pattern, similar to cercariae that express high levels of LeX motifs that may be important for successful invasion of the human host.

An important question open for further research is how the molecular context of a glycan motif plays a role in its immunogenic properties. These properties may be dependent on the presence on either a protein or a lipid carrier, on the structure of the underlying glycan (combination with other motifs, number of antennae, chain length) and on its specific exposure on secretions or on the parasite surface during different stages of the infection. In particular, the LeX motif has been extensively studied in different immunological and structural contexts. LeX as part of multivalent synthetic glycoconjugates has been shown to be capable of inducing and modulating DC-SIGN and TLR4-mediated Th2 responses (91, 92) but in the context of IPSE/α1 and omega-1, only LeX in omega-1 was involved in the induction of Th2 responses, in this case by mediating uptake of omega-1 by DC via MR (5). SEA does however induce Th2-associated effects similar to polyvalent LeX conjugates in certain biological systems (67, 93) and the LeX-containing *O*-glycans that are abundantly expressed in ES products may play a role in this. Both cercarial and egg ES are rich in complex multifucosylated LDN-containing glycans as well, but this specific group of glycans has not been explored so far in biological/immunological studies. Although lectin receptors for LeX, LDN and LDN-F have been identified (75, 77, 94), no specific receptors for other abundant motifs such as F-LDN-F and the DF-containing glycans have been described. It will be interesting to test if the latter group of fucosylated glycan motifs harbors similar or perhaps contrasting immunomodulatory properties to LeX.

Regardless of their potential immunomodulatory properties, most glycan motifs expressed on schistosome glycoconjugates give rise to antibodies in the infected human or animal host. Generally higher antibody titers are observed against schistosome/pathogen specific glycans motifs such as DF, compared with motifs that are shared with the host such as LeX and LDN-F (26, 70, 71), but in addition the antiglycan responses are highly related to infection parameters (59, 70). Although it would be out of the scope of this paper to address the relevance of antiglycan antibodies in schistosomiasis immunity in detail, our data supports further research into the question if antiglycan antibodies might serve as a decoy for the immune system, directing antibodies away from the vulnerable invading schistosomulum (17), or that antiglycan antibodies may be involved in protective immune mechanisms. We believe it is relevant that some antibody targets are shared between different life stages, in particular cercariae and eggs, whereas others appear to be characteristic or unique for a specific developmental stage. More research into antibody responses in defined human and animal cohorts toward a large number of glycan motifs and glycans described in this study will be needed to uncover the relation of each of the antiglycan antibodies to schistosome infection and immunity.

In conclusion, we presented the first comprehensive comparative analysis of schistosome glycans expressed during interaction of the parasite with the definitive host, and generated new insights into the biology and immunology associated with antigenic schistosome glycan motifs.

## Supplementary Material

Supplemental Data

## Footnotes

^1^ The abbreviations used are:

DC: dendritic cell
MR: mannose receptor
MGL: macrophage galactose-type lectin
DC-SIGN: dendritic cell-specific ICAM-3-grabbing nonintegrin
Th2: T helper 2
F: Fuc or fucose
DF: difucosyl or Fucα1–2Fucα1–3
TF: trifucosyl or Fucα1–2Fucα1–2Fucα1–3
H: Hex or hexose
N: HexNAc or *N*-acetylhexosamine
X: Xyl or xylose
Gal: Galactose
GalNAc: *N*-acetylgalactosamine
GlcNAc: *N*-acetylglucosamine
LDN: LacDiNAc or GalNAcβ1–4GlcNAc
LN: LacNAc or Galβ1–4GlcNAc
LeX: Lewis X or Galβ1–4(Fucα1–3)GlcNAc
LDN-F: GalNAcβ1–4(Fucα1–3)GlcNAc
F-LDN-F: Fucα1–3GalNAcβ1–4(Fucα1–3)GlcNAc
F-LDN: Fucα1–3GalNAcβ1–4GlcNAc
Pseudo-LeY: pseudo Lewis Y or Fucα1–3Galβ1–4(Fucα1–3)GlcNAc)
CAA: circulating anodic antigen
CCA: circulating cathodic antigen
SEA: soluble egg antigens
IPSE: interleukin-4-inducing principle from schistosome eggs
PNGase F: *N*-glycosidase F
PNGase A: *N*-glycosidase A
rEGCase II: recombinant endoglycoceramidase II
NAG: β-*N*-acetylglucosaminidase from jack bean
BKF: α-L-fucosidase from bovine kidney
XMF: α(1–3,4)-fucosidase from *Xanthomonas manihotis*
JBG: β(1–4,6)-galactosidase from jack bean
2-AA: 2-aminobenzoic acid
DHB: 2,5-dihydroxybenzoic acid
NMR: nuclear magnetic resonance
RP: reversed phase
MeOH: methanol
ES: excretory secretory molecules.
