# Supplementary material for: Glycomic Analysis of Life Stages of the Human Parasite Schistosoma mansoni Reveals Developmental Expression Profiles of Functional and Antigenic Glycan Motifs
Source: Mol Cell Proteomics. 2015 Apr 16;14(7):1750–69. doi: 10.1074/mcp.M115.048280 (PMC4587318; doi:10.1074/mcp.M115.048280)
Supplement: Supplemental Data [file supp_14_7_1750__index.html]

Glycomic analysis of life stages of the human parasite Schistosoma mansoni reveals developmental expression profiles of functional and antigenic glycan motifs. — Glycomic Analysis of Life Stages of the Human Parasite Schistosoma mansoni Reveals Developmental Expression Profiles of Functional and Antigenic Glycan Motifs — Developmental Glycosylation of S. mansoni — Supplemental Data 

# Glycomic Analysis of Life Stages of the Human Parasite *Schistosoma mansoni* Reveals Developmental Expression Profiles of Functional and Antigenic Glycan Motifs

## Supplemental Data

**Files in this Data Supplement:**

- Legends to Supplemental Figures - Legends to Supplemental Figures
- Supplemental Figure 1 - MALDI-TOF-MS of the PNGase F-sensitive N-glycans of *S. mansoni* cercariae (A), 3hr schistosomula (B), 24hr schistosomula (C), 3 days schistosomula (D), 6 days schistosomula (E), 2 weeks worms (F), 3 weeks worms (G), 4 weeks worms (H), 5 weeks worms (I), 6 weeks worms (J)
- Supplemental Figure 2 - MALDI-TOF-MS of the permethylated O-glycans of *S. mansoni* cercariae (A), 3hr schistosomula (B), 24hr schistosomula (C), 48hr schistosomula (D), 3 days schistosomula (E), immature eggs (F), mature eggs (G) and miracidia (H) released by reductive &#x26;#946;-elimination
- Supplemental Figure 3 - MALDI-TOF-MS/MS analysis of permethylated O-glycans
- Supplemental Figure 4 - MALDI-TOF-MS of the lipid-glycans of *S. mansoni* cercariae (A), 3hr schistosomula (B), 24hr schistosomula (C), 48hr schistosomula (D), 3 days schistosomula (E), 9 days schistosomula (F), adult worms (G), immature eggs (H), mature eggs (I) and miracidia (J) released by endoglycoceramidase digestion
- Supplemental Figure 5 - MALDI-TOF-MS/MS analysis of 2-aminobenzoic acid-labeled cercariae lipid-glycans
- Supplemental Tables 1-3 - Overview of exoglycosidase digestions
- Supplemental Table 4 - Relative abundance and structural characteristics of N-glycans during worm development
- Supplemental Table 5 - Terminal glycan motifs in *S. mansoni* glycoconjugates
