# Supplementary material for: Glycomic Analysis of Life Stages of the Human Parasite Schistosoma mansoni Reveals Developmental Expression Profiles of Functional and Antigenic Glycan Motifs
Source: Mol Cell Proteomics. 2015 Apr 16;14(7):1750–69. doi: 10.1074/mcp.M115.048280 (PMC4587318; doi:10.1074/mcp.M115.048280)
Supplement: Supplemental Data [file supp_M115.048280_mcp.M115.048280-1.pdf]

Legends to Supplemental Figures

Supplemental Figure 1. MALDI-TOF-MS of the PNGase F-sensitive N-glycans of *S. mansoni* cercariae (A), 3hr schistosomula (B), 24hr schistosomula (C), 3 days schistosomula (D), 6 days schistosomula (E), 2 weeks worms (F), 3 weeks worms (G), 4 weeks worms (H), 5 weeks worms (I), 6 weeks worms (J). Released glycans were labeled with 2-aminobenzoic acid and analyzed in negative-ion reflectron mode. All signals are labeled with monoisotopic masses and structures deduced from these masses on basis of digestions with exoglycosidases and literature data. Red triangle, fucose; yellow circle, galactose; blue square, N-acetylglucosamine; green circle, mannose; yellow square, N-acetylgalactosamine; white star, xylose. M4–M9, oligomannosidic N-glycans with 4–9 mannose residues. M10, oligomannosidic N-glycan with 9 mannose and 1 glucose residue. Signals corresponding to a hexose ladder are marked with *.

Supplemental Figure 2. MALDI-TOF-MS of the permethylated O-glycans of *S. mansoni* cercariae (A), 3hr schistosomula (B), 24hr schistosomula (C), 48hr schistosomula (D), 3 days schistosomula (E), immature eggs (F), mature eggs (G) and miracidia (H) released by reductive β-elimination. Glycans were analyzed as sodium-adducts in positive-ion reflectron mode. All signals are labeled with monoisotopic masses and structures based on the results of MALDI-TOF MS/MS fragmentations (masses indicated in black) supplemented with literature data (masses indicated in red). Red triangle, fucose; yellow circle, galactose; blue square, N-acetylglucosamine; yellow square, N-acetylgalactosamine. Signals corresponding to a hexose ladder are marked with *.

Supplemental Figure 3. MALDI-TOF-MS/MS analysis of permethylated O-glycan signals at m/z 1187 $[M+Na^+]$ H_3N_2 (A), 1361 $[M+Na^+]$ $F_1H_3N_2$ (B) and 1565 $[M+Na^+]$ $F_1H_4N_2$ (C) found in cercariae, 1198 $[M+Na^+]$ $F_1H_1N_3$ (D) and 1606 $[M+Na^+]$ $F_1H_3N_3$ (E) found in eggs and 738[$M+Na^+$] H_2N_1 (F) found in miracidia. Fragments were registered as sodium-adducts in positive-ion reflectron mode. Relevant signals are labeled with monoisotopic masses and structures. Red triangle, fucose; yellow circle, galactose; blue square, N-acetylglucosamine; yellow square, N-acetylgalactosamine.

Supplemental Figure 4. MALDI-TOF-MS of the lipid-glycans of *S. mansoni* cercariae (A), 3hr schistosomula (B), 24hr schistosomula (C), 48hr schistosomula (D), 3 days schistosomula (E), 9 days schistosomula (F), adult worms (G), immature eggs (H), mature eggs (I) and miracidia (J) released by endoglycoceramidase digestion. Glycans were labeled with 2-aminobenzoic acid and analyzed in the negative-ion reflectron mode. All signals are labeled with monoisotopic masse. Compositions were deduced based on literature and MALDI-TOF MS/MS fragmentations (for masses indicated in red). In most cases isomers due to variable distribution of fucosyl residues along the backbone may occur, the most likely and/or most abundant isomer is indicated for each mass. F, fucose; H, hexose; N, N-acetylhexosamine. Signals corresponding to a hexose ladder are marked with *.

Supplemental Figure 5. MALDI-TOF-MS/MS analysis of 2-aminobenzoic acid-labeled cercariae lipid-glycans observed at m/z 1525 $[M-H^+]$ $F_2H_3N_3$ (A), 1541 $[M-H^+]$ $F_1H_4N_3$ (B) and 1728 $[M-H^+]$ $F_2H_3N_4$ (C). Fragments were obtained in negative-ion reflectron mode. Structurally

relevant signals are labeled with monoisotopic masses and structures. Red triangle, fucose; blue circle, glucose; yellow circle, galactose; blue square, N-acetylglucosamine; yellow square, N-acetylgalactosamine.
