# Supplementary material for: Glycomic Analysis of Life Stages of the Human Parasite Schistosoma mansoni Reveals Developmental Expression Profiles of Functional and Antigenic Glycan Motifs
Source: Mol Cell Proteomics. 2015 Apr 16;14(7):1750–69. doi: 10.1074/mcp.M115.048280 (PMC4587318; doi:10.1074/mcp.M115.048280)

# Suppl. Fig. 1A
PNGase F-sensitive glycans of cercariae

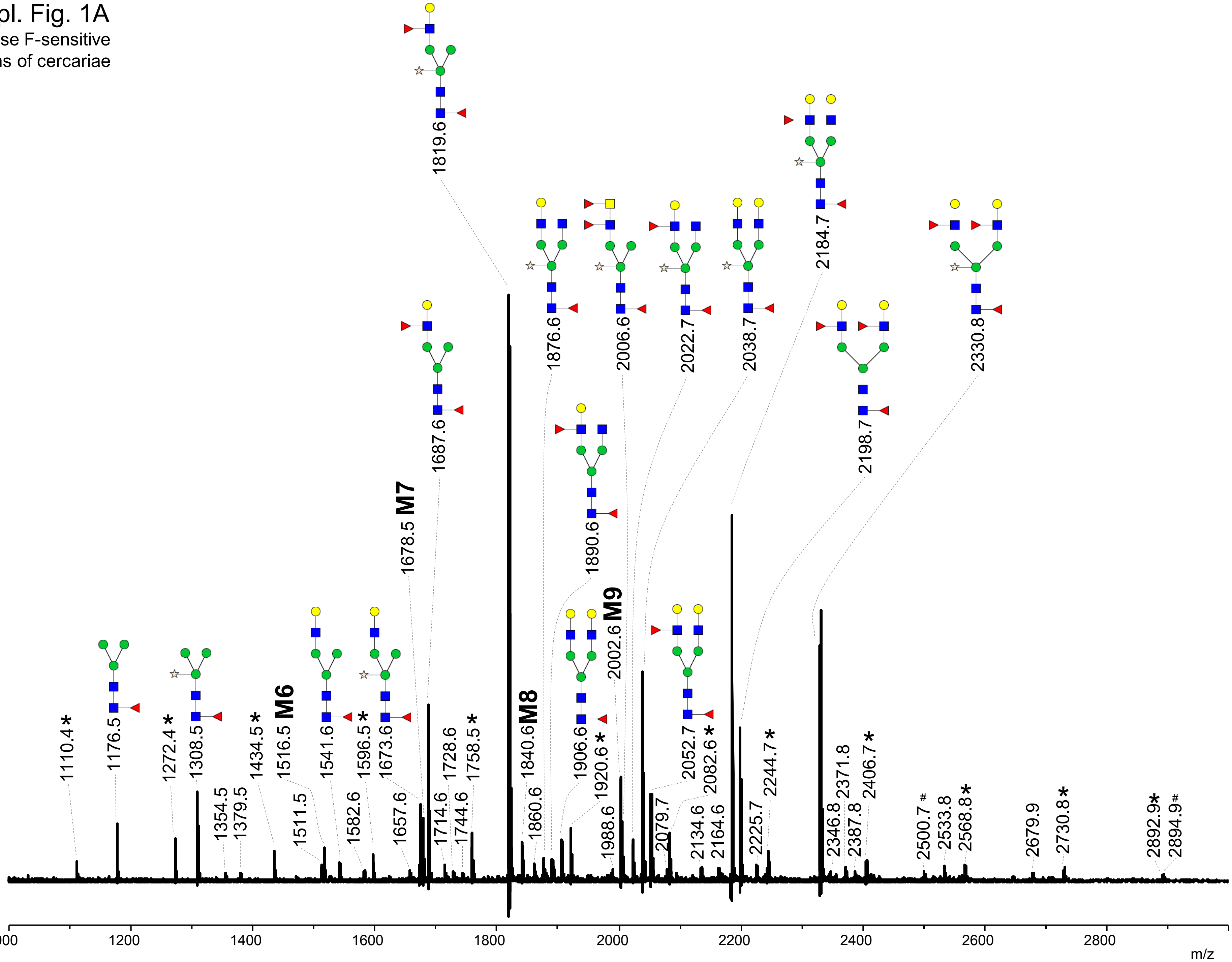

Suppl. Fig. 1B
PNGase F-sensitive
glycans of 3 hr schistosomula

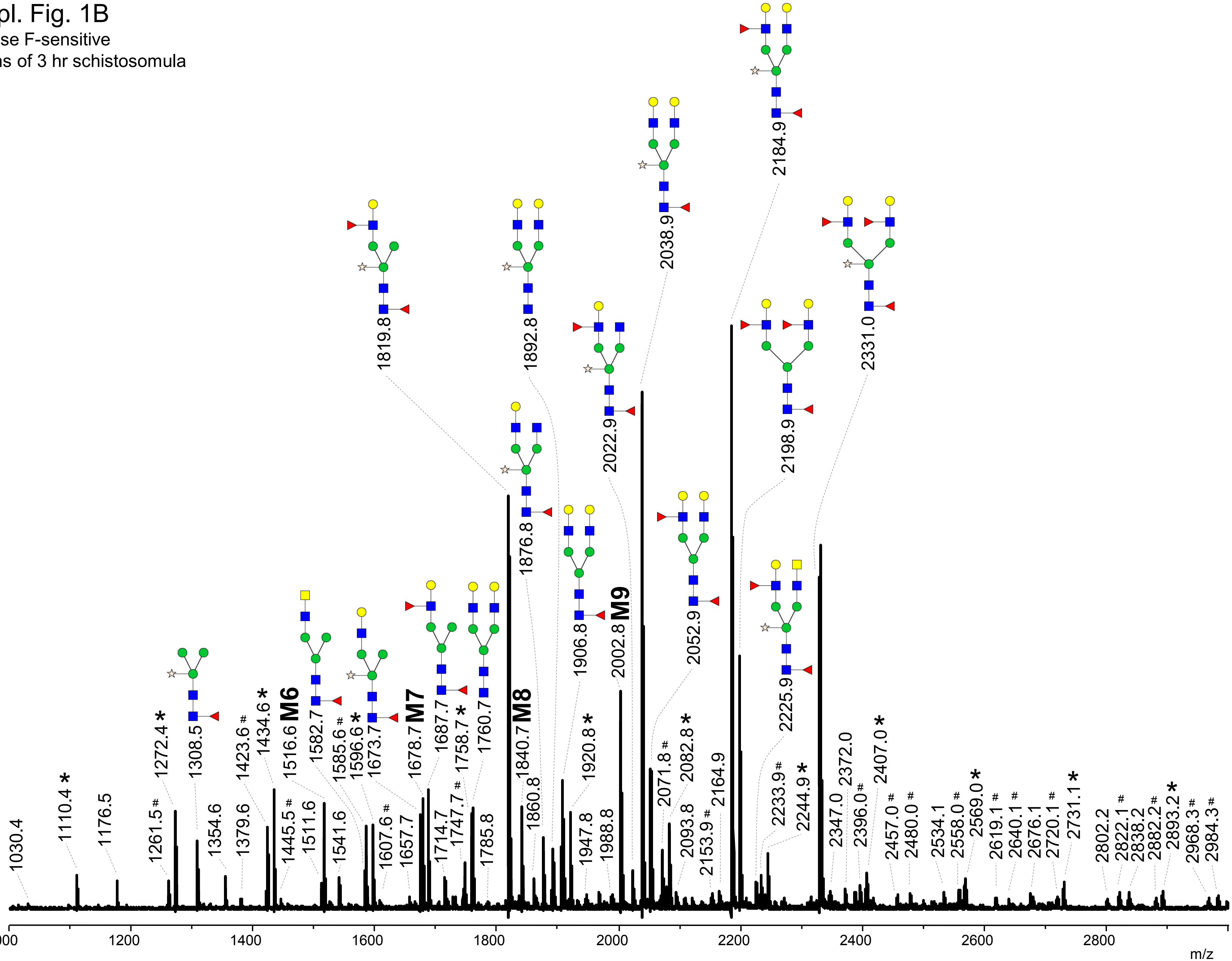

Suppl. Fig. 1C
PNGase F-sensitive
glycans of 24 hr schistosomula

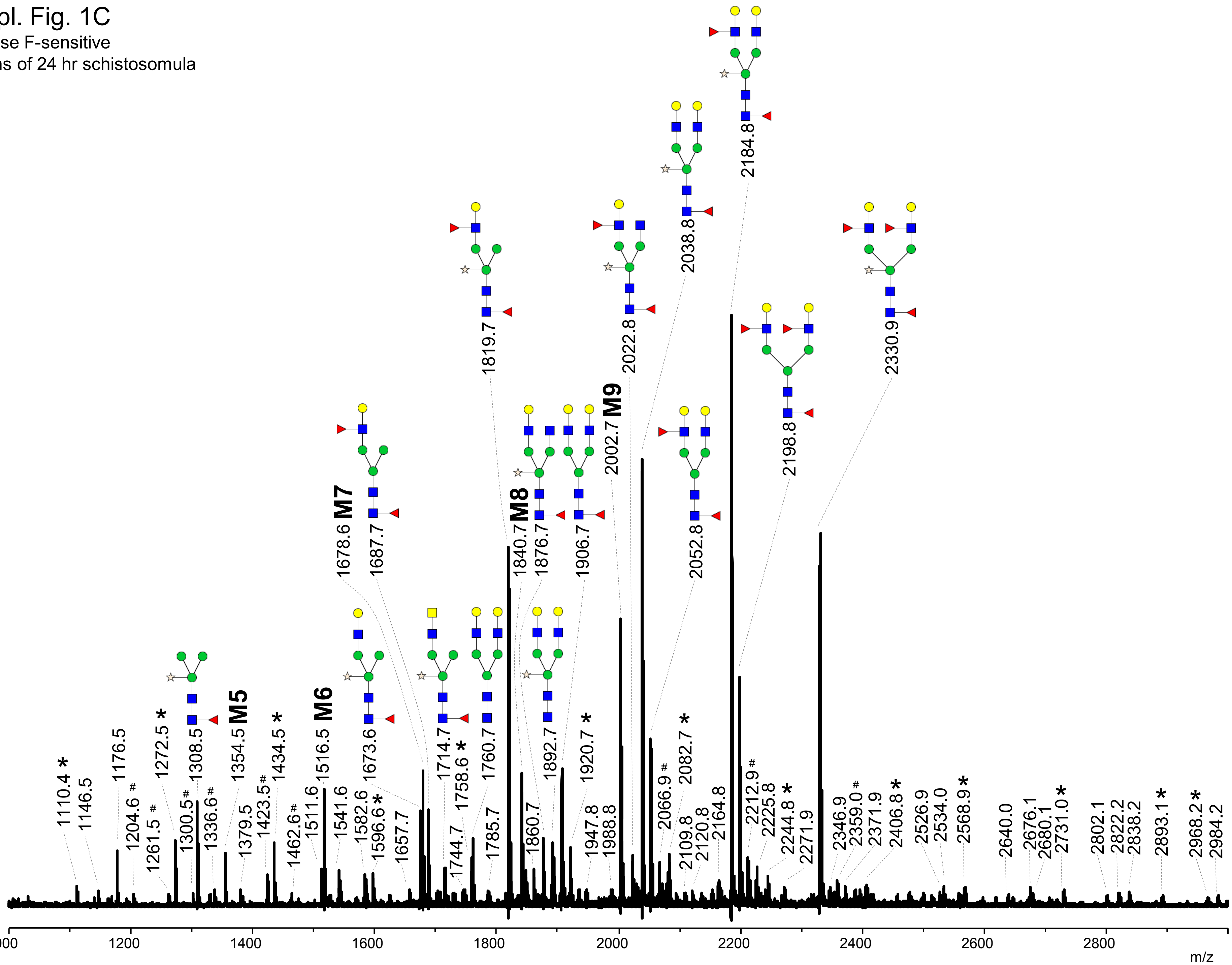

Suppl. Fig. 1D
PNGase F-sensitive
glycans of 3 days schistosomula

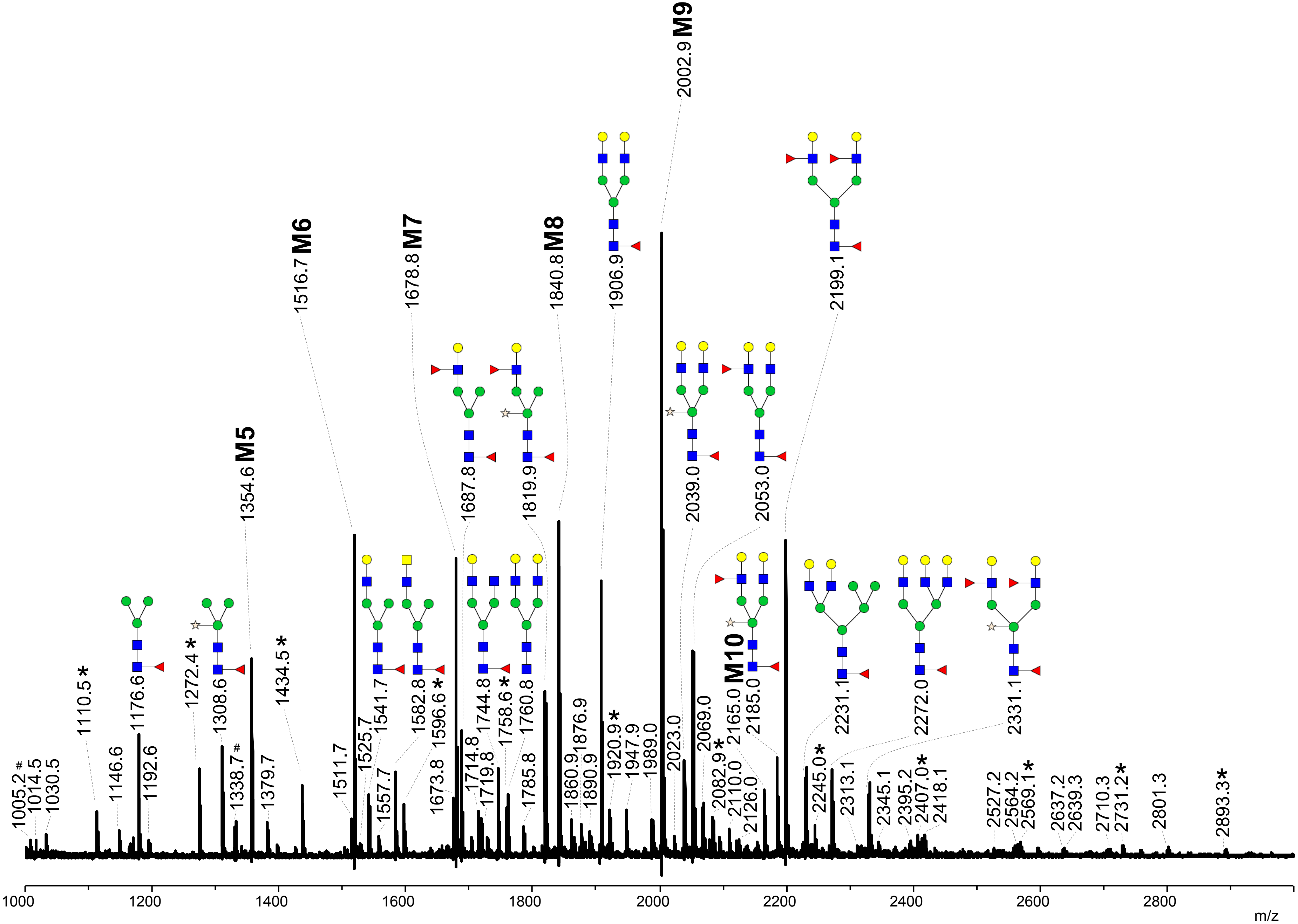

Suppl. Fig. 1E
PNGase F-sensitive
glycans of 6 days schistosomula

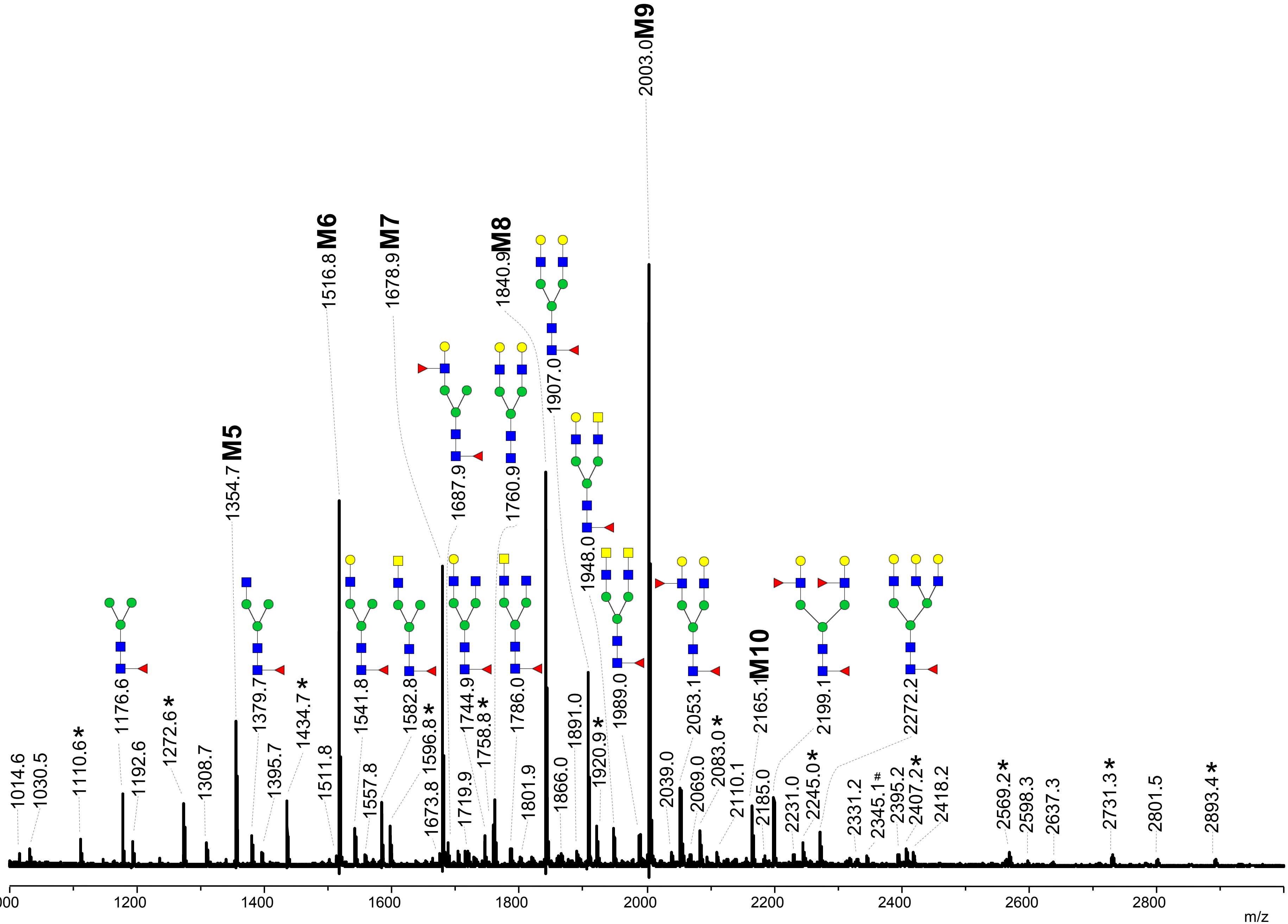

Suppl. Fig. 1F
PNGase F-sensitive
glycans of 2 weeks worms

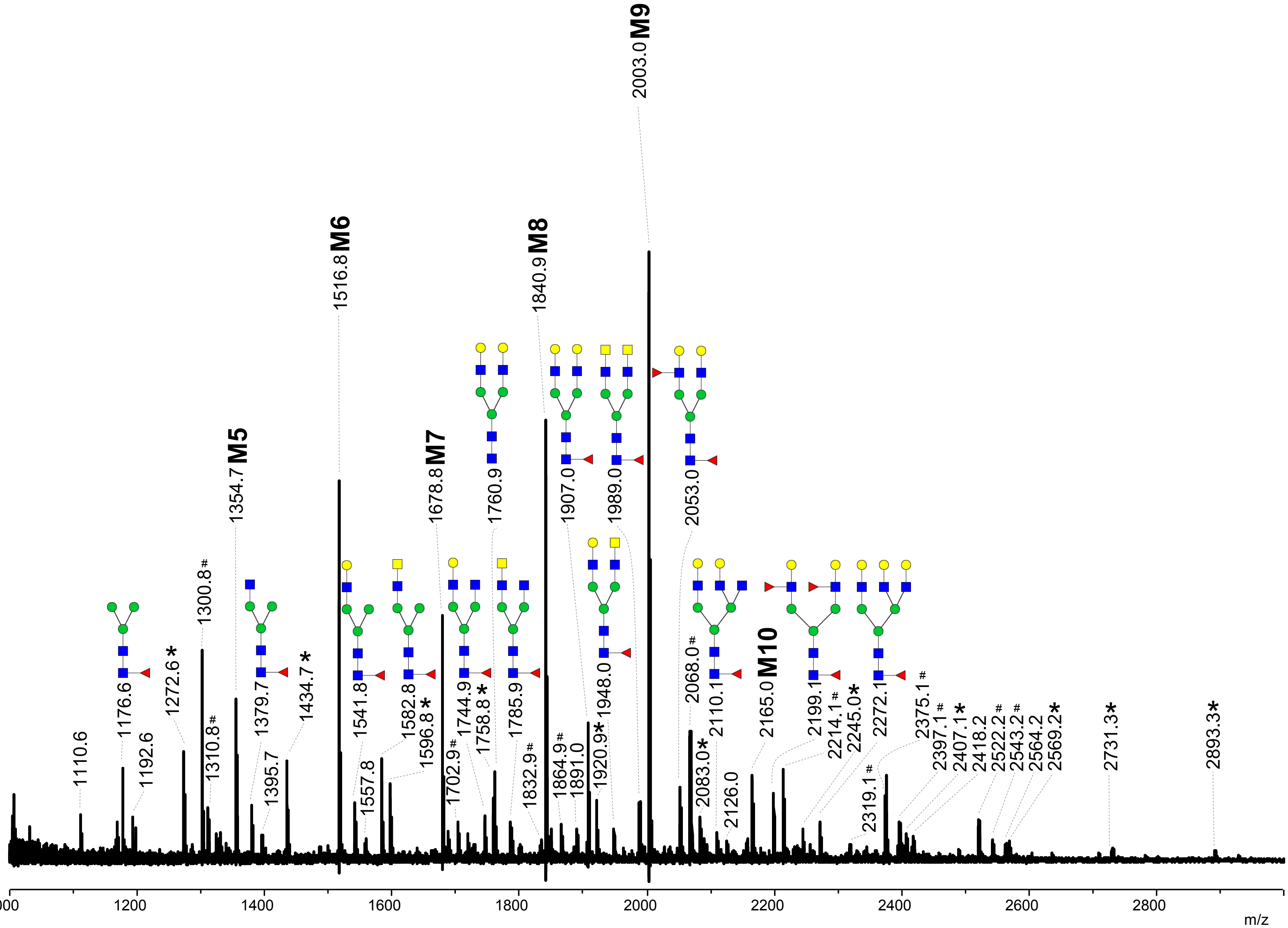

Suppl. Fig. 1G
PNGase F-sensitive
glycans of 3 weeks worms

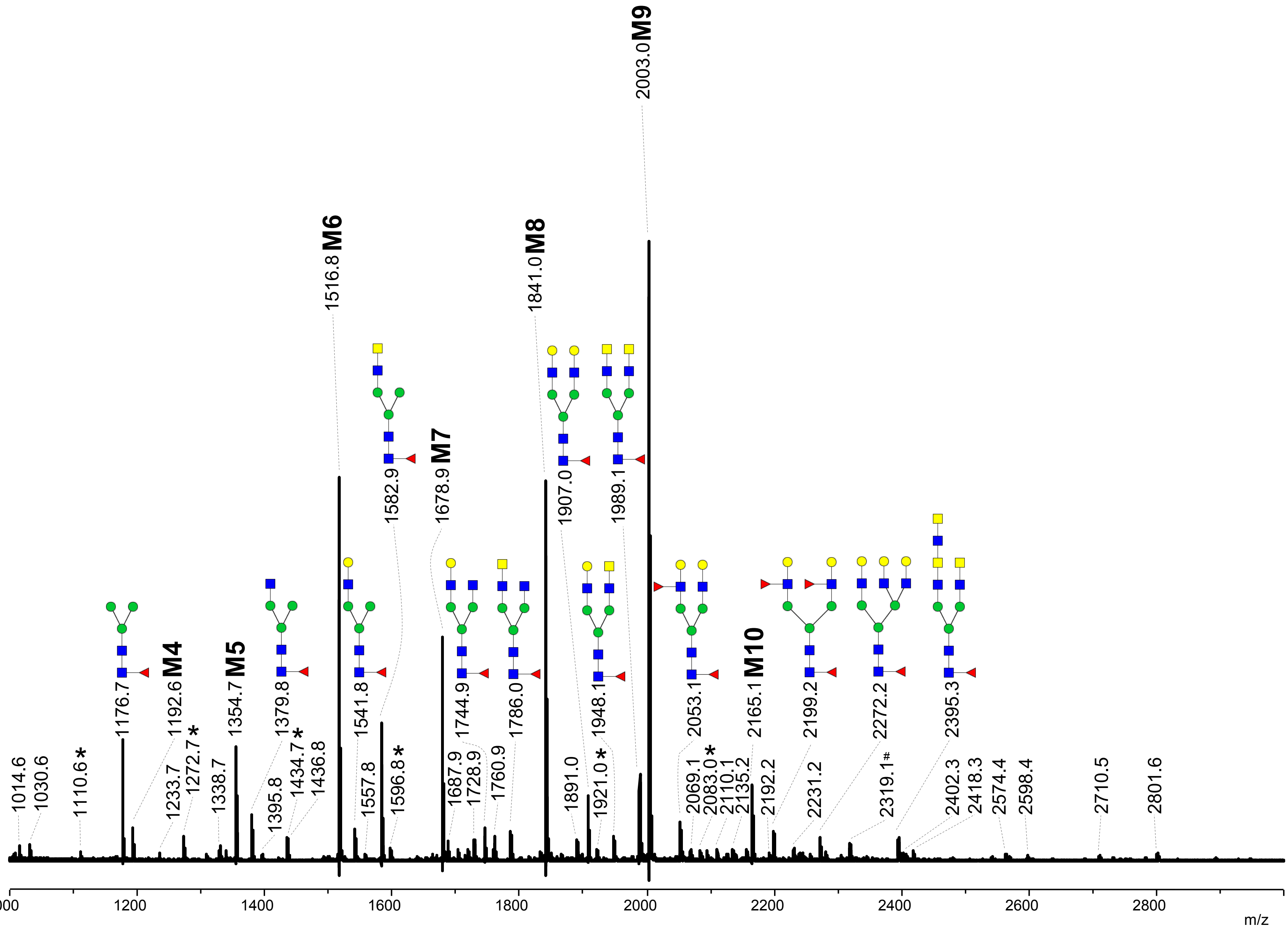

Suppl. Fig. 1H
PNGase F-sensitive
glycans of 4 weeks worms

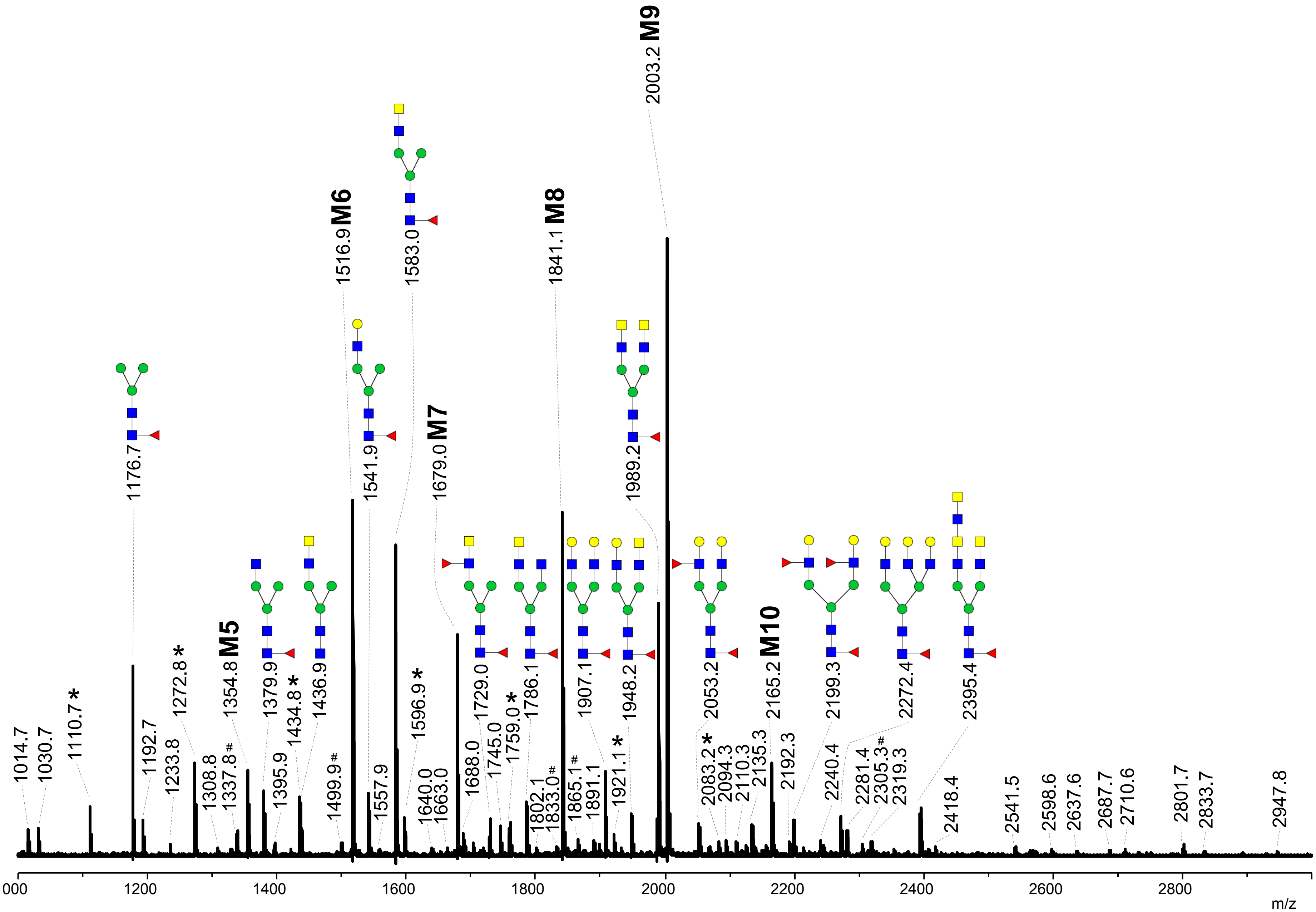

Suppl. Fig. 1I

PNGase F-sensitive
glycans of 5 weeks worms

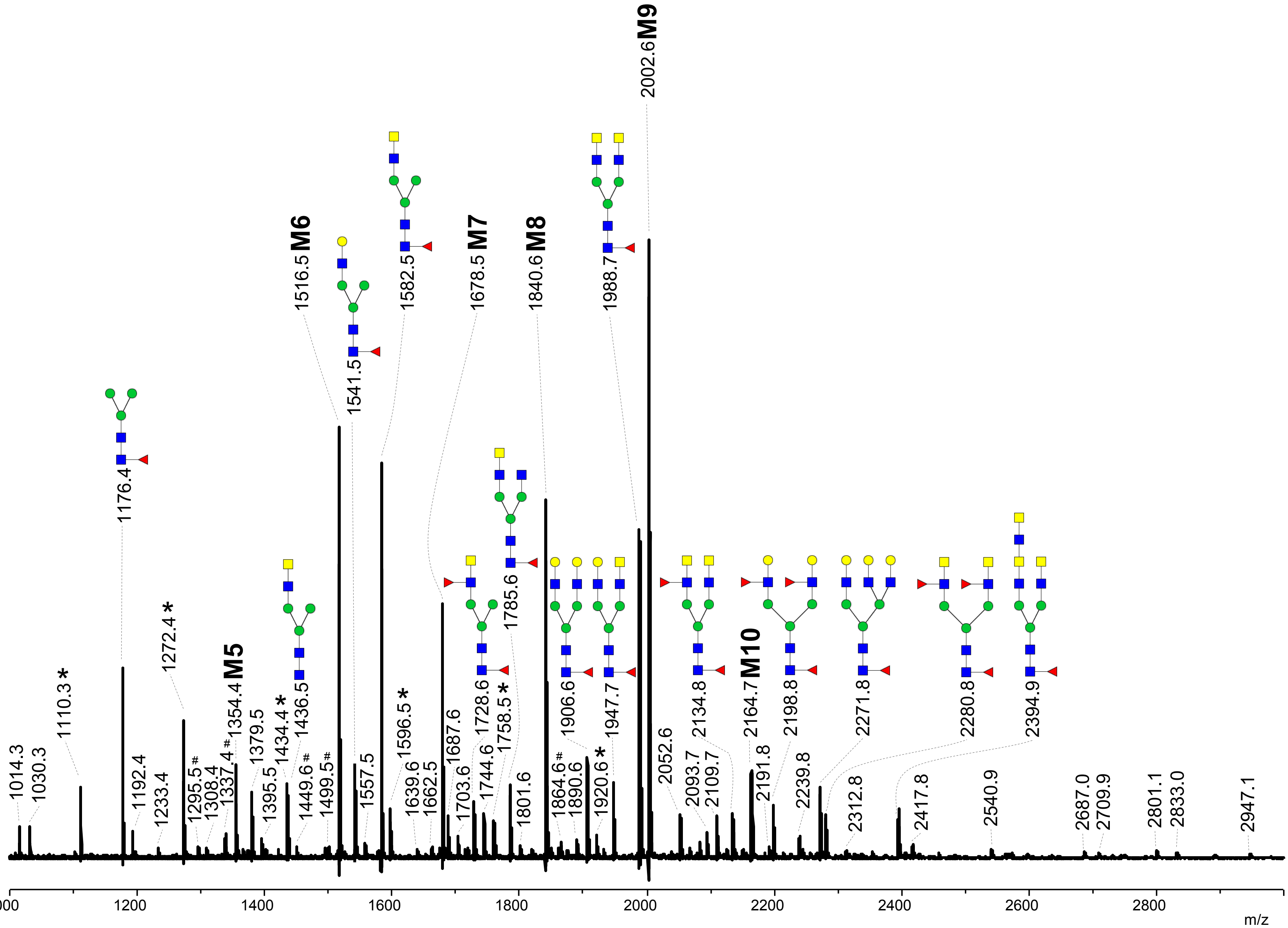

Suppl. Fig. 1J
PNGase F-sensitive
glycans of 6 weeks worms

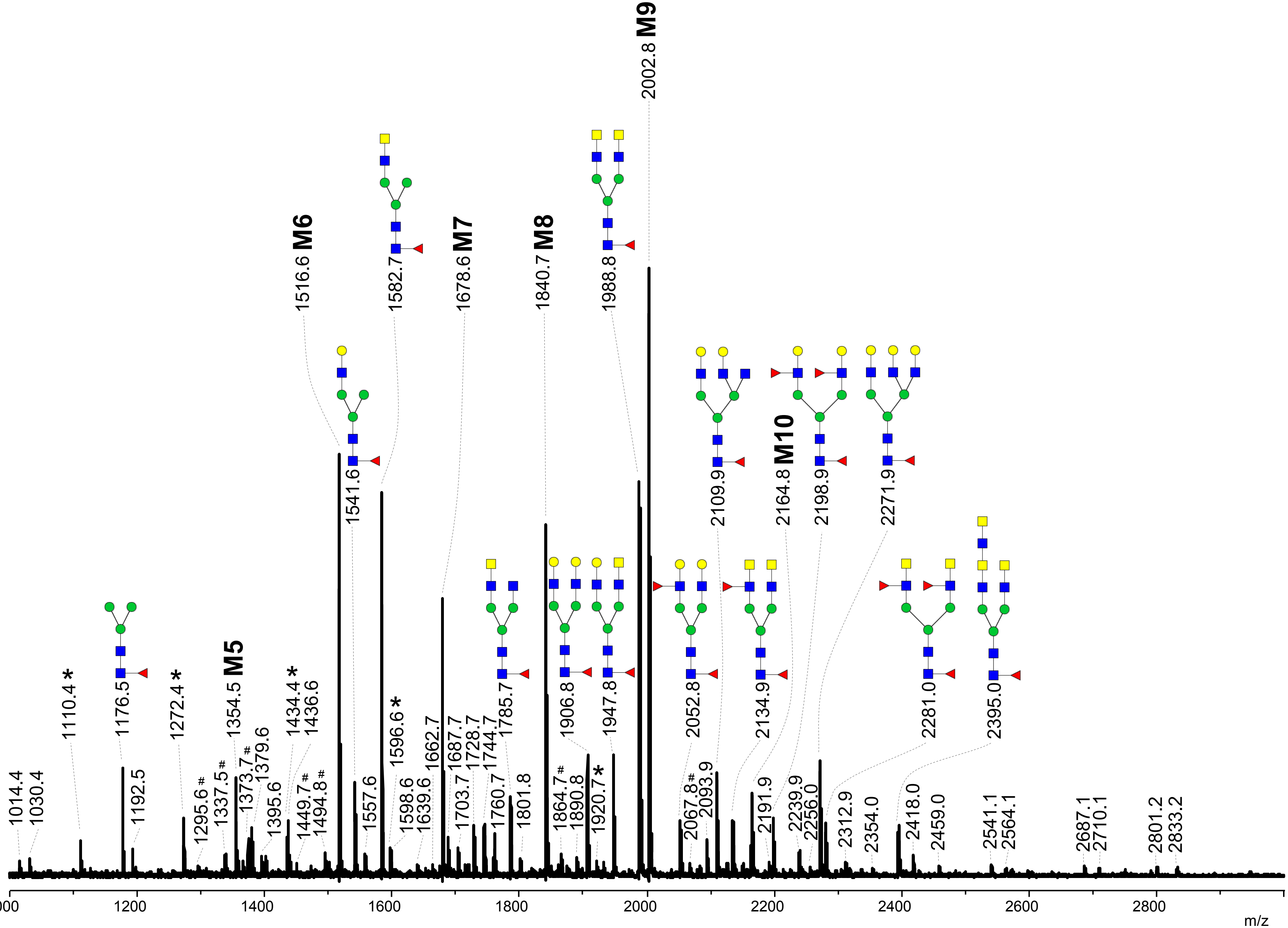

Supplement: Supplemental Data [file supp_M115.048280_mcp.M115.048280-2.pdf]
