# Supplementary material for: Glycomic Analysis of Life Stages of the Human Parasite Schistosoma mansoni Reveals Developmental Expression Profiles of Functional and Antigenic Glycan Motifs
Source: Mol Cell Proteomics. 2015 Apr 16;14(7):1750–69. doi: 10.1074/mcp.M115.048280 (PMC4587318; doi:10.1074/mcp.M115.048280)

Suppl. Fig. 2A
permethylated O-glycans
of cercariae

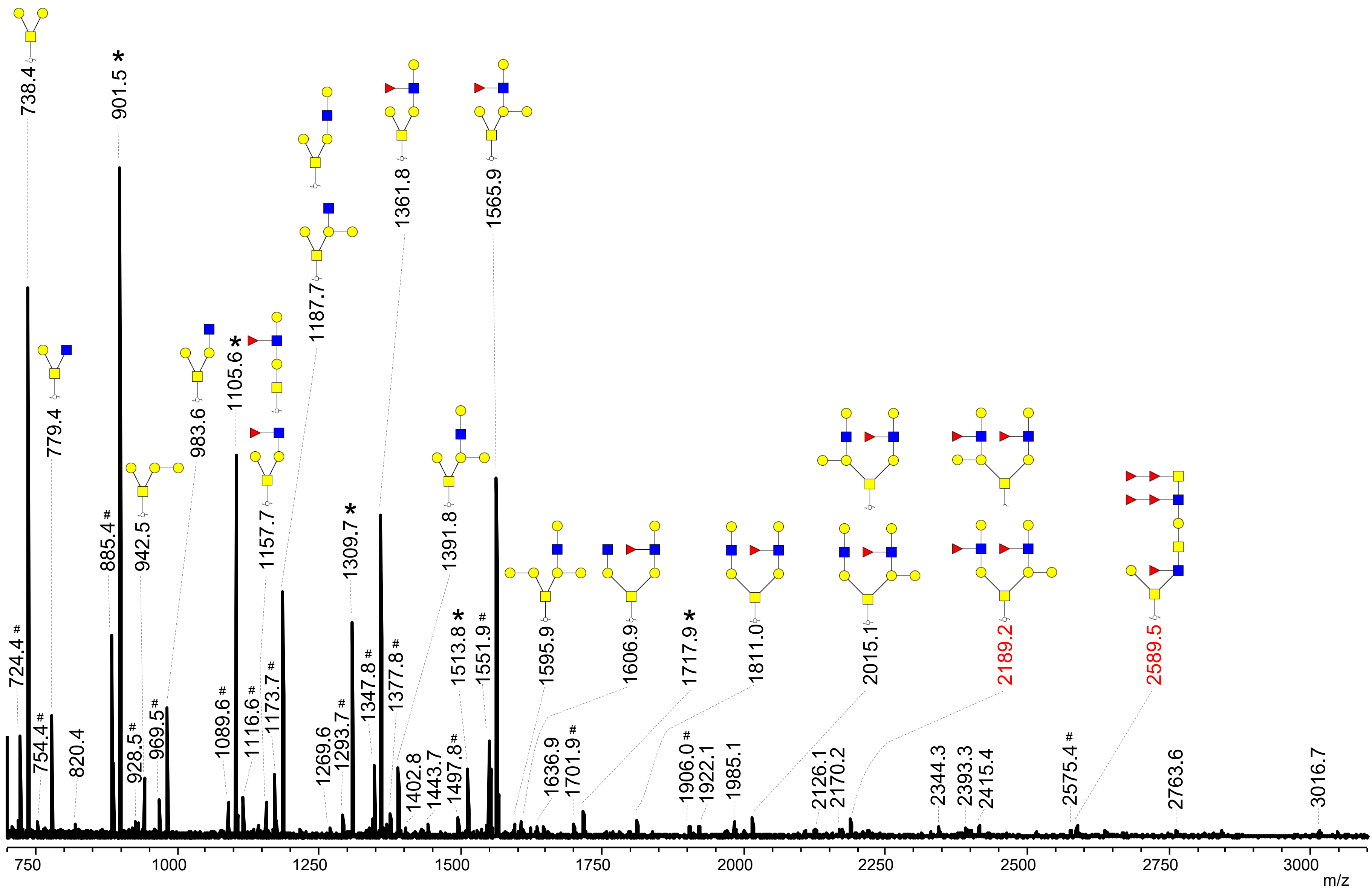

Suppl. Fig. 2B

permethylated O-glycans of 3h schistosomula

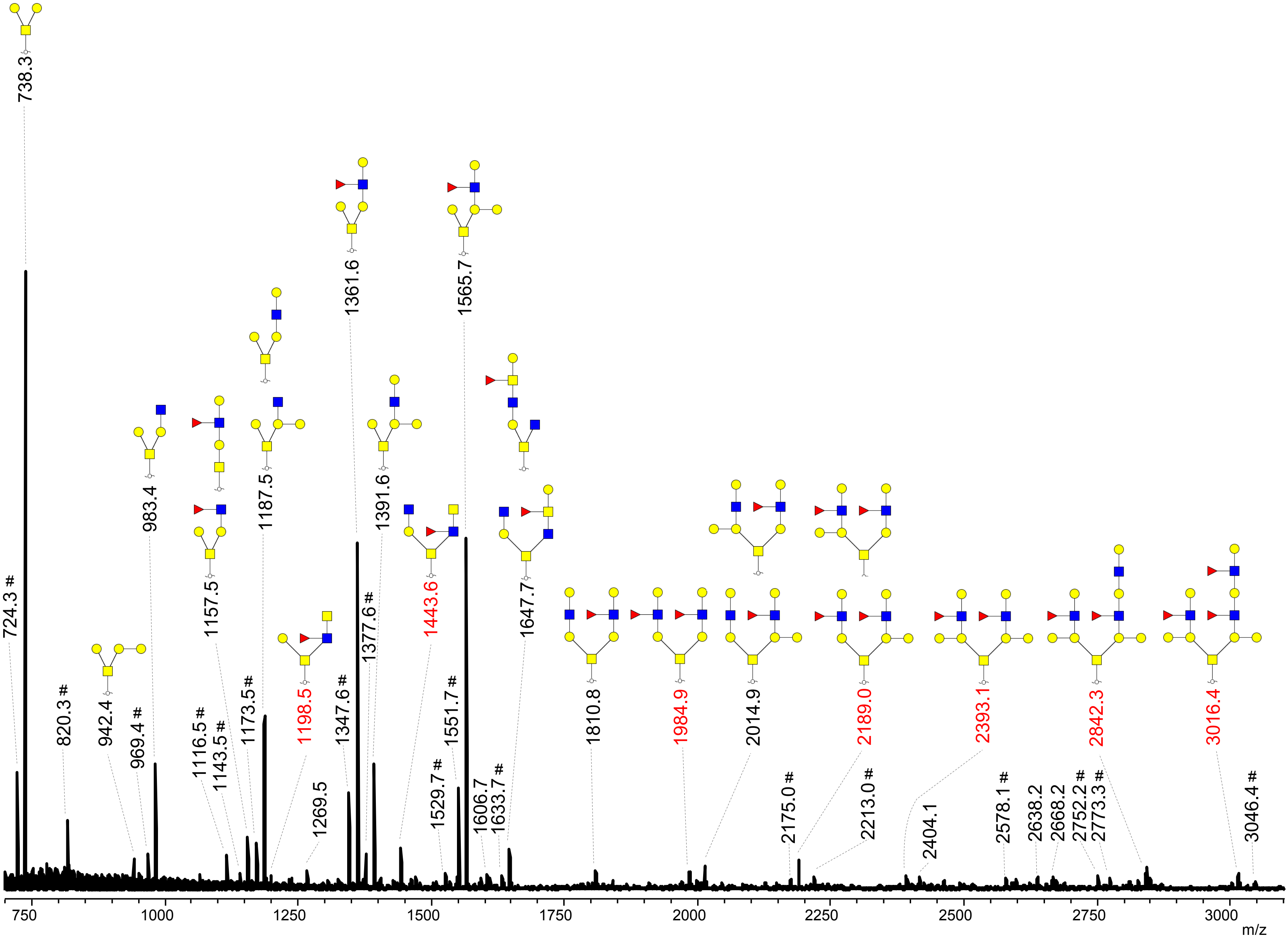

Suppl. Fig. 2C
permethylated O-glycans
of 24h schistosomula

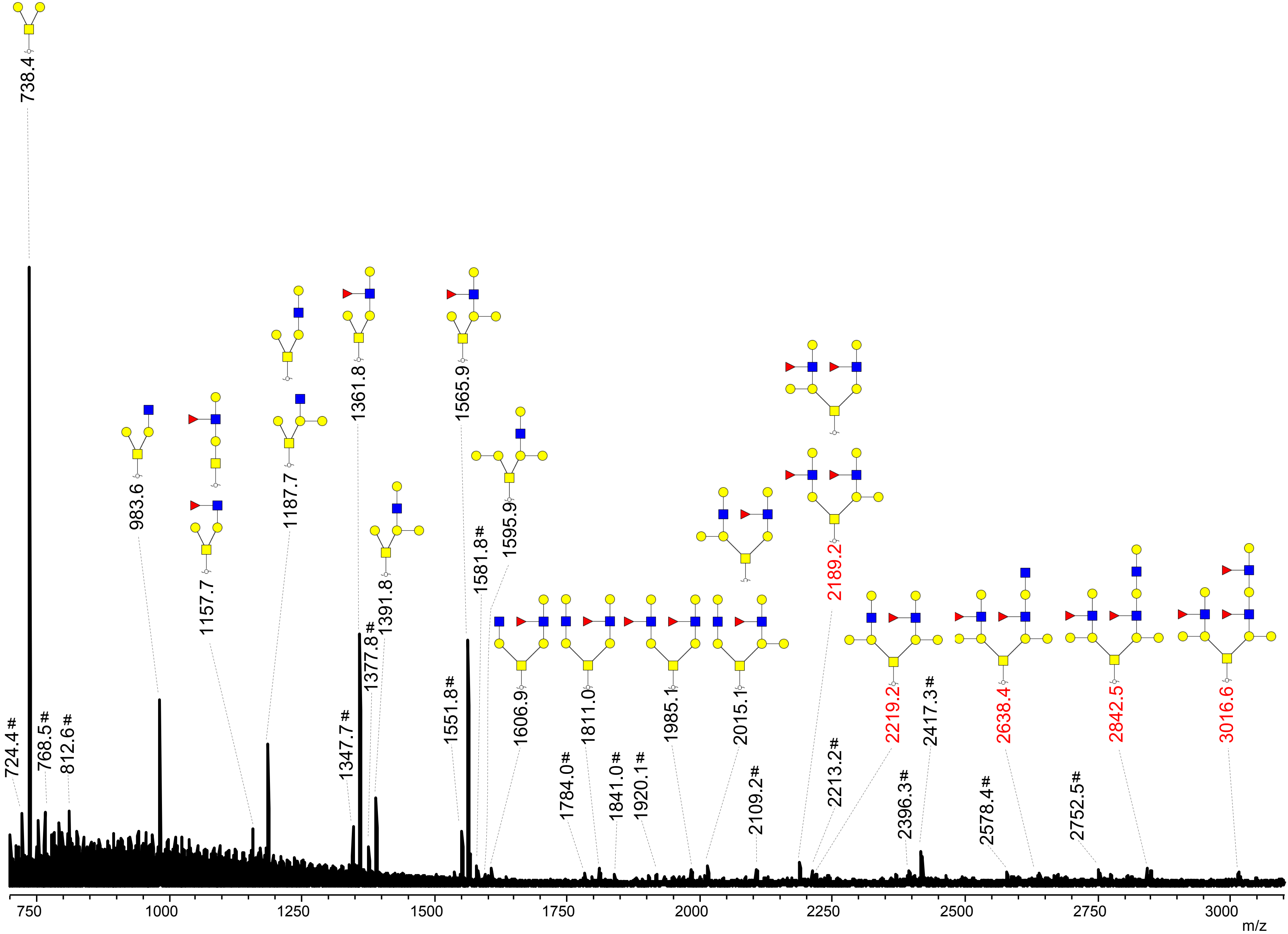

Suppl. Fig. 2D
permethylated O-glycans
of 48h schistosomula

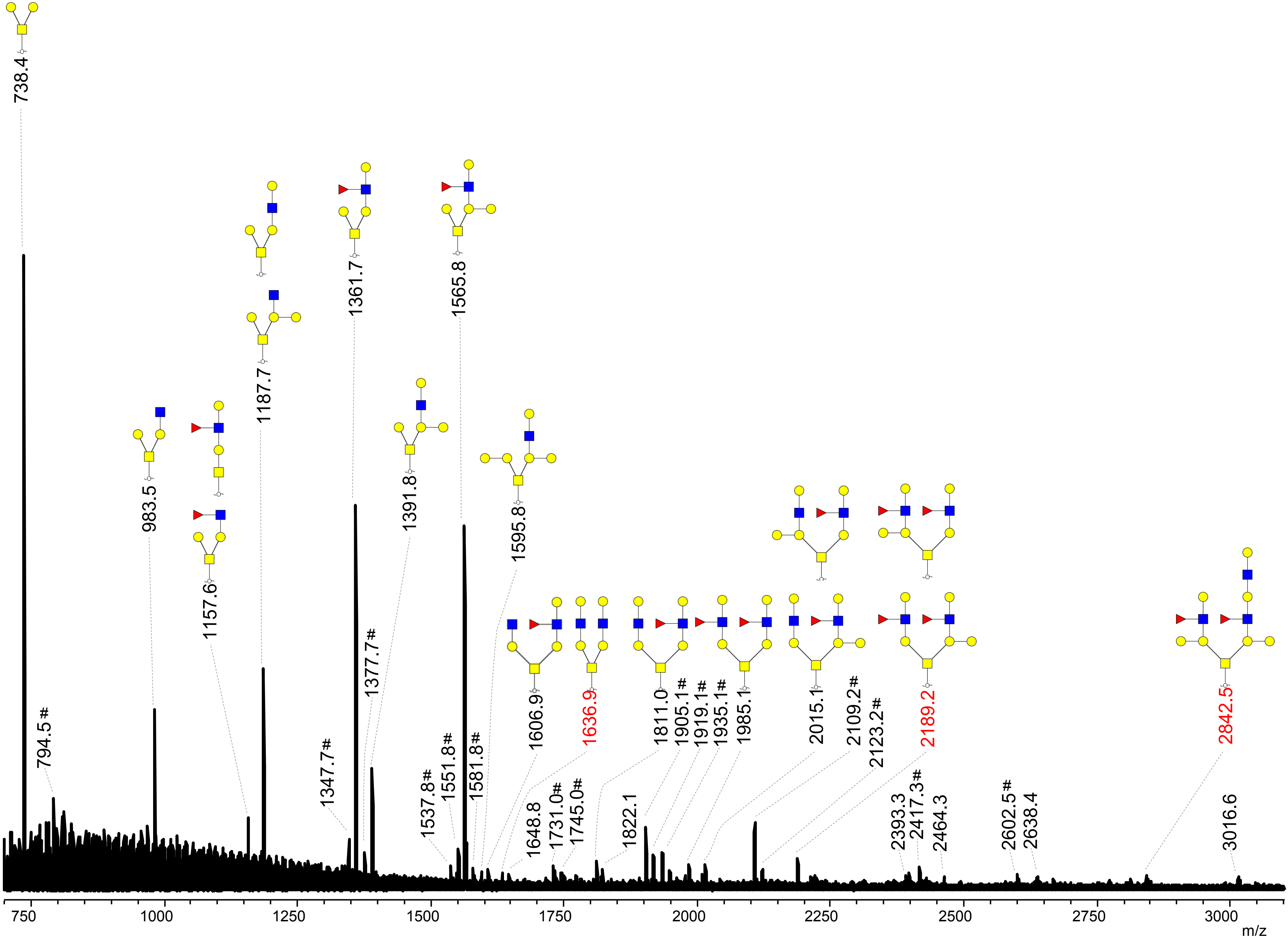

Suppl. Fig. 2E
permethylated O-glycans
of 3 days schistosomula

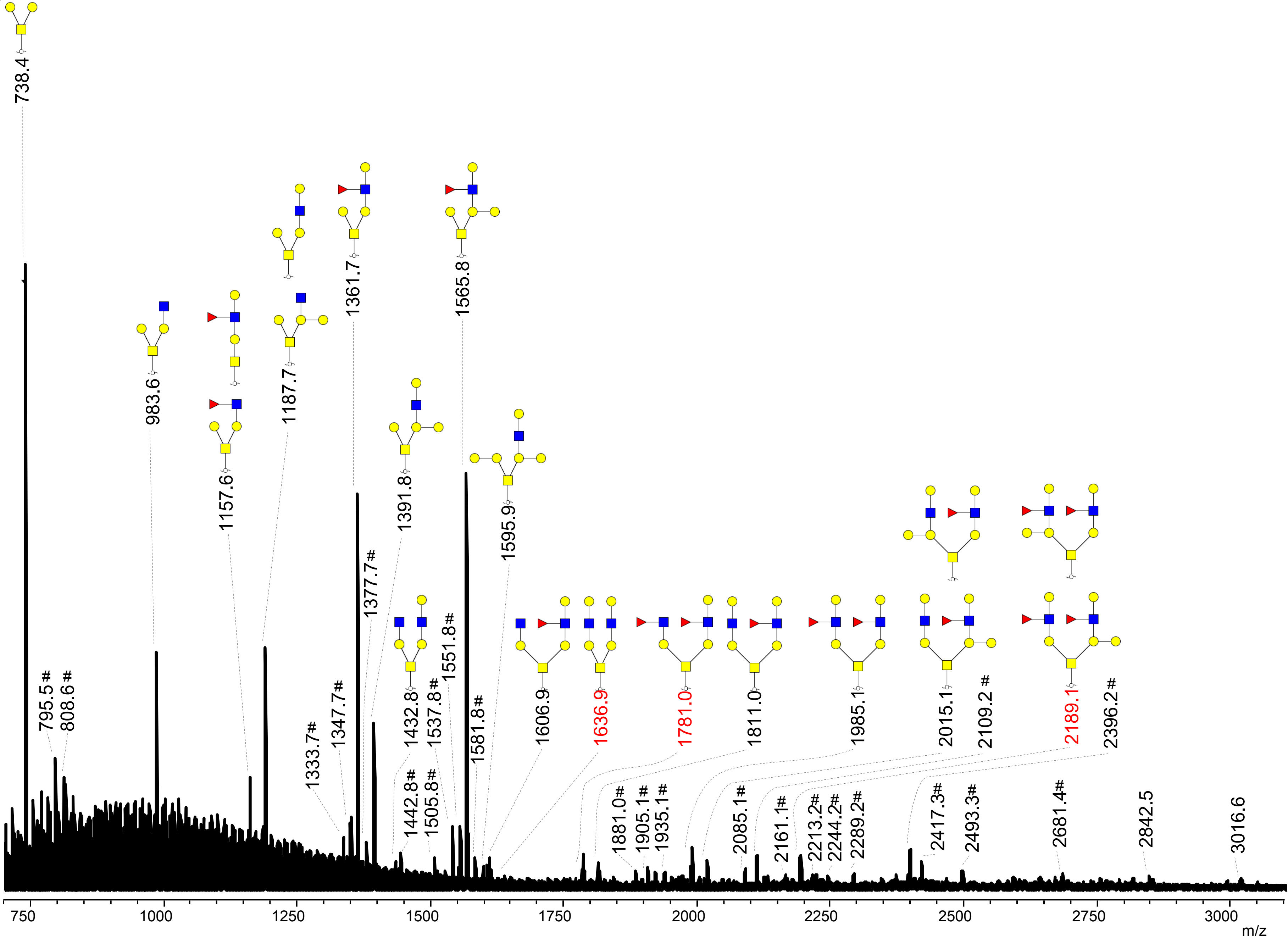

Suppl. Fig. 2F
permethylated O-glycans
of immature eggs

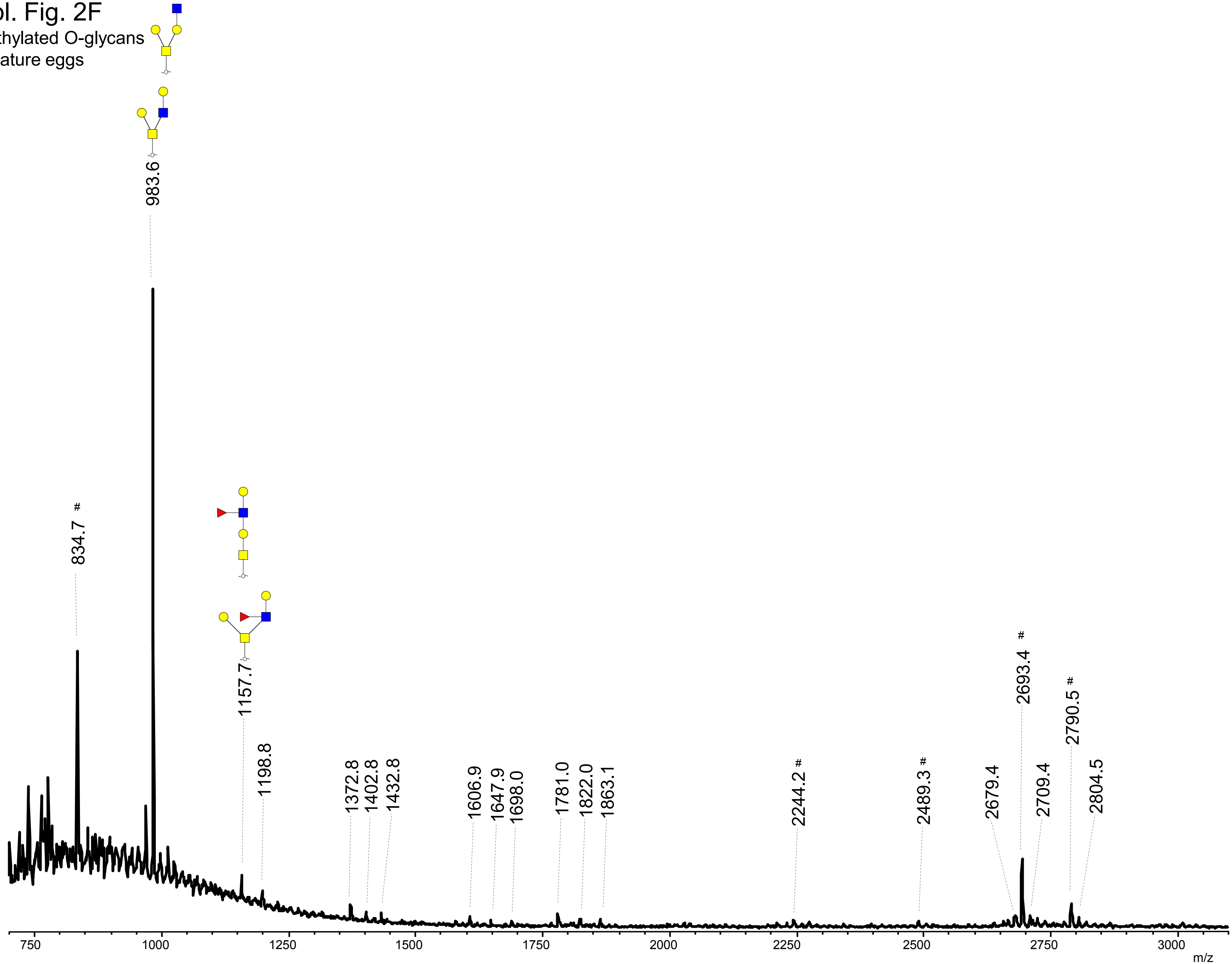

Suppl. Fig. 2G
permethylated O-glycans
of mature eggs

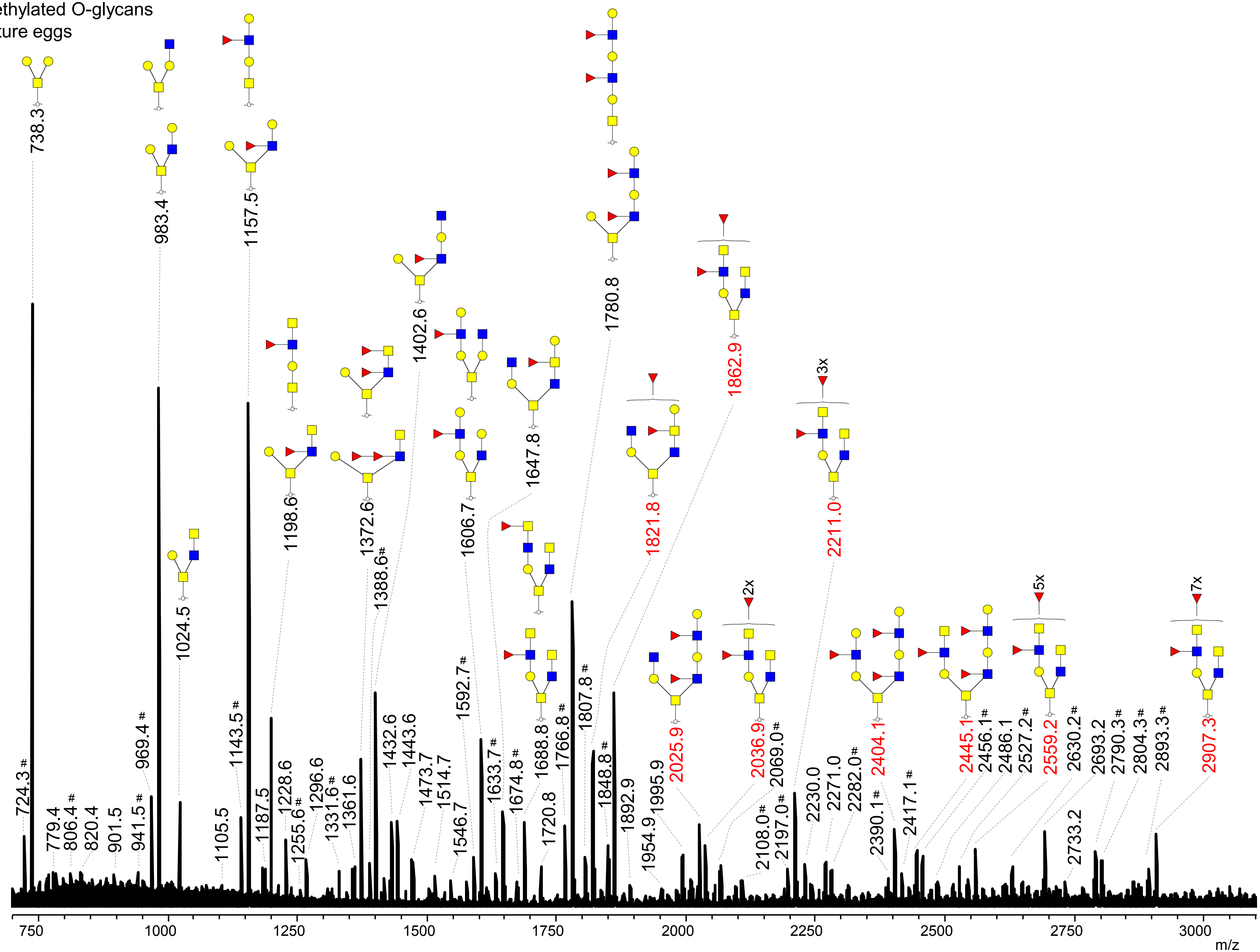

Suppl. Fig. 2H

permethylated O-glycans
of miracidia

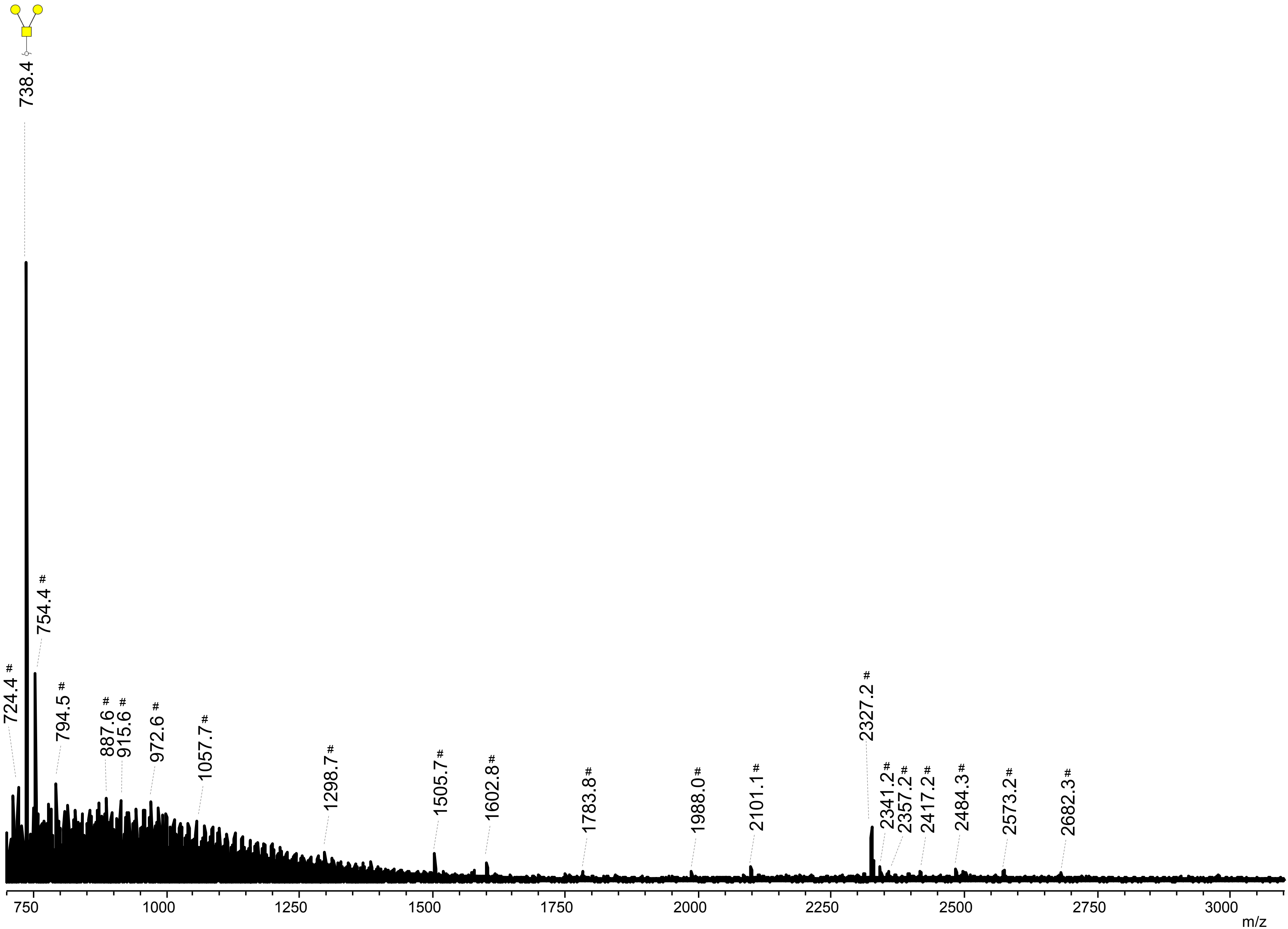

Supplement: Supplemental Data [file supp_M115.048280_mcp.M115.048280-3.pdf]
