# Supplementary material for: Glycomic Analysis of Life Stages of the Human Parasite Schistosoma mansoni Reveals Developmental Expression Profiles of Functional and Antigenic Glycan Motifs
Source: Mol Cell Proteomics. 2015 Apr 16;14(7):1750–69. doi: 10.1074/mcp.M115.048280 (PMC4587318; doi:10.1074/mcp.M115.048280)

Suppl. Fig. 3A

MALDI-TOF-MS/MS analysis of permethylated O-glycan at m/z 1187[M+Na+] H3N2 found in cercariae

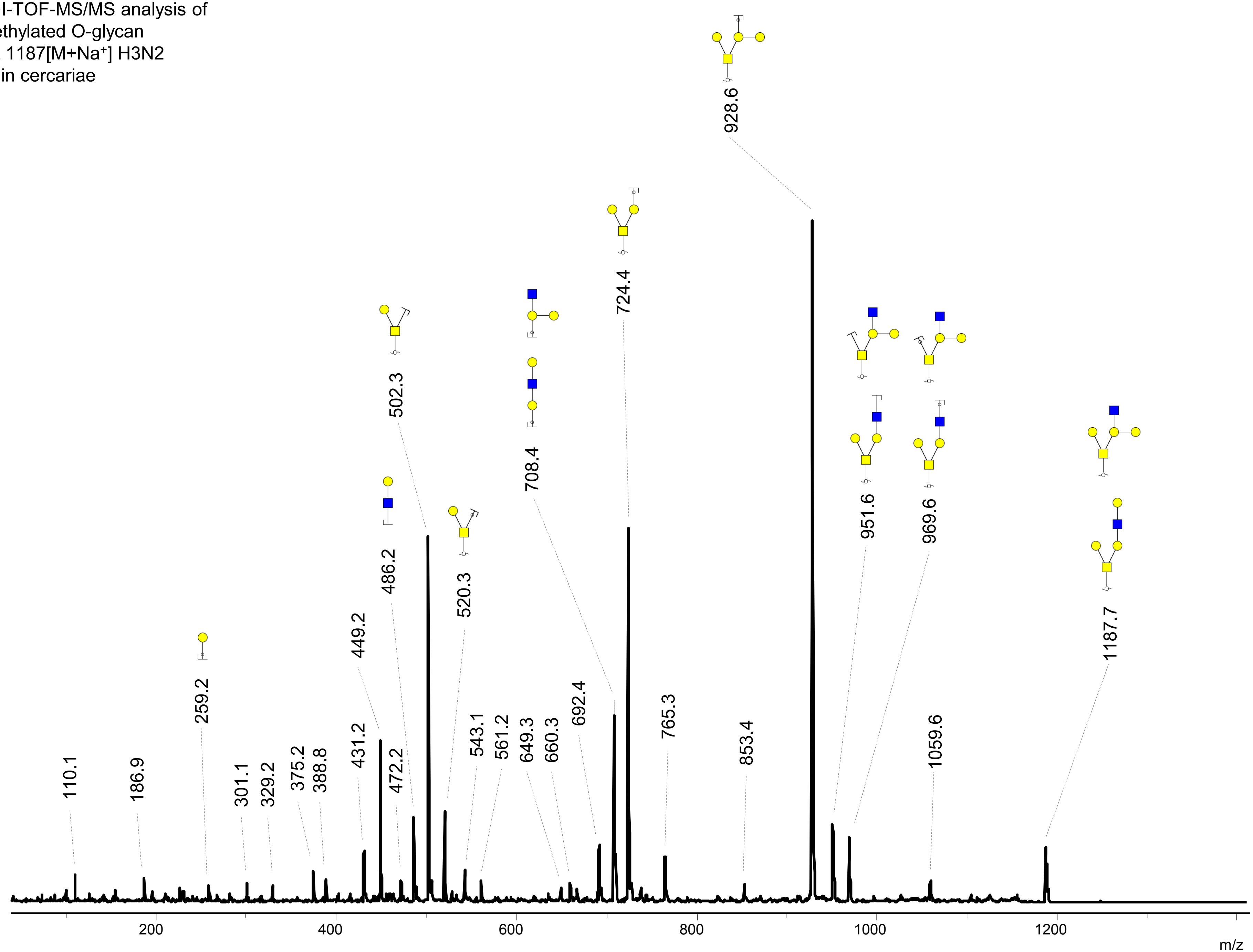

Suppl. Fig. 3B
MALDI-TOF-MS/MS analysis of
permethylated O-glycan
at 1361[M+Na+] F1H3N2
found in cercariae

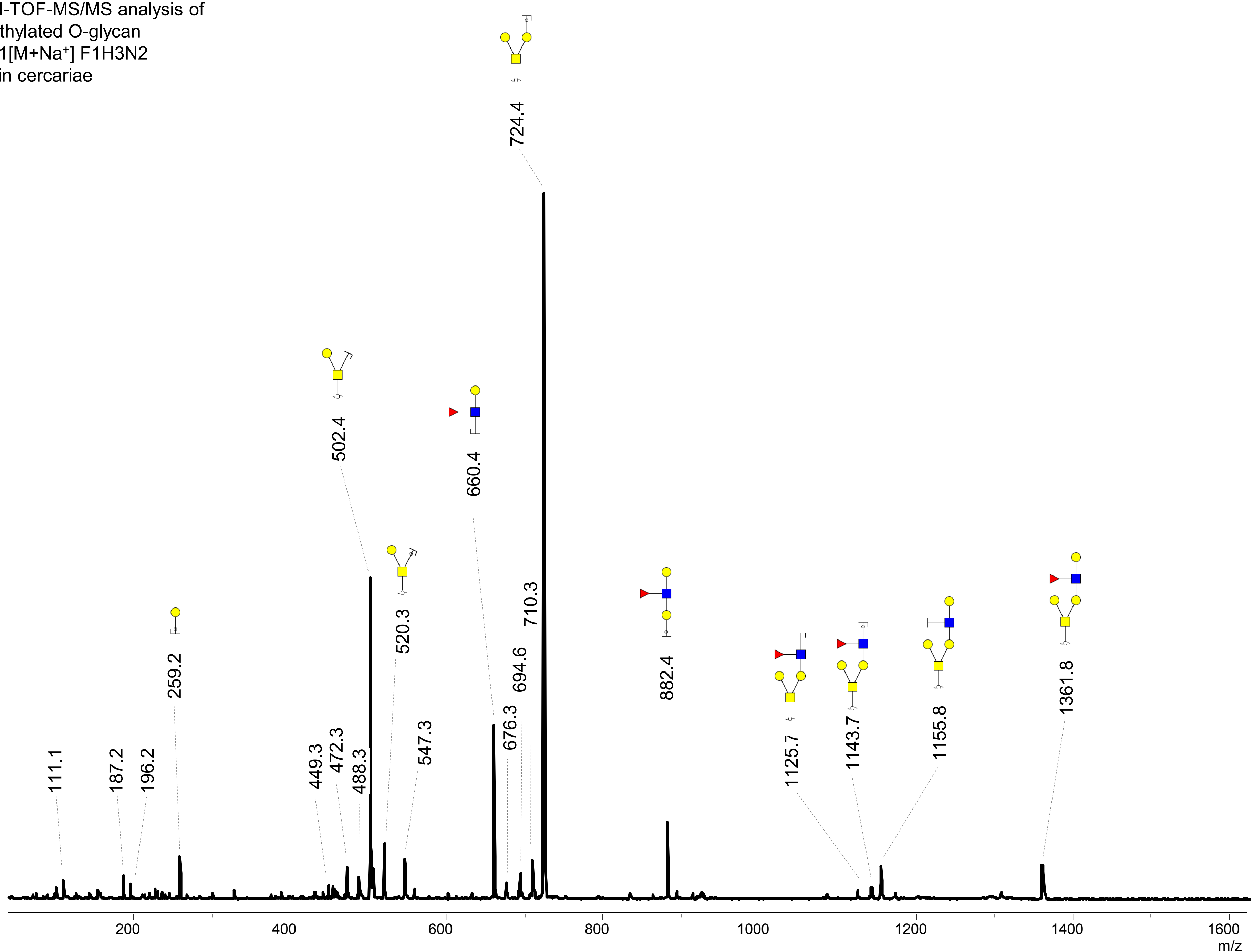

Suppl. Fig. 3C

MALDI-TOF-MS/MS analysis of permethylated O-glycan at 1565[M+Na$^+$] F1H4N2 found in cercariae

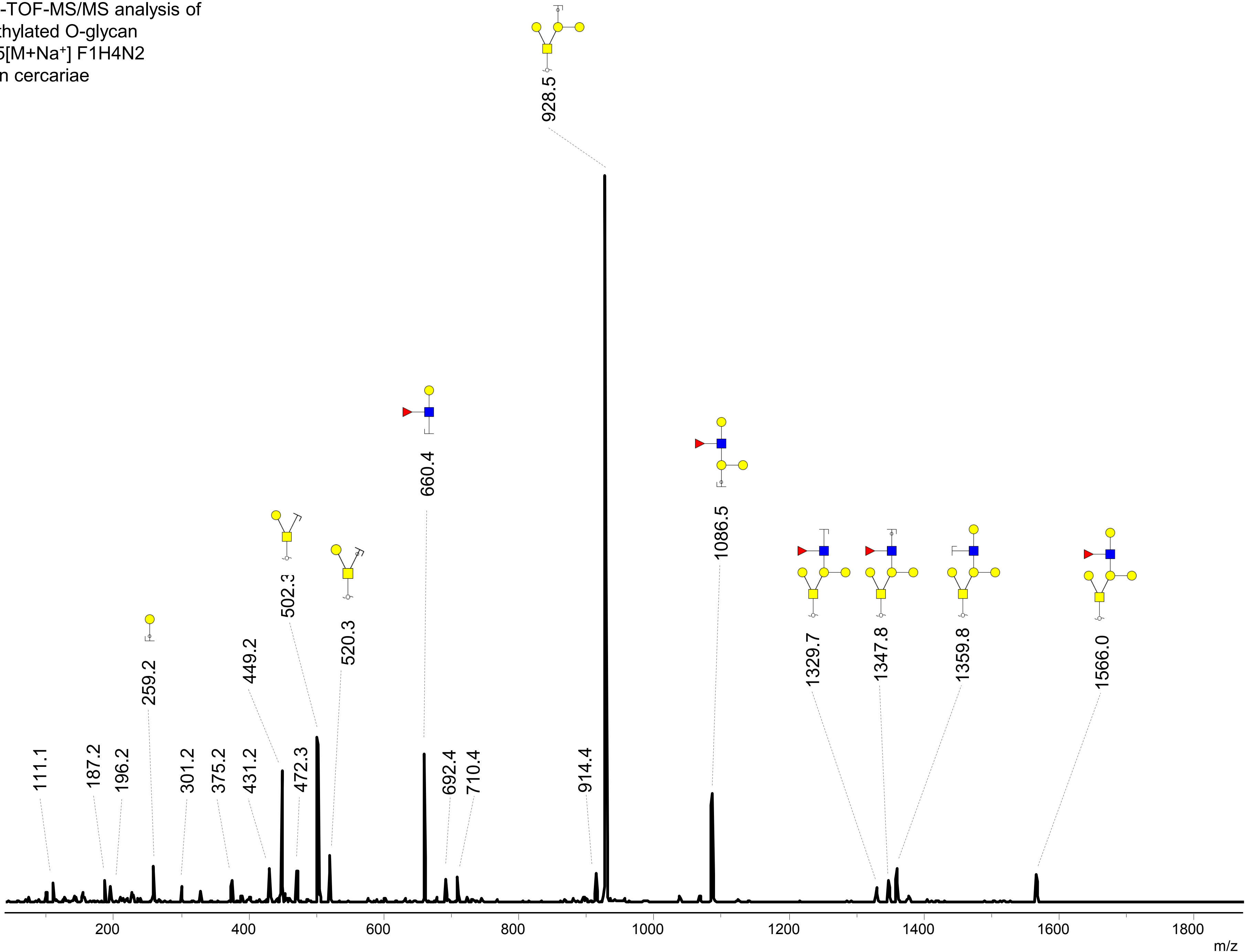

Suppl. Fig. 3D
MALDI-TOF-MS/MS analysis of
permethylated O-glycan
at 1198[M+Na^{+}] F1H1N3
found in eggs

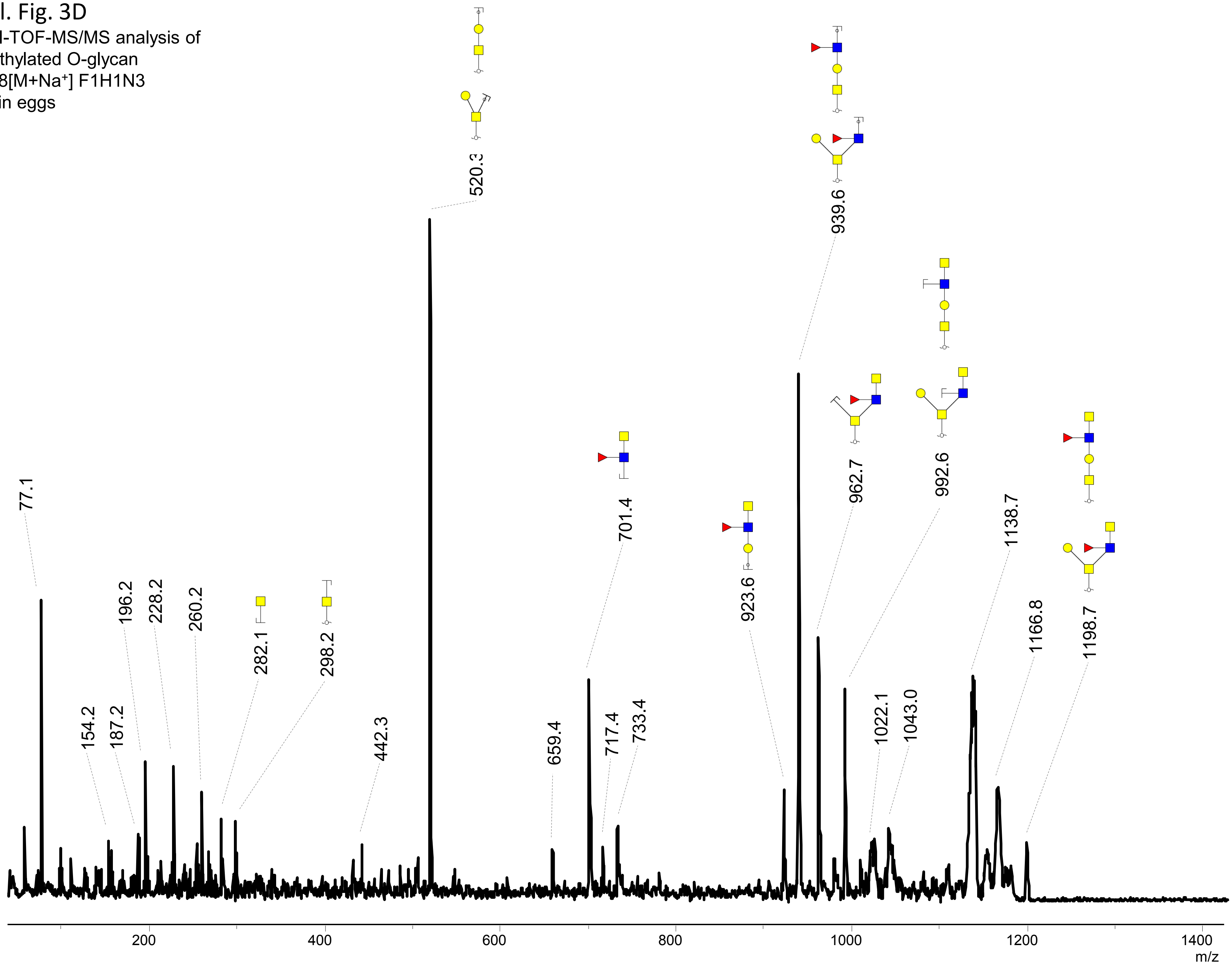

Suppl. Fig. 3E
MALDI-TOF-MS/MS analysis of
permethylated O-glycan
at 1606[M+Na$^+$] F1H3N3
found in eggs

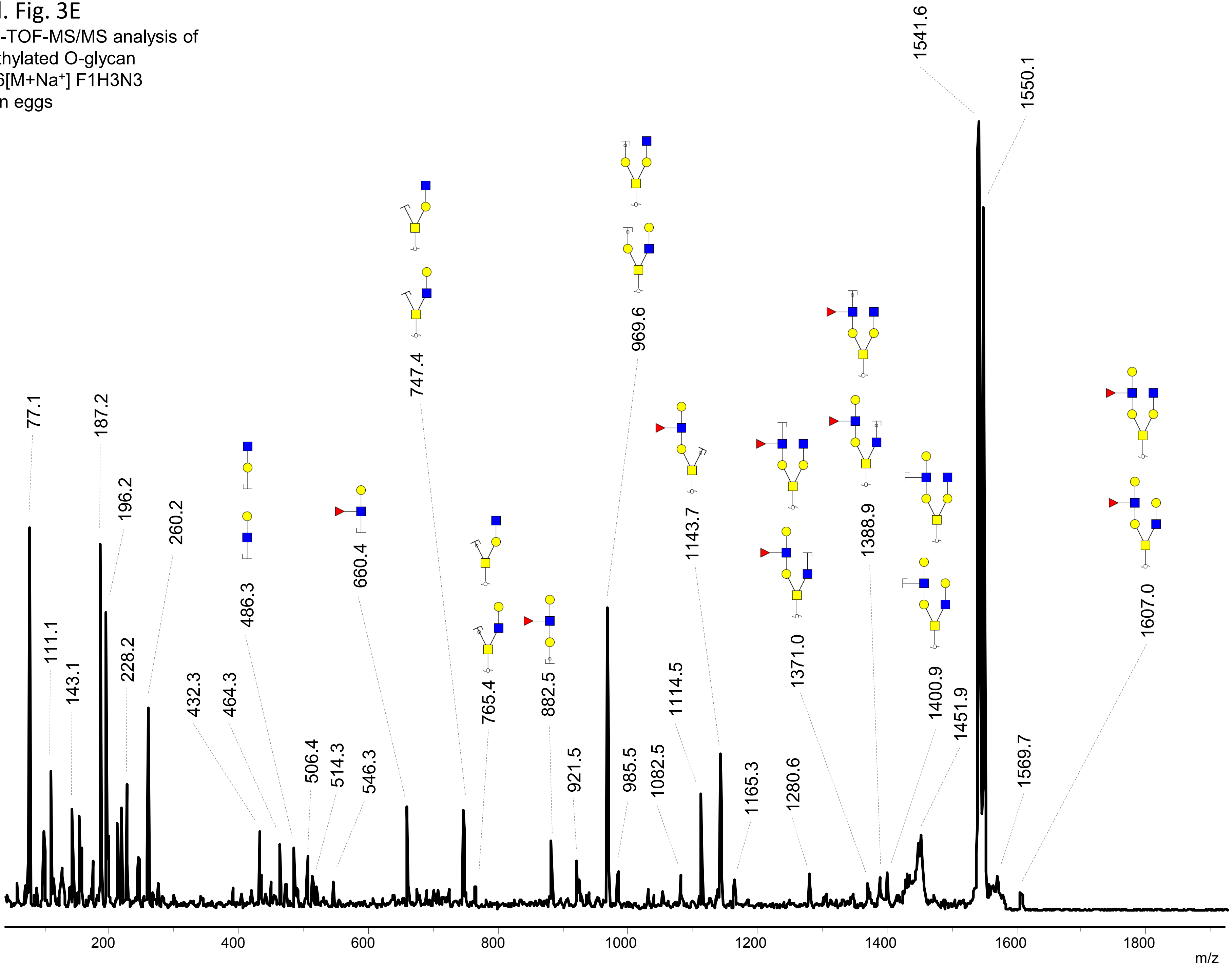

Suppl. Fig. 3F

MALDI-TOF-MS/MS analysis of
permethylated O-glycan
at 738[M+Na+] H2N1
found in miracidia

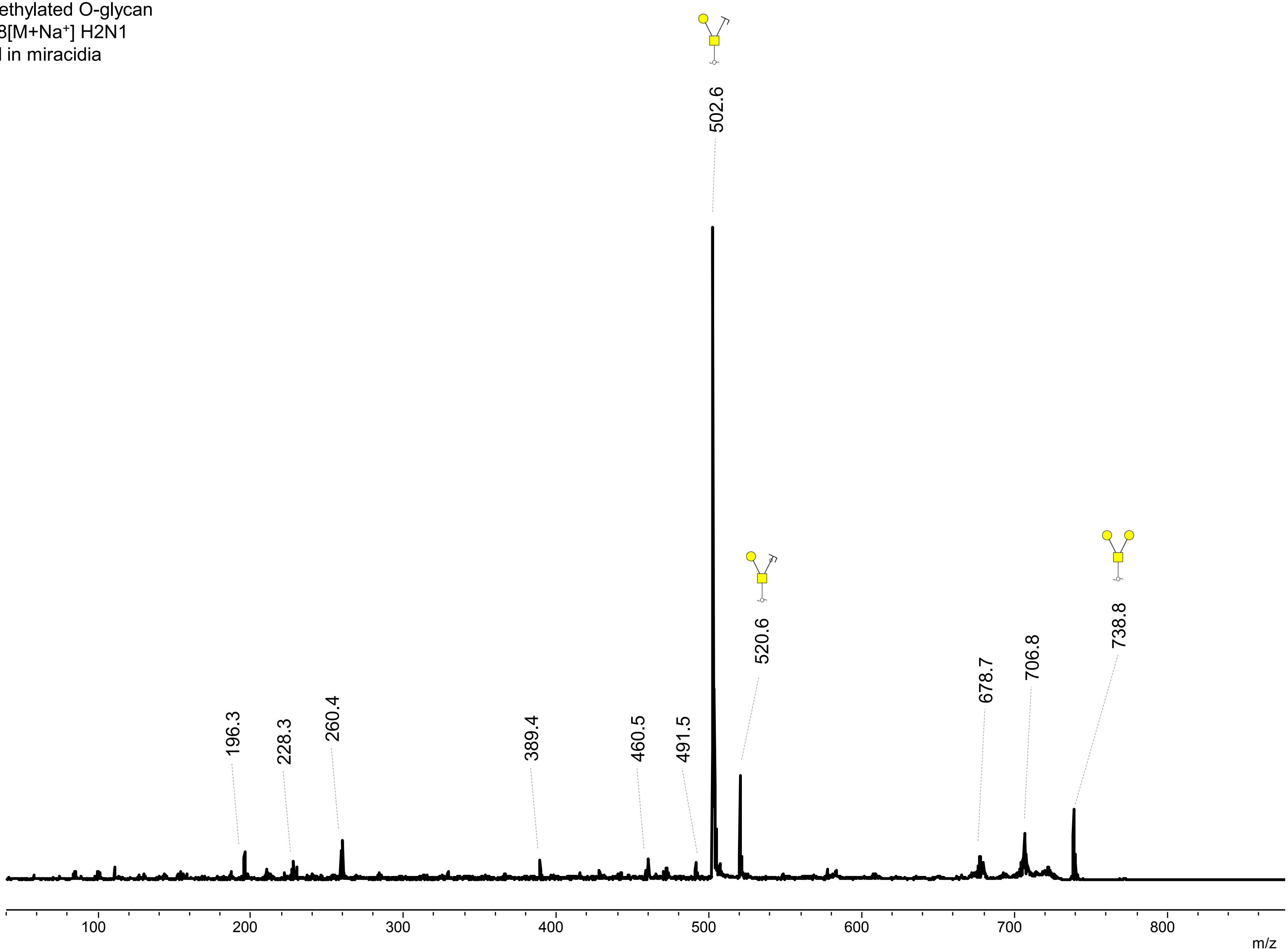

Supplement: Supplemental Data [file supp_M115.048280_mcp.M115.048280-4.pdf]
