# Supplementary material for: Glycomic Analysis of Life Stages of the Human Parasite Schistosoma mansoni Reveals Developmental Expression Profiles of Functional and Antigenic Glycan Motifs
Source: Mol Cell Proteomics. 2015 Apr 16;14(7):1750–69. doi: 10.1074/mcp.M115.048280 (PMC4587318; doi:10.1074/mcp.M115.048280)

Suppl. Fig. 4A
lipid glycans
of cercariae

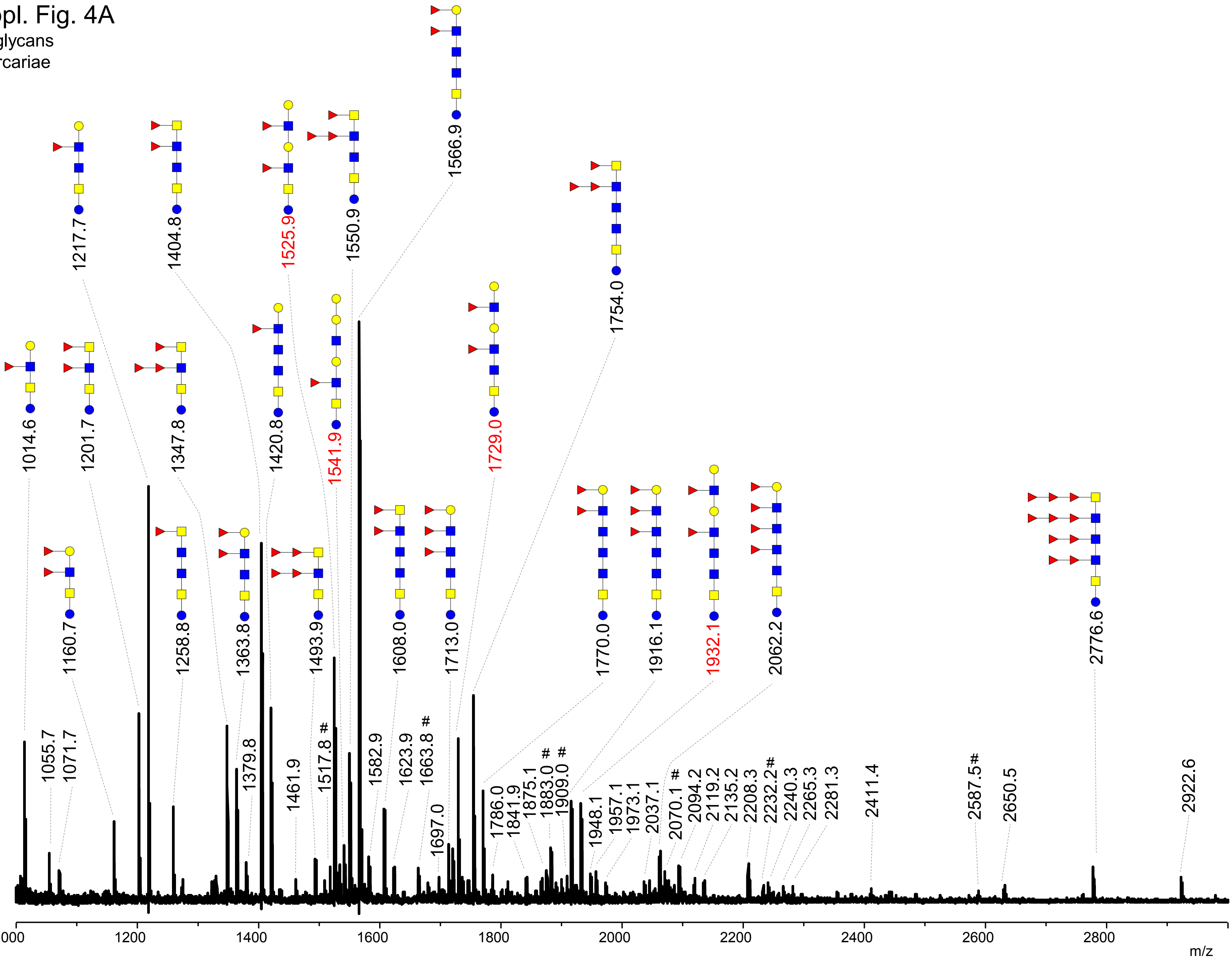

Suppl. Fig. 4B

lipid glycans
of 3h schistosomula

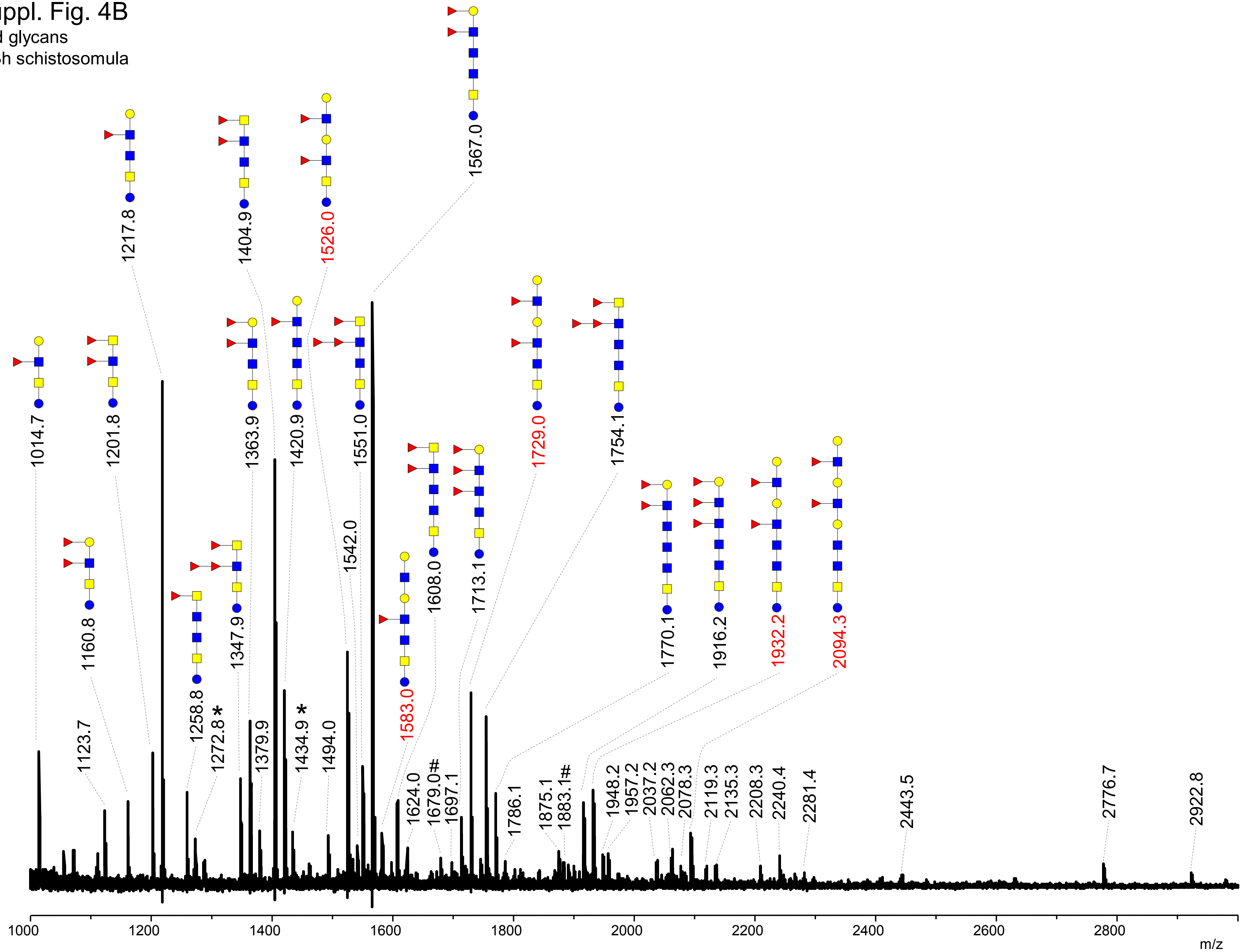

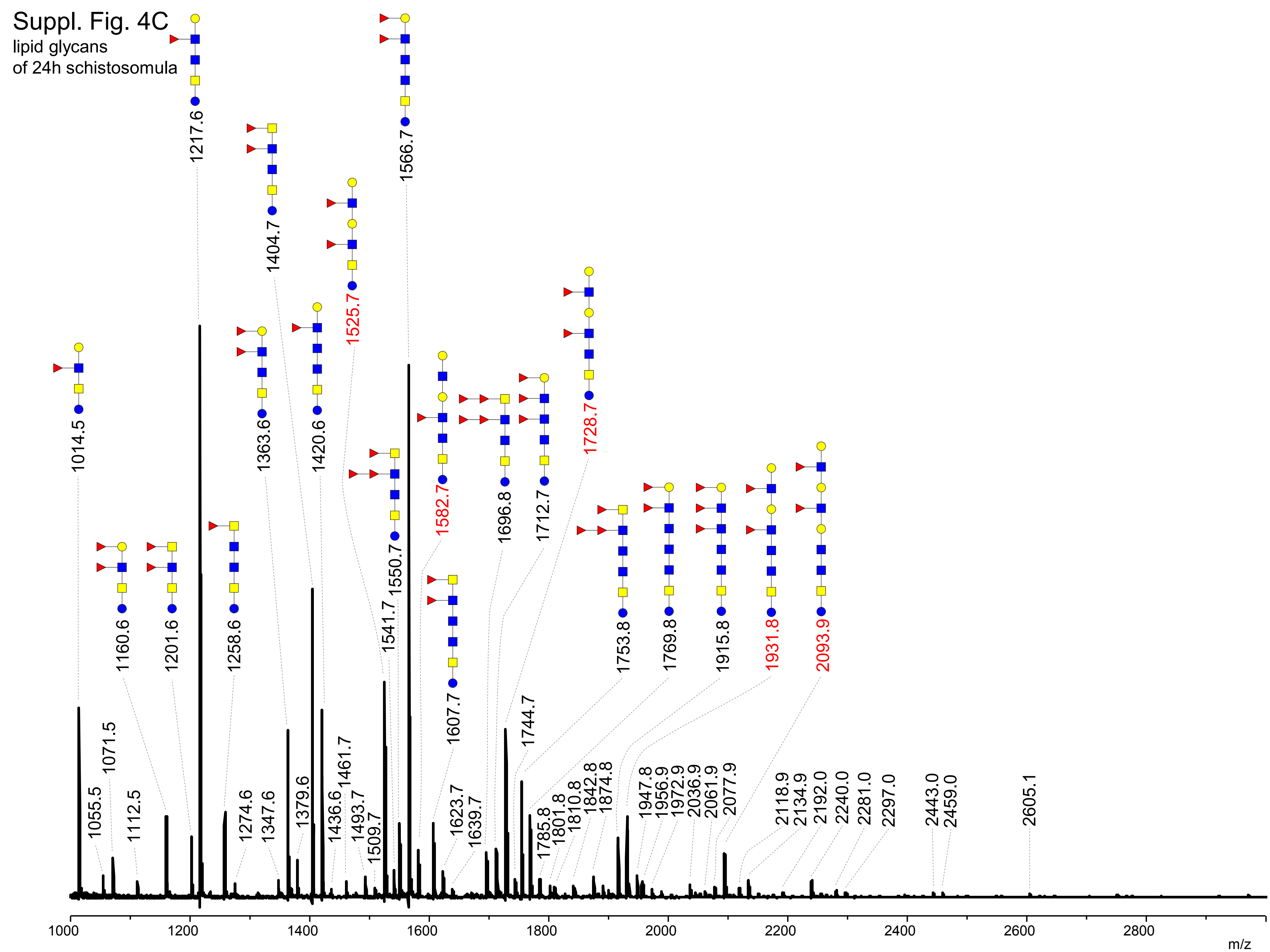
Suppl. Fig. 4C
lipid glycans
of 24h schistosomula
1014.5
1055.5
1071.5
1112.5
1160.6
1201.6
1217.6
1258.6
1274.6
1347.6
1363.6
1379.6
1404.7
1420.6
1436.6
1461.7
1493.7
1509.7
1525.7
1541.7
1550.7
1566.7
1582.7
1607.7
1623.7
1639.7
1696.8
1712.7
1728.7
1744.7
1753.8
1769.8
1785.8
1801.8
1810.8
1842.8
1874.8
1915.8
1931.8
1947.8
1956.9
1972.9
2036.9
2061.9
2077.9
2093.9
2118.9
2134.9
2192.0
2240.0
2281.0
2297.0
2443.0
2459.0
2605.1
1000
1200
1400
1600
1800
2000
2200
2400
2600
2800
m/z

Suppl. Fig. 4D
lipid glycans
of 48h schistosomula

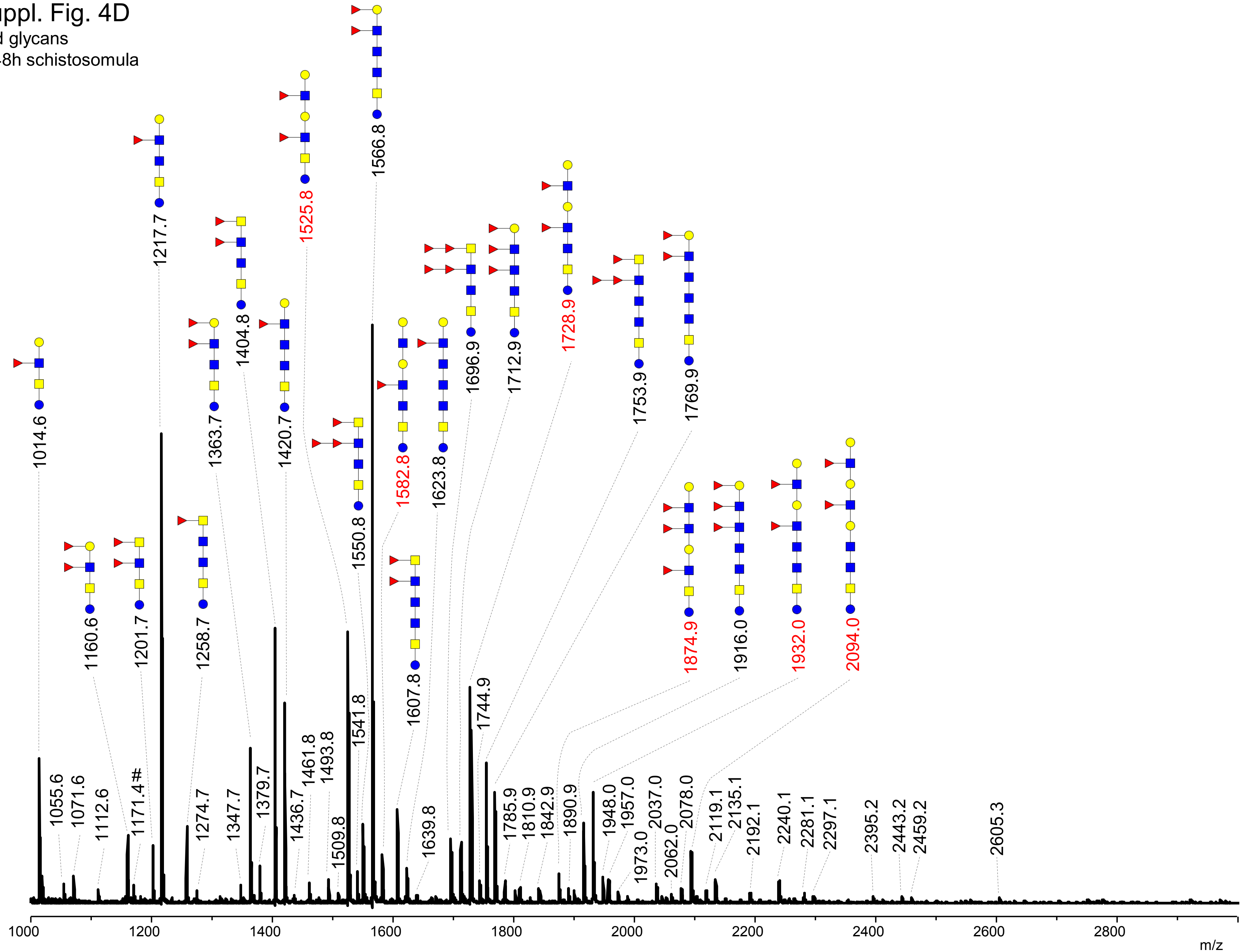

Suppl. Fig. 4E
lipid glycans
of 3 days schistosomula

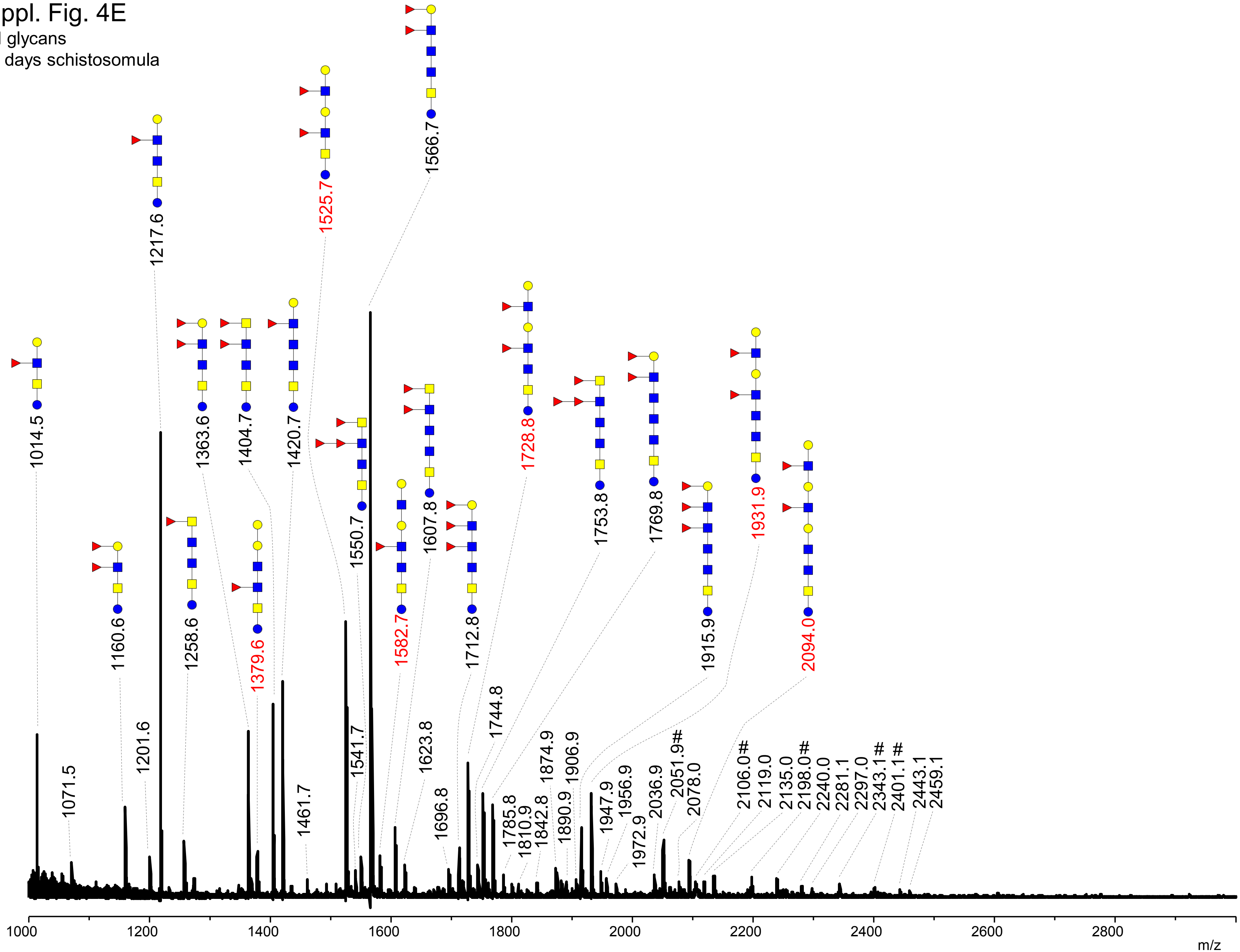

Suppl. Fig. 4F
lipid glycans
of 9 days schistosomula

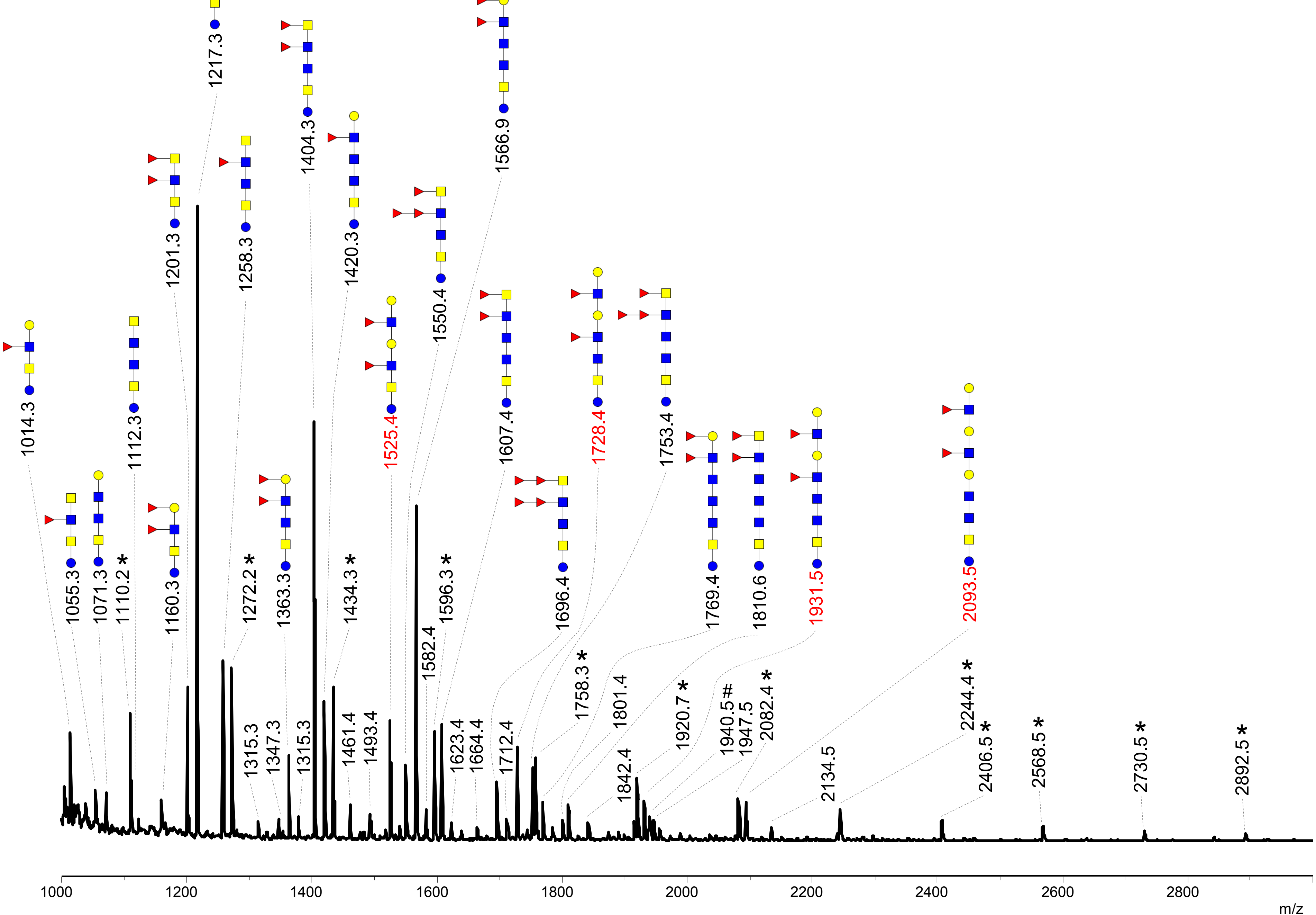

Suppl. Fig. 4G
lipid glycans
of adult worms

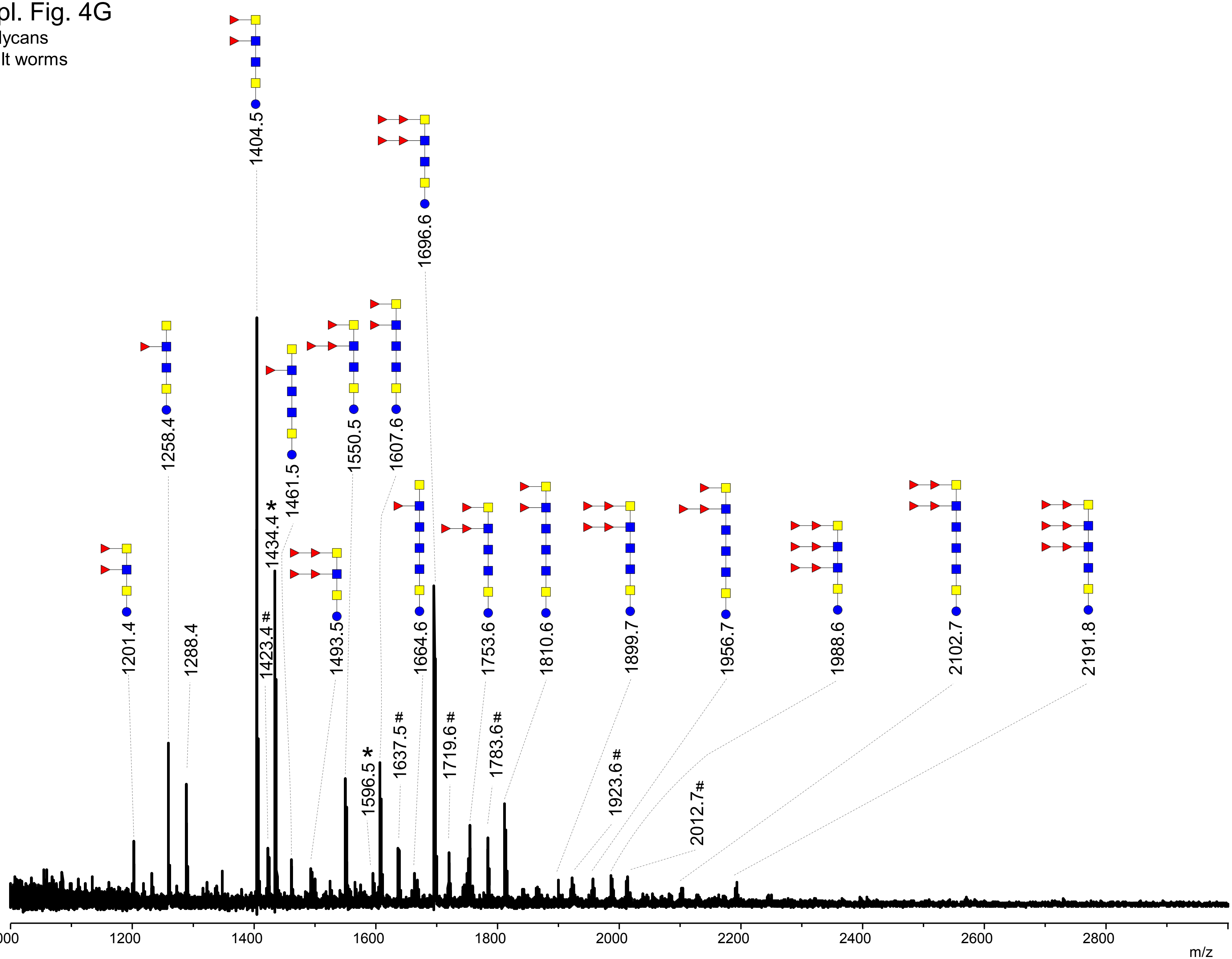

Suppl. Fig. 4H
lipid glycans
of immature eggs

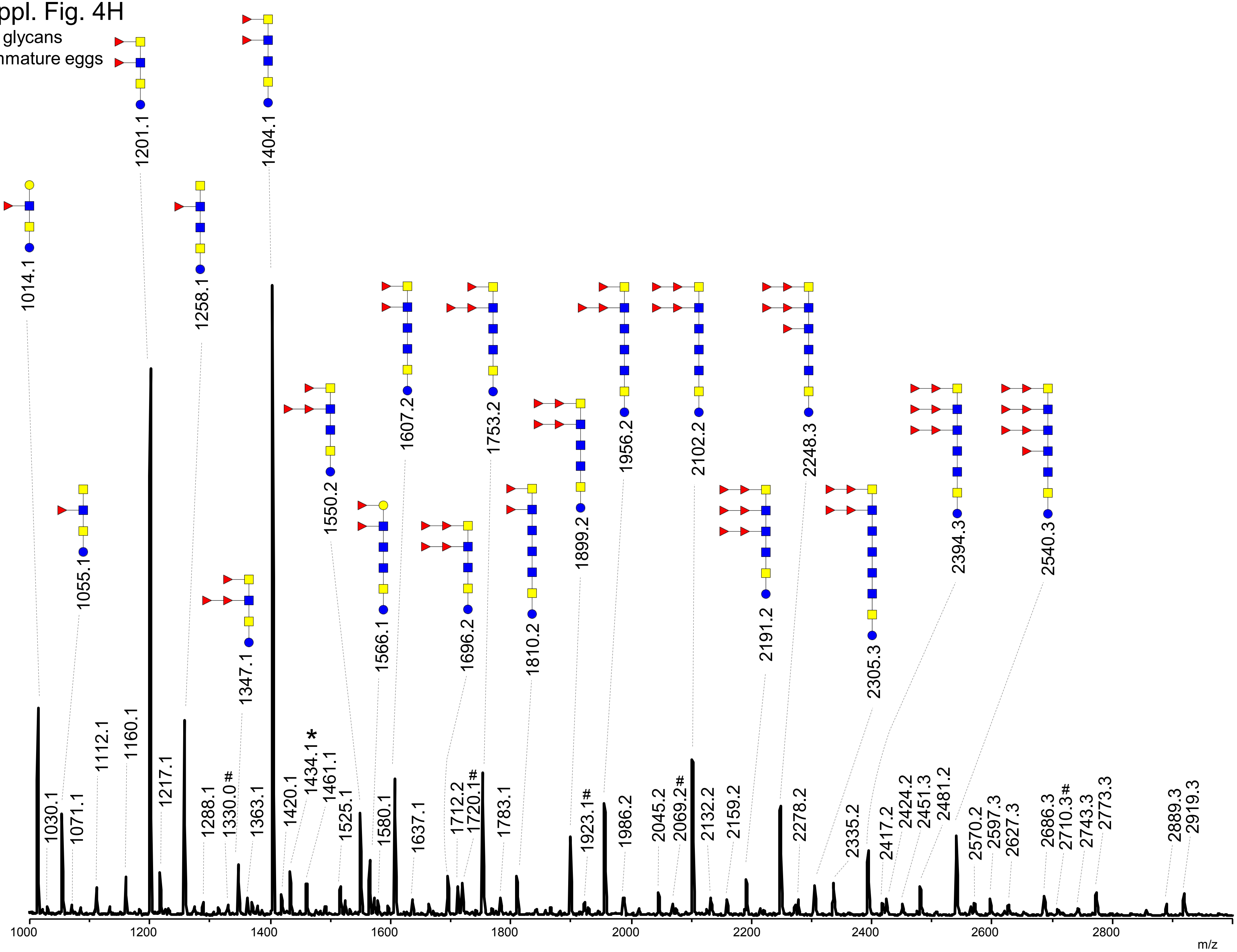

Suppl. Fig. 4I
lipid glycans
of mature eggs

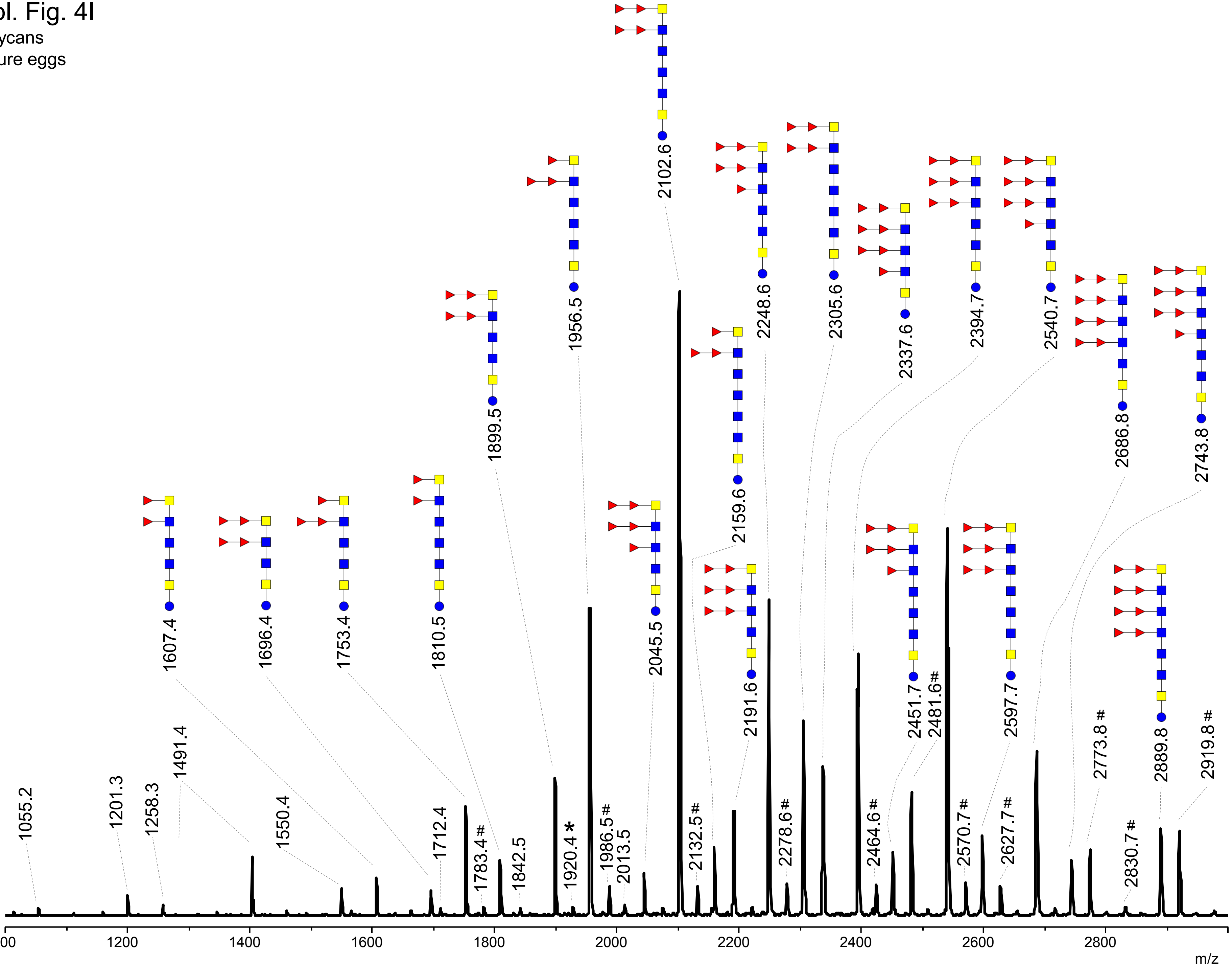

Suppl. Fig. 4J
lipid glycans
of miracidia

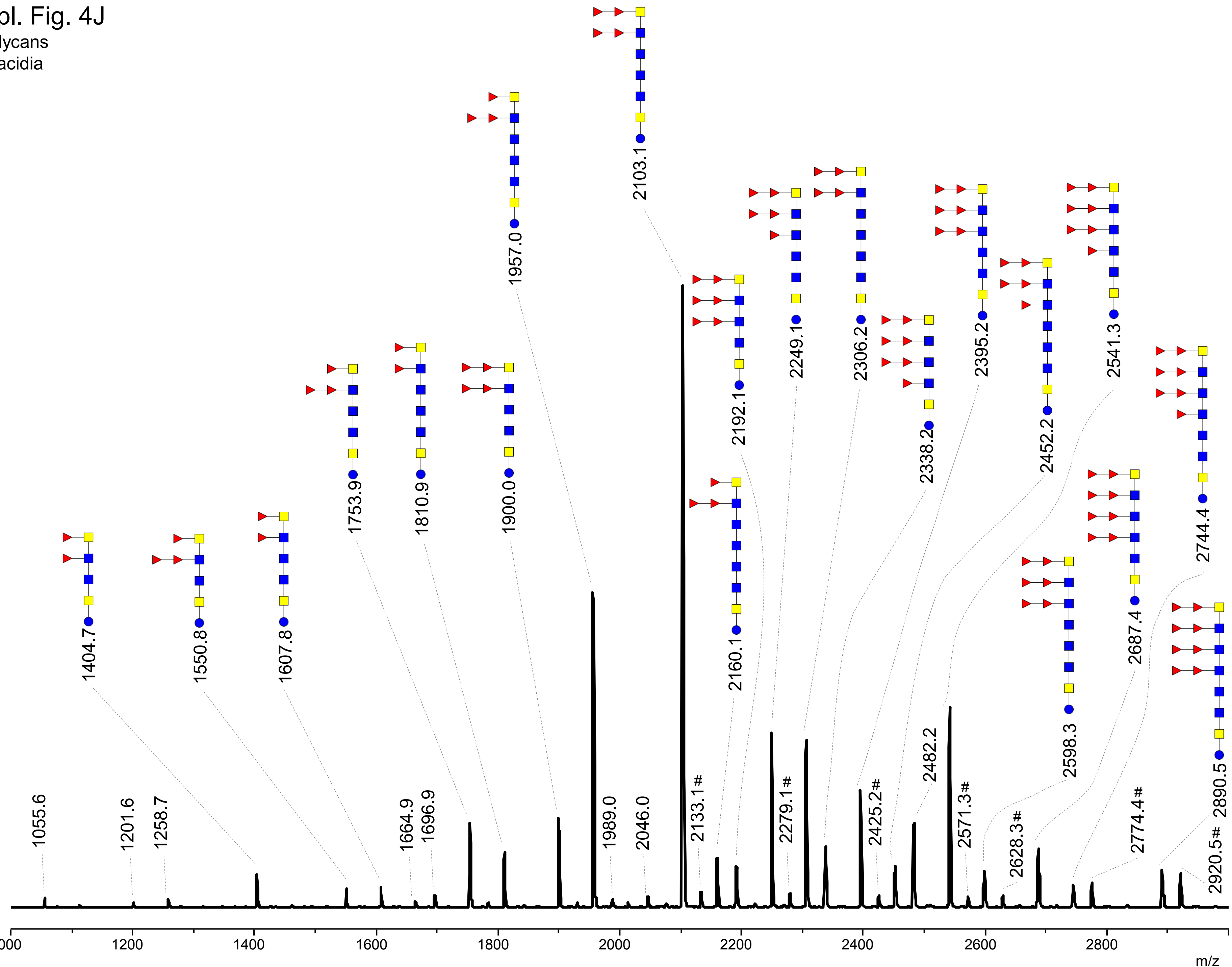

Supplement: Supplemental Data [file supp_M115.048280_mcp.M115.048280-5.pdf]
