# Supplementary material for: Glycomic Analysis of Life Stages of the Human Parasite Schistosoma mansoni Reveals Developmental Expression Profiles of Functional and Antigenic Glycan Motifs
Source: Mol Cell Proteomics. 2015 Apr 16;14(7):1750–69. doi: 10.1074/mcp.M115.048280 (PMC4587318; doi:10.1074/mcp.M115.048280)

Suppl. Fig. 5A

MALDI-TOF-MS/MS analysis of lipid glycan at m/z 1525[M-H⁺] F2H3N3 found in cercariae

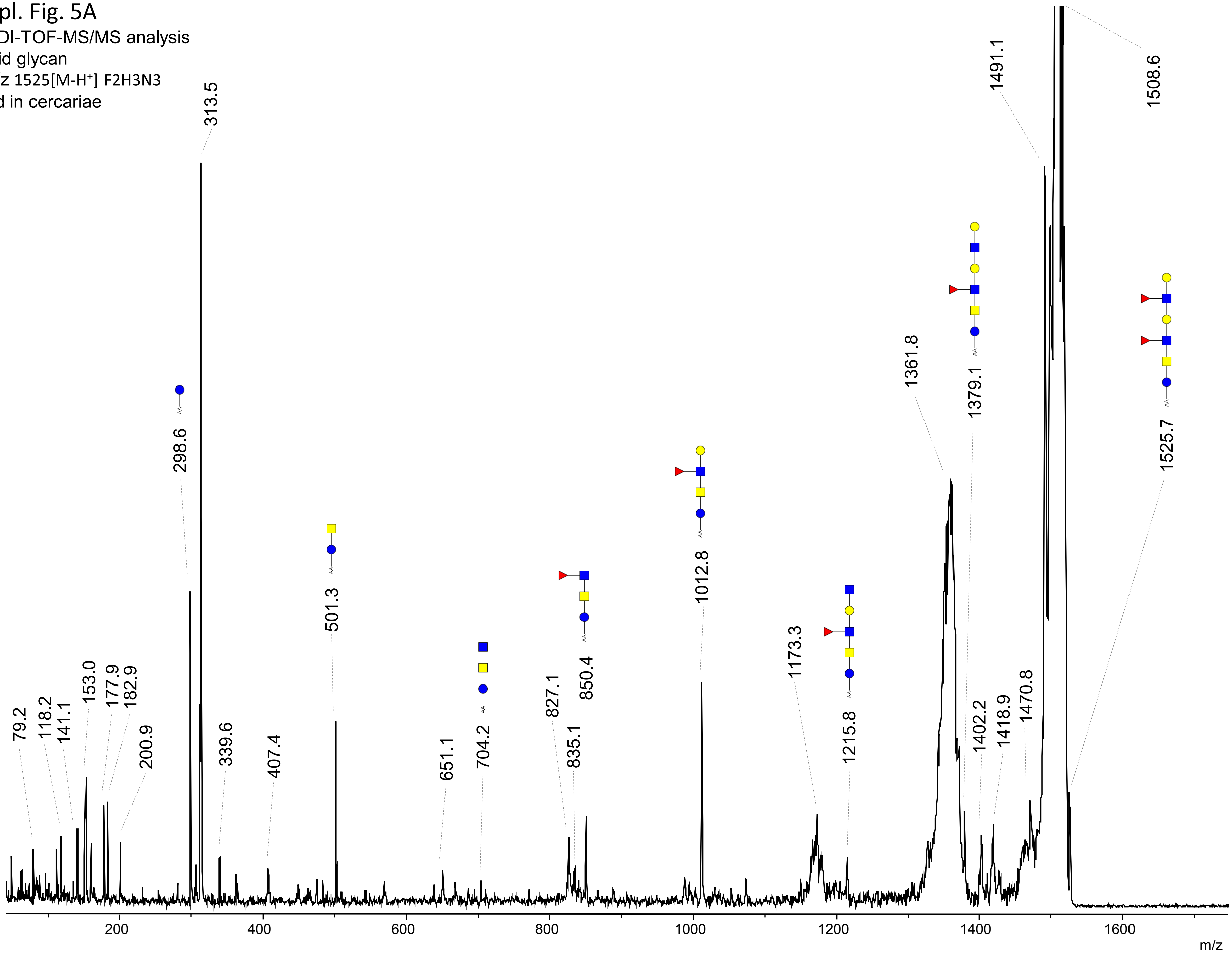

Suppl. Fig. 5B

MALDI-TOF-MS/MS analysis of lipid glycan at m/z 1541[M-H$^+$] F1H4N3 found in cercariae

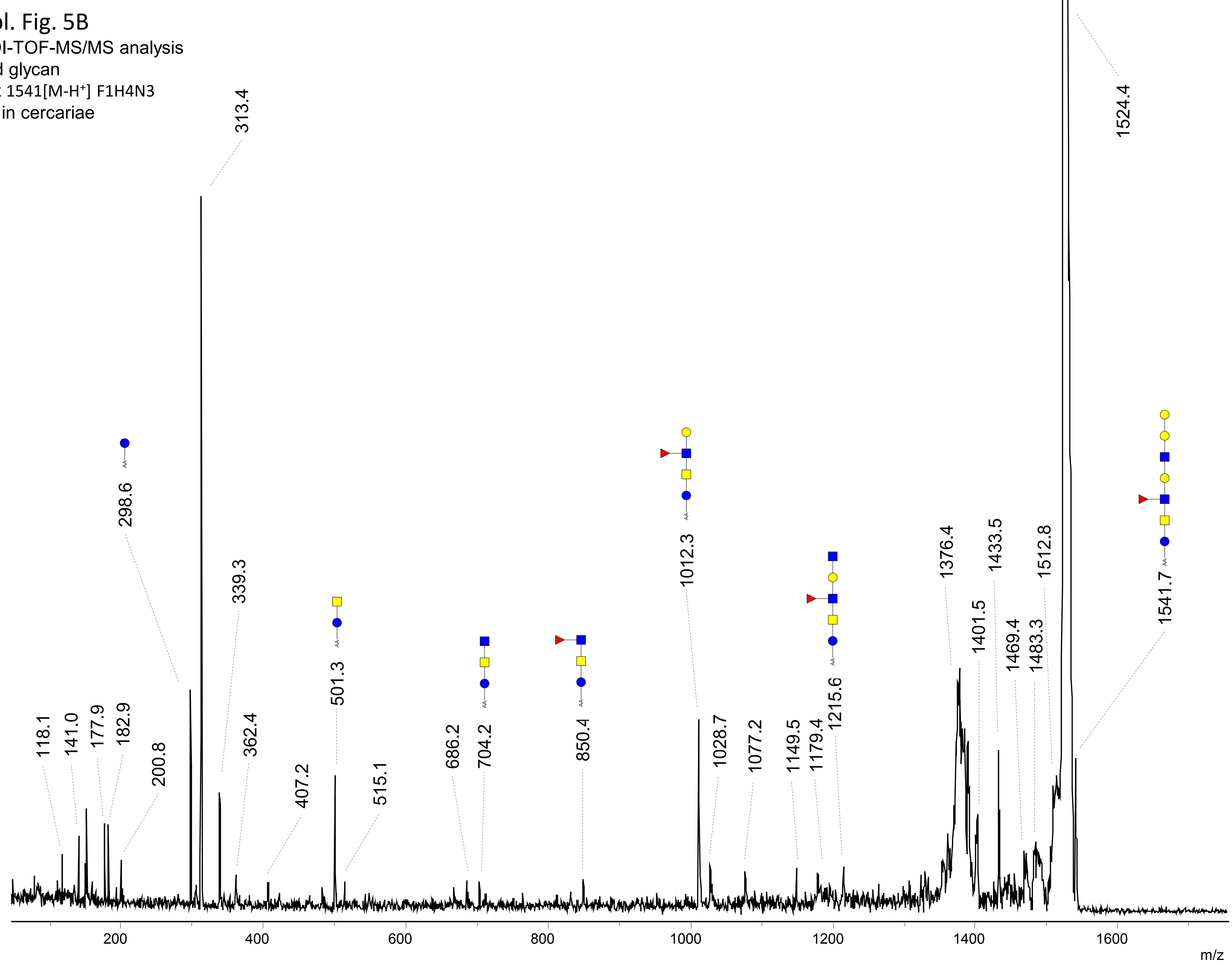

Suppl. Fig. 5C

MALDI-TOF-MS/MS analysis
of lipid glycan
at m/z 1728[M-H⁺] F2H3N4
found in cercariae

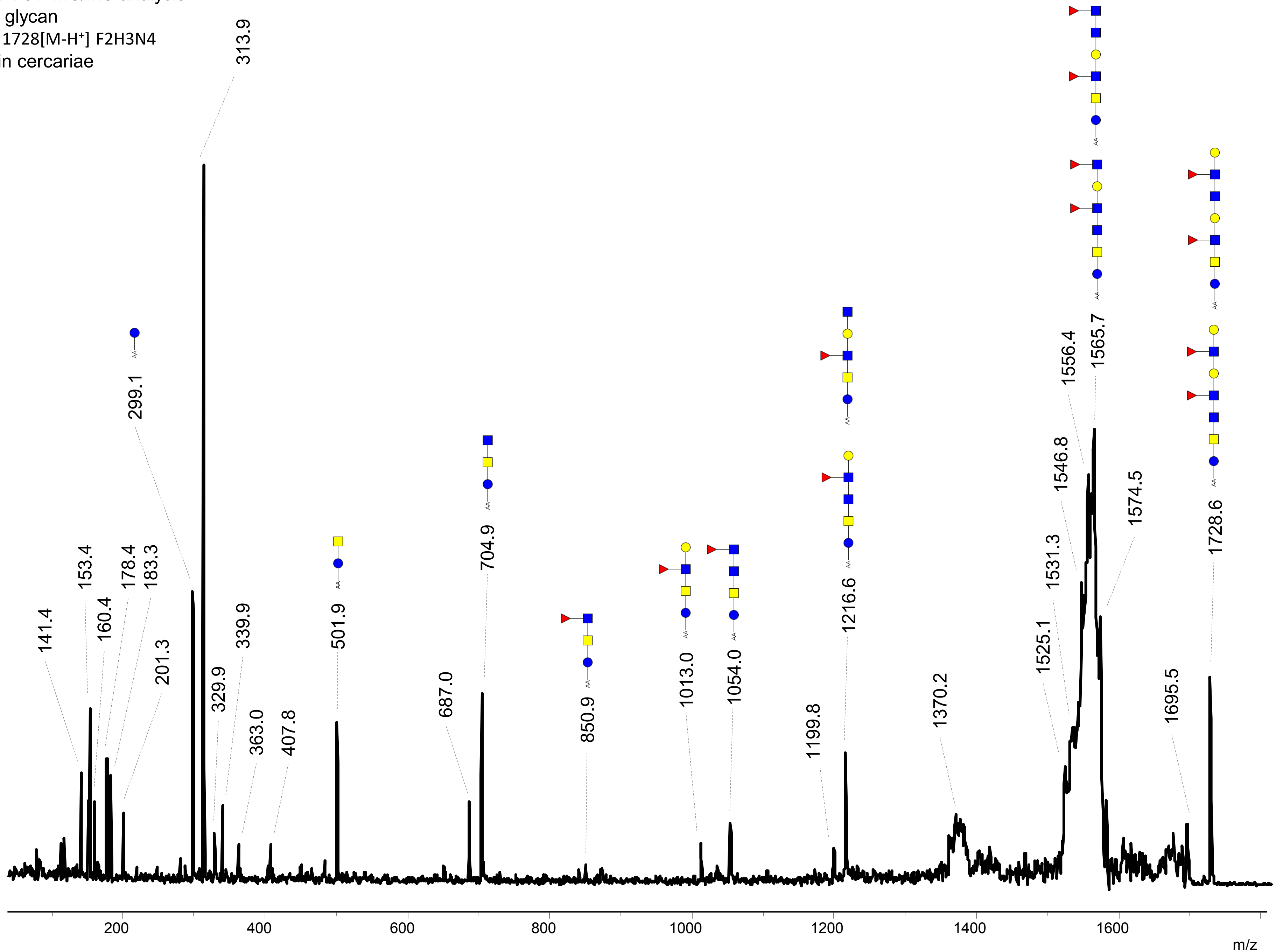

Supplement: Supplemental Data [file supp_M115.048280_mcp.M115.048280-6.pdf]
