# Supplementary material for: Glycomic Analysis of Life Stages of the Human Parasite Schistosoma mansoni Reveals Developmental Expression Profiles of Functional and Antigenic Glycan Motifs
Source: Mol Cell Proteomics. 2015 Apr 16;14(7):1750–69. doi: 10.1074/mcp.M115.048280 (PMC4587318; doi:10.1074/mcp.M115.048280)
Supplement: Supplemental Data [file supp_M115.048280_mcp.M115.048280-7.pdf]

Supplemental Table 1. Overview of exoglycosidase digestions of *S.mansoni* 3 days schistosomula PNGase F-sensitive N-glycans and structures deduced**.**

Registered masses[1]	Putative Composition[2]	Jackbean N-acetylhexosaminidase treatment[3]	Jackbean beta-Galactosidase treatment[3]	Bovine Kidney Fucosidase treatment[3]	Xanthomonas Manihotis Fucosidase treatment[3]	Most probable structural characteristics[4]
1176.6	F1H3N2	-	-	1030 (-1F)	-	core fucosylation
1308.6	X1F1H3N2	-	-	1162 (-1F)	-	core fucosylation; core xylosylation
1354.6	H5N2	-	-	-	-	oligomannose
1516.7	H6N2	-	-	-	-	oligomannose
1541.7	F1H4N3	-	1379 (-1H)	1395 (-1F)	-	1 LacNAc; core fucosylation
1582.8	F1H3N4	1176 (-2N)	-	1436 (-1F)	-	2 single HexNAc or 1 LDN; core fucosylation
1678.7	H7N2	-	-	-	-	oligomannose
1687.8	F2H4N3	-	-	1395 (-2F)	1541 (-1F)	1 Lewis X; core fucosylation
1744.8	F1H4N4	1541 (-1N)	1582 (-1H)	1598 (-1F)	-	1 single HexNAc; 1 LacNAc; core fucosylation
1760.8	H5N4	-	1436 (-2H)	-	-	2 LacNAc
1819.9	X1F2H4N3	-	-	1527 (-2F)	1673 (-1F)	1 Lewis X; core fucosylation; core xylosylation
1840.8	H8N2	-	-	-	-	oligomannose
1906.9	F1H5N4	-	1582 (-2H)	1760 (-1F)	-	2 LacNAc; core fucosylation
2002.9	H9N2	-	-	-	-	oligomannose
2038.6	X1F1H5N4	-	1876 (-1H) / 1714 (-2H)	1892 (-1F)	-	2 LacNAc; core fucosyaltion; core xylosylation
2053.0	F2H5N4	-	1890 (-1H)	1760 (-2F)	1906 (-1F)	1 LacNAc; 1Lewis X; core fucosylation
2165.0	H10N2	-	-	-	-	oligomannose
2185.0	X1F2H5N4	-	2022 (-1H)	1892 (-2F)	2038 (-1F)	1 LacNAc; 1Lewis X; core fucosylation; core xylosylation
2199.1	F3H5N4	-	-	1760 (-3F)	1906 (-2F)	2 Lewis X; core fucosylation
2231.1	F1H7N4	-	1906 (-2H)	2085 (-1F)	-	2 LacNAc; core fucosylation; hybrid type
2272.1	F1H6N5	-	2109 (-1H) / 1947 (-2H) / 1785 (-3H)	2125 (-1F)	-	3 LacNAc; core fucosylation
2331.1	X1F3H5N4	-	-	1892 (-3F)	2038 (-2F)	2 Lewis X; core fucosylation; core xylosylation

[1] Registered mass in the undigested spectrum
[2] X, Xylose; F, Fucose; H, Hexose; N, N-acetylhexosamine
[3] Resulting masses after digestion of the original mass with the different exoglycosidases. Changes in monosaccharides are indicated in parenthesis. -, no shift in mass after exoglycosidase digestion.
[4] Core xylosylation was assigned on the basis of composition.

Supplemental Table 2. Overview of exoglycosidase digestions of *S.mansoni* 6 weeks worms PNGase F-sensitive N-glycans and structures deduced**.**

Registered masses[1]	Putative Composition[2]	Jackbean N-acetylhexosaminidase treatment[3]	Jackbean beta-Galactosidase treatment[3]	Bovine Kidney Fucosidase treatment[3]	Xanthomonas Manihotis Fucosidase treatment[3]	Most probable structural characteristics[4]
1176.5	F1H3N2	-	-	1030 (-1F)	-	core fucosylation
1354.5	H5N2	-	-	-	-	oligomannose
1516.6	H6N2	-	-	-	-	oligomannose
1541.6	F1H4N3	-	1379 (-1H)	1395 (-1F)	-	1 LacNAc; core fucosylation
1582.7	F1H3N4	1379 (-1N) / 1176 (-2N)	-	1436 (-1F)	-	2 single HexNAc or 1 LDN; core fucosylation
1678.6	H7N2	-	-	-	-	oligomannose
1785.7	F1H3N5	1379 (-2N) / 1176 (-3N)	-	1639 (-1F)	-	1LDN-HexNAc or 1 LDN and 1 single HexNAc; core fucosylation
1840.7	H8N2	-	-	-	-	oligomannose
1906.8	F1H5N4	-	1744 (-1H) / 1582 (-2H)	1760 (-1F)	-	2 LacNAc; core fucosylation
1947.8	F1H4N5	1744 (-1N) / 1541 (-2N)	1785 (-1H)	1801 (-1F)	-	2 single HexNAc or 1 LDN; 1 LacNAc; core fucosylation
1988.8	F1H3N6	1785 (-1N) / 1379 (-3N) / 1176 (-4N)	-	1842 (-1F)	-	2 LDN or 1 LDN-LDN; core fucosylation
2002.7	H9N2	-	-	-	-	oligomannose
2052.8	F2H5N4	-	1890 (-1H)	1906 (-1F) / 1760 (-2F)	1906 (-1F)	1 LacNAc; 1Lewis X; core fucosylation
2109.9	F1H5N5	1906 (-1N)	1947 (-1H) / 1785 (-2H)	1963 (-1F)	-	1 single HexNAc; 2 LacNAc; core fucosylation
2134.9	F2H3N6	1931 (-1N) / 1728 (-2N) / 1525 (-3N)	-	1988 (-1F) / 1842 (-2F)	1988 (-1F)	1 LDN-F and 1 LDN or 1 LDN-(LDN-F); core fucosylation
2164.8	H10N2	-	-	-	-	oligomannose
2198.9	F3H5N4	-	-	1906 (-2F) / 1760 (-3F)	2052 (-1F) / 1906 (-2F)	2 Lewis X; core fucosylation
2271.9	F1H6N5	-	1947 (-2H) / 1785 (-3H)	2125 (-1F)	-	3 LacNAc; core fucosylation
2281.0	F3H3N6	2077 (-1N) / 1874 (-2N)	-	2134 (-1F) / 1988 (-2F) / 1842 (-3F)	2134 (-1F) / 1988 (-2F)	2 LDN-F or 1 LDN-(F-LND-F); core fucosylation
2395.0	F1H3N8	1785 (-3N) / 1379 (-5N) / 1176 (-6N)	-	2248 (-1F)	-	1 LDN-LDN and 1 LDN; core fucosylation

[1] Registered mass in the undigested spectrum
[2] X, Xylose; F, Fucose; H, Hexose; N, N-acetylhexosamine
[3] Resulting masses after digestion of the original mass with the different exoglycosidases. Changes in monosaccharides are indicated in parenthesis. -, no shift in mass after exoglycosidase digestion.
[4] Core xylosylation was assigned on the basis of composition.

Supplemental Table 3. Overview of exoglycosidase digestions of *S.mansoni* mature eggs PNGase F-sensitive N-glycans and structures deduced**.**

Registered masses[1]	Putative Composition [2]	Jackbean N-acetylhexosaminidase treatment[3]	Jackbean beta-Galactosidase treatment[3]	Bovine Kidney Fucosidase treatment[3]	Xanthomonas Manihotis Fucosidase treatment[3]	Most probable structural characteristics [4]
1308.4	X1F1H3N2	-	-	1162 (-1F)	-	core fucosylation; core xylosylation
1354.4	H5N2	-	-	-	-	oligomannose
1511.5	X1F1H3N3	1308 (-1N)	-	1365 (-1F)	-	1 single HexNAc; core fucosylation; core xylosylation
1516.5	H6N2	-	-	-	-	oligomannose
1678.5	H7N2	-	-	-	-	oligomannose
1840.6	H8N2	-	-	-	-	oligomannose
1860.6	X1F2H3N4	1657 (-1N)	-	1714 (-1F) / 1568 (-2F)	1714 (-1F)	1 LDN-F or 1 single HexNAc and 1 fucosylated HexNAc; core xylosylation; core fucosylation
1906.6	F1H5N4	-	1744 (-1H) / 1582 (-2H)	1760 (-1F)	1760 (-1F)	2 LacNAc; core fucosylation
1947.6	F1H4N5	1744 (-1N) / 1541 (-2N)	1785 (-1H)	1801 (-1F)	1801 (-1F)	1 LDN of 2 single HexNAc; 1 LacNAc; core fucosylation
2002.6	H9N2	-	-	-	-	oligomannose
2006.7	X1F3H3N4	1803 (-1N)	-	1860 (-1F) / 1714 (-2F) / 1568 (-3F)	1860 (-1F) / 1714 (-2F)	1 LDN-DF or 1 single HexNAc and 1 double fucosylated HexNAc; core fucosylation; core xylosylation
2052.7	F2H5N4	-	1890 (-1H)	1760 (-2F)	1906 (-1F) / 1760 (-2F)	1 LacNAc; 1 Lewis X; core fucosylation
2093.7	F2H4N5	1890 (-1N)	1931 (-1H)	1947 (-1F) / 1801 (-2F)	1947 (-1F) / 1801 (-2F)	1 LDN-F or 1 single HexNAc and 1 single fucosylated HexNAc; 1 LacNAc; core fucosylation
2152.7	X1F4H3N4	-	-	1860 (-2F) / 1714 (-3F) / 1568 (-4F)	2006 (-1F)	1 F-LDN-DF or 1 single fucosylated HexNAc and 1 double fucosylated HexNAc; core fucosylation; core xylosylation
2164.7	H10N2	-	-	-	-	oligomannose
2168.7	X1F3H4N4	1965 (-1N)	2006 (-1H)	2022 (-1F)	2022 (-1F) / 1876 (-2F)	1 Lewis X and 1 double fucosylated HexNAc; core xylosylation
2198.7	F3H5N4	-	-	2052 (-1F) / 1906 (-2F) / 1760 (-3F)	2052 (-1F) / 1906 (-2F)	2 Lewis X; core fucosylation
2298.8	X1F5H3N4	-	-	2152 (-1F) / 1860 (-3F) / 1714 (-4F) / 1568 (-5F)	-	1 DF-LDN-DF or 2 double fucosylated HexNAc;<br>core fucosylated; core xylosylation
2314.8	X1F4H4N4	-	-	2022 (-2F)	2168 (-1F)	1 Lewis X and 1 double fucosylated HexNAc; core fucosylation; core xylosylation
2330.8	X1F3H5N4	-	-	2184 (-1F) / 2038 (-2F)	2184 (-1F)	2 Lewis X; core fucosylation; core xylosylation
2460.8	X1F5H4N4	-	-	2314 (-1F) / 2022 (-3F)	2314 (-1F)	1 Lewis X and 1 triple fucosylated HexNAc; core fucosylation; core xylosylation
2526.8	X1F1H3N8	1714 (-4N) / 1511 (-5N) / 1308 (-6N)	-	2379 (-1F)	-	3 LDN; core fucosylation; core xylosylation

[1] Registered mass in the undigested spectrum
[2] X, Xylose; F, Fucose; H, Hexose; N, N-acetylhexosamine
[3] Resulting masses after digestion of the original mass with the different exoglycosidases. Changes in monosaccharides are indicated in parenthesis. -, no shift in mass after exoglycosidase digestion.
[4] Core xylosylation was assigned on the basis of composition.
