# Supplementary material for: Glycomic Analysis of Life Stages of the Human Parasite Schistosoma mansoni Reveals Developmental Expression Profiles of Functional and Antigenic Glycan Motifs
Source: Mol Cell Proteomics. 2015 Apr 16;14(7):1750–69. doi: 10.1074/mcp.M115.048280 (PMC4587318; doi:10.1074/mcp.M115.048280)
Supplement: Supplemental Data [file supp_M115.048280_mcp.M115.048280-8.pdf]

Supplemental Table 4. Relative abundance and structural characteristics of N-glycans during worm development

Registered masses[1]	Putative Composition[2]	Life stages[3]										Glycan type[4]				Core modifica-tions[4]		Antennae motifs[5]		
		Cercariae	3 hr somules	24 hr somules	3 days somules	6 days somules	2 wks worms	3 wks worms	4 wks worms	5 wks worms	6 wks worms	Complex type	Paucimannosidic	Oligomannosidic	Hybrid Type	Core xylosylation	Core fucosylation	LeX	LDN	Fucosylated LDN
1014.5	F1H2N2	-	-	-	0.3%	0.4%	-	0.4%	0.5%	0.4%	0.3%		v				v	0	0	0
1030.4	H3N2	-	0.1%	-	0.3%	0.4%	-	0.5%	0.5%	0.5%	0.3%		v					0	0	0
1146.5	X1F1H2N2	-	-	0.3%	0.4%	-	-	-	-	-	-		v			v	v	0	0	0
1176.5	F1H3N2	1.6%	0.4%	0.8%	1.8%	1.5%	2.1%	2.8%	3.8%	2.9%	1.7%		v				v	0	0	0
1192.6	H4N2	-	-	-	0.3%	0.5%	1.0%	0.8%	0.7%	0.5%	0.4%		v					0	0	0
1233.7	H3N3	-	-	-	-	-	-	0.2%	0.2%	0.2%	-	v						0	0	0
1308.5	X1F1H3N2	3.0%	1.2%	1.7%	1.6%	0.7%	-	0.2%	0.2%	0.2%	-		v			v	v	0	0	0
1338.7	F1H4N2	-	-	-	-	-	-	0.3%	-	-	-		v				v	0	0	0
1354.5	H5N2	0.4%	0.6%	0.9%	3.3%	3.6%	4.0%	3.0%	1.8%	1.6%	1.7%			v				0	0	0
1379.5	F1H3N3	0,3%	0.2%	0.3%	0.6%	0.8%	1.4%	1.3%	1.4%	1.2%	0.9%	v					v	0	0	0
1395.7	H4N3	-	-	-	-	0.3%	0.7%	0.2%	0.3%	0.4%	0.4%	v						0	0	0
1436.8	H3N4	-	-	-	-	-	-	0.7%	1.2%	1.2%	1.0%	v						0	1	0
1511.5	X1F1H3N3	0,7%	0.4%	0.6%	0.6%	0.3%	-	-	-	-	-	v				v	v	0	0	0
1516.5	H6N2	1.0%	1.8%	2.1%	5.6%	9.8%	10.4%	11.5%	8.7%	8.6%	8.2%			v				0	0	0
1525.7	F2H3N3	-	0.1%	-	0.2%	-	-	-	0.2%	0.2%	-	v					v	0	0	0
1527.6	X1H4N3	-	0.1%	0.3%	-	-	-	-	-	-	-	v				v		0	0	0
1541.6	F1H4N3	0.8%	0.6%	0.7%	1.2%	1.1%	1.7%	0.9%	1.5%	1.9%	1.9%	v					v	0	0	0
1557.7	H5N3	-	-	-	0.3%	0.3%	0.7%	0.2%	0.2%	0.3%	0.5%				v			0	0	0
1582.6	F1H3N4	0.4%	0.7%	0.6%	1.5%	1.9%	2.9%	4.1%	7.7%	8.4%	7.5%	v					v	0	1	0
1598.6	H4N4	-	-	-	-	-	-	-	-	-	0.5%	v						0	0	0
1640.0	H3N5	-	-	-	-	-	-	-	0.2%	0.2%	0.3%	v						0	1	0
1657.6	X1F2H3N3	0.4%	0.2%	0.3%	-	-	-	-	-	-	-	v				v	v	0	0	0
1662.8	F1H6N2	-	-	-	-	0.3%	-	0.2%	0.2%	0.2%	0.3%			v			v	0	0	0
1673.6	X1F1H4N3	2.6%	1.8%	1.9%	1.0%	0.4%	-	-	0.2%	0.2%	0.3%	v				v	v	0	0	0
1678.5	H7N2	1.9%	2.1%	2.6%	5.8%	9.1%	7.6%	7.1%	5.8%	5.5%	5.8%			v				0	0	0
1687.6	F2H4N3	6.2%	2.4%	2.0%	2.4%	0.8%	1.0%	0.7%	0.6%	1.0%	0.9%	v					v	1	0	0
1703.8	F1H5N3	-	-	-	0.4%	0.4%	-	0.3%	0.3%	0.5%	0.6%				v		v	0	0	0
1714.6	X1F1H3N4	0.6%	0.6%	0.9%	0.9%	0.5%	-	-	0.2%	0.2%	-	v				v	v	0	1	0
1719.7	H6N3	-	0.2%	-	0.8%	0.5%	0.7%	0.4%	0.2%	-	0.3%				v			0	0	0
1728.6	F2H3N4	0.4%	0.2%	0.3%	0.4%	0.3%	-	0.7%	1.0%	1.2%	1.0%	v					v	0	0	1
1730.7	X1H4N4	-	0.2%	0.3%	-	-	-	-	-	-	-	v				v		0	0	0
1744.6	F1H4N4	0.3%	0.4%	0.4%	1.6%	1.0%	1.5%	1.1%	0.8%	1.0%	1.1%	v					v	0	0	0
1760.7	H5N4	-	1.8%	1.3%	1.2%	1.9%	2.6%	0.8%	0.9%	0.8%	0.9%	v						0	0	0
1785.7	F1H3N5	-	0.1%	0.3%	0.6%	0.7%	1.3%	1.0%	1.5%	1.7%	1.8%	v					v	0	1	0
1801.9	H4N5	-	-	-	-	0.3%	-	-	0.2%	0.3%	0.4%	v						0	1	0
1819.6	X1F2H4N3	21.2%	8.9%	7.8%	3.4%	0.3%	-	-	-	0.2%	-	v				v	v	1	0	0

1835.8	X1F1H5N3	-	0.2%	-	-	-	-	-	-	-	-				v	v	v	0	0	0
1840.6	H8N2	1.5%	2.1%	2.8%	6.9%	13.3%	14.3%	13.2%	9.9%	8.6%	8.0%			v				0	0	0
1850.0	F2H5N3	-	-	-	-	-	-	0.3%	0.2%	0.3%	-				v		v	0	0	0
1860.6	X1F2H3N4	0.7%	0.6%	0.7%	0.8%	0.4%	-	-	-	0.2%	0.3%	v				v	v	0	0	1
1866.8	F1H6N3	-	0.3%	-	0.4%	0.4%	-	0.3%	-	-	-				v		v	0	0	0
1875.1	F3H3N4	-	-	-	-	-	-	-	0.2%	0.2%	-	v					v	0	0	1
1876.6	X1F1H4N4	0.9%	1.5%	1.5%	0.6%	-	-	-	-	-	-	v				v	v	0	0	0
1881.9	H7N3	-	-	-	0.3%	-	-	-	-	-	-				v			0	0	0
1890.6	F2H4N4	0.8%	0.4%	0.5%	0.5%	0.5%	1.0%	0.8%	0.4%	0.4%	0.4%	v					v	1	0	0
1892.8	X1H5N4	-	1.2%	1.3%	-	-	-	-	-	-	-	v				v		0	0	0
1906.6	F1H5N4	1.5%	2.9%	3.0%	5.9%	7.0%	4.6%	2.4%	2.4%	2.5%	2.8%	v					v	0	0	0
1917.8	X1F1H3N5	-	0.2%	-	0.3%	-	-	-	-	-	-	v				v	v	0	1	0
1932.2	F2H3N5	-	-	-	-	-	-	-	0.2%	0.2%	0.3%	v					v	0	1	1
1947.8	F1H4N5	-	0.3%	0.4%	1.0%	1.2%	1.2%	0.9%	1.3%	1.9%	2.9%	v					v	0	1	0
1988.6	F1H3N6	0.4%	0.3%	0.4%	0.7%	1.2%	2.2%	3.3%	7.9%	9.0%	10.0%	v					v	0	2	0
2002.6	H9N2	3.9%	5.0%	6.6%	14.7$	22.8%	21.2%	24.8%	20.3%	17.1%	15.7%			v				0	0	0
2006.6	X1F3H3N4	0.9%	-	-	-	-	-	-	-	-	-	v				v	v	0	0	1
2012.0	F2H6N3	-	-	-	-	-	-	0.3%	0.3%	-	-				v		v	1	0	0
2022.7	X1F2H4N4	1.4%	0.9%	1.1%	0.5%	-	-	-	-	-	-	v				v	v	1	0	0
2036.8	F3H4N4	-	0.2%	0.4%	-	-	-	-	-	-	-	v					v	1	0	0
2038.7	X1F1H5N4	7.9%	13.2%	10.9%	2.2%	0.4%	-	-	-	0.2%	-	v				v	v	0	0	0
2052.7	F2H5N4	3.4%	3.5%	3.8%	4.7%	3.0%	2.5%	1.4%	1.0%	1.2%	1.3%	v					v	1	0	0
2068.9	F1H6N4	-	0.3%	0.8%	1.2%	0.4%	-	0.4%	-	-	-				v		v	0	0	0
2079.7	X1F1H4N5	0.4%	0.6%	0.6%	0.3%	-	-	-	-	-	-	v				v	v	0	1	0
2093.8	F2H4N5	-	0.5%	0.3%	0.4%	0.4%	0.6%	0.4%	0.5%	0.7%	0.9%	v					v	1	1	0
2109.9	F1H5N5	-	0.2%	0.3%	0.6%	0.5%	1.1%	0.5%	0.4%	1.2%	2.5%	v					v	0	0	0
2120.9	X1F1H3N6	-	0.2%	0.3%	0.3%	-	-	-	-	-	-	v				v	v	0	2	0
2126.0	H6N5	-	-	-	0.3%	0.3%	0.6%	0.3%	0.3%	0.,2%	0.3%	v						0	0	0
2134.6	F2H3N6	0.5%	-	-	-	-	-	0.4%	1.0%	1.2%	1.4%	v					v	0	1	1
2151.3	F1H4N6	-	-	-	-	-	-	-	0.2%	0.2%	0.3%	v					v	0	0	0
2151.9	X1F4H3N4	-	-	-	0.3%	-	-	-	-	-	-	v				v	v	0	0	1
2164.6	H10N2	0.5%	0.4%	0.6%	1.5%	2.2%	3.2%	3.1%	3.0%	2.5%	2.2%			v				0	0	0
2168.9	X1F3H4N4	-	0.2%	0.4%	-	-	-	-	-	-	-	v				v	v	1	0	0
2182.8	F4H4N4	-	-	0.4%	-	-	-	-	-	-	-	v					v	1	0	0
2184.7	X1F2H5N4	14.4%	16.0%	15.0%	2.2%	0.4%	-	-	-	-	-	v				v	v	1	0	0
2192.2	F1H3N7	-	-	-	-	-	-	0.4%	0.4%	0.3%	0.4%	v					v	0	1	0
2198.7	F3H5N4	6.1%	6.4%	5.5%	7.4%	2.8%	2.6%	1.2%	1.2%	1.6%	1.6%	v					v	2	0	0
2209.9	X1F3H3N5	-	0.4%	-	-	-	-	-	-	-	-	v				v	v	0	0	1
2225.7	X1F2H4N5	0.7%	0.7%	0.8%	0.3%	-	-	-	-	-	-	v				v	v	1	1	0
2230.9	F1H7N4	-	0.5%	-	2.0%	0.5%	-	0.5%	-	-	-				v		v	0	0	0
2240.0	F3H4N5	-	-	-	0.3%	-	-	0.3%	0.5%	0.7%	0.7%	v					v	1	0	1
2241.9	X1F1H5N5	-	0.4%	0.4%		-	-	-	-		-	v				v	v	0	0	0
2256.1	F2H5N5	-	-	-	-	-	0.7%	0.2%	-	0.2%	0.2%	v					v	1	0	0
2271.9	F1H6N5	-	0.3%	0.5%	1.9%	1.4%	1.5%	1.0%	1.4%	2.1%	3.0%	v					v	0	0	0
2281.3	F3H3N6	-	-	-	-	-	-	0.4%	0.8%	1.3%	1.4%	v					v	0	0	2
2313.1	F1H5N6	-	-	-	0.2%	0.2%	-	-	0.2%	0.2%	0.4%	v					v	0	1	0
2327.3	H11N2	-	-	-	-	-	-	-	0.1%	-	-			v				0	0	0

2330.8	X1F3H5N4	10.5%	9.8%	9.4%	1.7%	0.3%	-	-	-	-	-	v		v	v	2	0	0
2345.0	F4H5N4	-	0.4%	0.4%	0.4%	-	-	0.2%	-	-	-		v		v	0	0	1
2346.7	X1F2H6N4	0.4%	0.5%	0.4%	-	-	-	-	-	-	-		v	v	v	1	0	0
2354.4	F1H4N7	-	-	-	-	-	-	-	0.2%	0.2%	0.2%	v			v	0	1	0
2371.8	X1F3H4N5	0.5%	0.5%	0.5%	-	-	-	-	-	-	-	v		v	v	1	0	1
2387.8	X1F2H5N5	0.4%	0.4%	0.4%	-	-	-	-	-	-	-	v		v	v	1	0	0
2394.9	F1H3N8	-	-	0.4%	0.4%	0.6%	0.7%	1.0%	1.5%	1.4%	1.3%	v			v	0	2	0
2402.2	F3H5N5	-	-	-	-	-	0.7%	0.4%	0.2%	-	-	v			v	1	0	0
2413.0	X1F3H3N6	-	0.2%	-	0.4%	0.2%	-	-	-	-	-	v		v	v	0	0	2
2418.0	F2H6N5	-	0.3%	-	0.6%	0.6%	1.0%	0.5%	0.3%	0.5%	0.6%	v			v	1	0	0
2458.8	F2H5N6	-	-	-	-	-	-	-	-	0.2%	0.2%	v			v	1	1	0
2518.1	X1F4H4N5	-	0.2%	-	-	-	-	-	-	-	-	v		v	v	1	0	1
2526.9	X1F1H3N8	-	-	0.4%	0.2%	-	-	-	-	-	-	v		v	v	0	2	0
2533.8	X1F3H5N5	0.6%	0.5%	0.5%	-	-	-	-	-	-	-	v		v	v	2	0	0
2541.4	F2H3N8	-	-	-	-	-	-	0.2%	0.3%	0.3%	0.3%	v			v	0	1	1
2558.9	X1F4H3N6	-	-	0.4%	0.2%	-	-	-	-	-	-	v			v	0	0	2
2564.2	F3H6N5	-	0.3%	-	0.3%	0.3%	0.8%	0.3%	0.2%	0.2%	0.2%	v			v	2	0	0
2573.5	F5H3N6	-	-	-	-	-	-	-	0.2%	0.2%	0.2%	v			v	0	0	2
2598.3	F1H3N9	-	-	-	-	0.2%	-	0.3%	0.2%	0.2%	-	v			v	0	1	0
2637.2	F1H7N6	-	-	-	0.2%	0.2%	-	-	0.2%	-	-	v			v	0	0	0
2679.9	X1F4H5N5	0.4%	0.4%	0.3%	-	-	-	-	-	-	-	v		v	v	2	0	0
2687.7	F3H3N8	-	-	-	-	-	-	-	0.2%	0.2%	0.3%	v			v	0	1	2
2710.3	F4H6N5	-	-	-	0.2%	-	-	0.3%	0.2%	0.2%	0.3%	v			v	2	0	0
2751.6	F4H5N6	-	-	-	-	-	-	-	0.1%	0.1%	0.2%	v			v	2	0	1
2783,5	F2H7N6	-	-	-	-	-	-	0.1%	-	-	-	v			v	1	0	0
2801.2	F1H3N10	-	-	-	0.2%	0.3%	-	0.4%	0.4%	0.3%	0.3%	v			v	0	2	0
2833.7	F4H3N8	-	-	-	-	-	-	-	0.2%	0.2%	0.3%	v			v	0	0	2
2929.7	F3H7N6	-	-	-	-	-	-	0.1%	-	-	-	v			v	2	0	0
2947.6	F2H3N10	-	-	-	-	-	-	0.1%	0.2%	0.1%	-	v			v	0	1	1
3004.7	F1H3N11	-	-	-	-	0.1%	-	0.1%	0.1%	-	-	v			v	0	1	0
3093.9	F3H3N10	-	-	-	-	-	-	-	0.1%	0.1%	-	v			v	0	2	2
3207.6	F1H3N12	-	-	-	-	0.1%	-	0.1%	0.1%	0.1%	-	v			v	0	2	0
3239.2	F4H3N10	-	-	-	-	-	-	-	-	0.1%	-	v			v	0	1	2
3386.0	F5H3N10	-	-	-	-	-	-	-	0.1%	0.1%	-	v			v	0	0	2

The structural interpretation for the different glycan compositions was made on the basis of the exoglycosidase digestions (Table 1 and Suppl. Tables 1-2) and structural glycan knowledge as described in references 11,15-17.

[1] Registered mass in the first life stage the signal was observed.

[2] X, xylose; F, fucose; H, hexose; N, N-acetylhexosamine

[3] Relative expression of the registered masses within a life stage based on the area of the two highest isotope-peaks relative to the total area of all glycan signals.

[4] Assignment of glycan type and core modifications to the putative compositions are indicated with ‘v’.

[5] The total number of LeX- and LDN-antennae are indicated. Note that LDN, does not include fucosylated LDN.
