# Supplementary material for: Glycomic Analysis of Life Stages of the Human Parasite Schistosoma mansoni Reveals Developmental Expression Profiles of Functional and Antigenic Glycan Motifs
Source: Mol Cell Proteomics. 2015 Apr 16;14(7):1750–69. doi: 10.1074/mcp.M115.048280 (PMC4587318; doi:10.1074/mcp.M115.048280)
Supplement: Supplemental Data [file supp_M115.048280_mcp.M115.048280-9.pdf]

Supplemental Table 5. Terminal glycan motifs in *S. mansoni* glycoconjugates

Glycan motif	Glycan structure	Structure in symbols
Difucosyl / DF	Fucα1-2Fucα1-	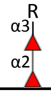
Trifucosyl / TF	Fucα1-2Fucα1-2Fucα1-	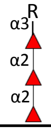
LacNAc /LN	Galβ1-4GlcNAcβ1-	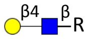
Lewis X / LeX	Galβ1-4(Fucα1-3)GlcNAcβ1-	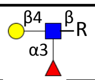
Pseudo Lewis Y / pseudo-LeY	Fucα1-3Galβ1-4(Fucα1-3)GlcNAcβ1-	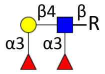
LacDiNAc / LDN	GalNAcβ1-4GlcNAcβ1-	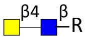
LDN-F	GalNAcβ1-4(Fucα1-3)GlcNAcβ1-	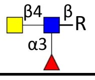
F-LDN	Fucα1-3GalNAcβ1-4GlcNAcβ1-	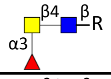
F-LDN-F	Fucα1-3GalNAcβ1-4(Fucα1-3)GlcNAcβ1-	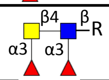
LDN-DF	GalNAcβ1-4(Fucα1-2Fucα1-3)GlcNAcβ1-	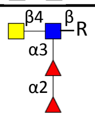
F-LDN-DF	Fucα1-3GalNAcβ1-4 (Fucα1-2Fucα1-3)GlcNAcβ1-	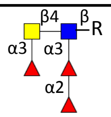
DF-LDN-DF	Fucα1-2Fucα1-3GalNAcβ1-4 (Fucα1-2Fucα1-3)GlcNAcβ1-	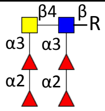
DF-LDN-TF	Fucα1-2Fucα1-3GalNAcβ1-4 (Fucα1-2Fucα1-2Fucα1-3)GlcNAcβ1-	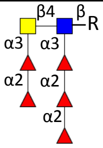
F-GlcNAc	Fucα1-3GlcNAcβ1-	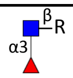
DF-GlcNAc	Fucα1-2Fucα1-3GlcNAcβ1-	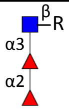
TF-GlcNAc	Fucα1-2Fucα1-2Fucα1-3GlcNAcβ1-	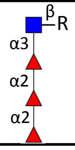
[a]Core-Xyl [a]Core(α3)-Fuc [a]Core(α6)-Fuc	Man(α1-3)(Manα1-6)(Xylβ1-2)Manβ1-4GlcNAcβ1-4(Fucα1-3)(Fucα1-6)GlcNAcβ1-	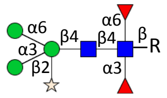

Red triangle, fucose; yellow square, N-acetylgalactosamine; blue square, N-acetylglucosamine; yellow circle, galactose; green circle, mannose; white star, xylose.

[a]N-glycan core-modifications which can be present separately or combined
